# Linear programming based gene expression model (LPM-GEM) predicts the carbon source for *Bacillus subtilis*

**DOI:** 10.1186/s12859-022-04742-7

**Published:** 2022-06-10

**Authors:** Kulwadee Thanamit, Franziska Hoerhold, Marcus Oswald, Rainer Koenig

**Affiliations:** Systems Biology Research Group, Institute for Infectious Diseases and Infection Control (IIMK), Jena University Hospital, Kollegiengasse 10, 07743 Jena, Germany

**Keywords:** Flux balance analysis, Mixed-integer linear programming, *Bacillus subtilis*, Carbon source, Transcriptomics, Constraint-based modeling, Thermodynamically infeasible loops

## Abstract

**Background:**

Elucidating cellular metabolism led to many breakthroughs in biotechnology, synthetic biology, and health sciences. To date, deriving metabolic fluxes by ^13^C tracer experiments is the most prominent approach for studying metabolic fluxes quantitatively, often with high accuracy and precision. However, the technique has a high demand for experimental resources. Alternatively, flux balance analysis (FBA) has been employed to estimate metabolic fluxes without labeling experiments. It is less informative but can benefit from the low costs and low experimental efforts and gain flux estimates in experimentally difficult conditions. Methods to integrate relevant experimental data have been emerged to improve FBA flux estimations. Data from transcription profiling is often selected since it is easy to generate at the genome scale, typically embedded by a discretization of differential and non-differential expressed genes coding for the respective enzymes.

**Result:**

We established the novel method Linear Programming based Gene Expression Model (LPM-GEM). LPM-GEM linearly embeds gene expression into FBA constraints. We implemented three strategies to reduce thermodynamically infeasible loops, which is a necessary prerequisite for such an omics-based model building. As a case study, we built a model of *B. subtilis* grown in eight different carbon sources. We obtained good flux predictions based on the respective transcription profiles when validating with ^13^C tracer based metabolic flux data of the same conditions. We could well predict the specific carbon sources. When testing the model on another, unseen dataset that was not used during training, good prediction performance was also observed. Furthermore, LPM-GEM outperformed a well-established model building methods.

**Conclusion:**

Employing LPM-GEM integrates gene expression data efficiently. The method supports gene expression-based FBA models and can be applied as an alternative to estimate metabolic fluxes when tracer experiments are inappropriate.

**Supplementary Information:**

The online version contains supplementary material available at 10.1186/s12859-022-04742-7.

## Background

Gaining insight into the metabolic fluxes can lead to a better understanding of how cells maintain their metabolic state and how they metabolically adapt to their microenvironment. It has led to astonishing discoveries such as considerably increased production yields after metabolic engineering [1–4], improved strain performance [5–7], and understanding various patho-mechanisms and identifying drug targets to cancer or diabetes [8–12]. In order to determine fluxes in metabolic pathways, metabolites are labeled with the specific ^13^C isotope and are traced over time employing mass spectrometry [1, 4, 6, 7, 9, 13–15], providing high accuracy and precision [16, 17]. However, these experiments are labor-intensive and costly [16, 18–20]. Besides this, constraint-based modeling (CBM) [21] has been applied to predict metabolic fluxes basing on flux balance analysis (FBA) [22]. FBA can be used to estimate metabolic fluxes without conducting such labeling experiments. Together with biologically reasonable assumptions as, e.g., bacteria or cancer cells aiming to maximize biomass production, fluxes of the metabolic reactions are derived from physiochemical constraints of their stoichiometry. FBA assumes a mass balance at a steady state for each (inner) metabolite. Additional constraints may be derived from thermodynamic constraints implying the directionality and enzyme capacity estimating a maximal enzymatic rate (V_max_). By this, FBA bypasses the need for reaction kinetic parameters facilitating to construct metabolic models on a genome scale without determining these experimentally demanding parameters [21–25]. It allows getting an estimate of the metabolic flux of interest leading to potential new hypotheses enabling the design of adapted experiments [2, 3, 10, 12, 26]. However, typically, utilizing only the stoichiometry of the reactions is insufficient to achieve good flux predictions. Hence, techniques were developed to add experimental data during model building [23, 27–33]. One of the most effective approaches was to integrate experimental omics data and specifically transcription profiles as they are not labor-intensive to generate on a systems view [34–36]. Though the data is not as direct as ^13^C tracer based data, it led to considerably good flux predictions [27, 28, 31–33]. Various methods have been developed to use gene expression data for metabolic network models. Most prominently, the approaches define qualitatively discretized  expressed/non-expressed reactions by setting a threshold as, e.g., implemented in the integrative Metabolic Analysis Tool (iMAT) [31, 33], the software Gene Inactivity Moderated by Metabolism and Expression (GIMME) [27], Probabilistic Regulation of Metabolism (PROM) [28] or the metabolic Context-specificity Assessed by Deterministic Reaction Evaluation (mCADRE) [32]). Although these context-specific model extraction methods successfully improved flux predictions compared to FBA not basing on expression data, finding suitable thresholds can be challenging. Moreover, employing defined thresholds disregard the fine-grained regulation of metabolism. To overcome this and provide better flux predictions, we propose a novel constraint-based approach considering the continuous nature of gene expression by embedding it in a linear model and applying three strategies to reduce thermodynamically infeasible loops.

Nutrition is essential for any cell to provide building blocks for maintenance and proliferation and to generate energy. Being able to identify carbon sources can support finding targets to treat microbial pathogens causing infectious diseases. Specifically, pathogenic micro-organisms are challenging to treat when hiding inside host cells. For example, osteomyelitis is an infection in the bone marrow and is mainly caused by *Staphylococcus aureus* [37–39]. It is a complex situation for treatment since antibiotics must penetrate the host cell or biofilm to eradicate the bacteria. For such a condition, ^13^C tracing experiments are difficult [19] as the observed tracing components may not be adequately traced back to the production/consumption of the host cell or the pathogen, and FBA may be a good alternative. We developed our method to predict nutritional uptakes, which may, in a future study, be applied to such a rather complex cellular situation in which a simple tracer analysis is difficult to perform. As a case study, we demonstrated our modeling concept and predicted the nutrition of the well-studied bacterium *Bacillus subtilis* (*B. subtilis*) using publicly available gene expression data studied at different carbon source conditions [13, 40].

## Methods

### Data assembly

#### Experimental data of the eight-carbon-source study (first dataset)

Published microarray gene expression data of the *B. subtilis* strain BSB1 was used. BSB1 is a tryptophan prototrophic derivative of strain 168. The data was taken from the original publication (Table S2 from [40]). The data based on tilling arrays covering the whole genome of *B. subtilis* *168* [40]. *B. subtilis* was grown in minimal medium in eight different carbon source conditions (glucose, fructose, gluconate, glutamate/succinate, glycerol, malate, malate/glucose, pyruvate) [40]. To validate our model, we used metabolic flux data from ^13^C isotope labeling experiments of the same eight carbon source conditions (Table S4 from [14]). In the following, this data will be denoted as the first dataset.

#### Experimental data of the nutritional-shift study (second dataset)

To validate our model with a separate, unknown dataset, we used publicly available gene expression and ^13^C tracer based metabolic flux data from a time-series experiment of two nutritional shifts, i.e., the shift from glucose to glucose plus malate and the shift from malate to malate plus glucose [13]. Gene expression and ^13^C metabolic flux data were generated using the same experimental protocol as for the first dataset. *B. subtilis* was grown in minimal medium on a single carbon substrate until an OD_600_ of 0.5 was achieved. Then, the other substrate (glucose or malate) was added to the culture to assess the bacterial behavior at 0 (before the addition of the other substrate), 5, 10, 15, 25, 45, 60, and 90 minutes after the other substrate was added. Both gene expression and ^13^C metabolic flux data were taken from the BaSysBio database (https://basysbio.ethz.ch/openbis/basysbio_openbis.html). This data is denoted as the second dataset in the following.

### Data pre-processing

We used the gene expression data of the first and second datasets. It had been pre-processed by computing the median of the estimated transcription signal of all probes assigned to one corresponding gene [13, 40]. The gene expression data in the second dataset had been further processed by quantile normalization [13]. All gene expression levels were provided after log2 transformation [13, 40]. In order to obtain the gene symbols, BSU identifiers were matched with gene symbols using bioDBnet, version 2.1 [41]. In the first dataset, each condition contained three biological replicates, and we used all of them. For most of the time points of the second dataset, three biological replicates were available. The rest had two biological replicates. For each condition/time point, gene expression levels across the available replicates were averaged. To map gene expression values to proteins and reactions, we used the gene-protein-reaction (GPR) mapping from the original publication of Chubukov et al. [14] and the metabolic network of *B. subtilis* *168* available from the BiGG Models database [42] (BiGG ID iYO844) [43]. We compared the mappings with the information from UniProt [44] and KEGG [45–47] and corrected it if stated otherwise in these databases. Additionally, we found literature about two more genes (lrgA, lrgB) coding for a pyruvate transporter and added them to the corresponding reaction in the GPR mapping [48]. The GPR mapping we used is provided in Additional file 1: Table S1. ^13^C metabolic flux data from Chubukov et al. [14] and Buescher et al. [13] were used as published without further processing.

### Model building

#### Building the metabolic model

To develop a mixed-integer linear programming based model, we transferred the iYO844 model of *B. subtilis* from Matlab to R (stoichiometric matrix, lower and upper bounds, reversibility, metabolite, and reaction names). To efficiently compare prediction results from our approach with the ^13^C metabolic flux data, we determined if we could fit the ^13^C metabolic flux data to the metabolic model in R. The solution from the ^13^C model needed to be a feasible solution complying with all set constraints. However, initial trials showed that we could not find any solution in the solution space when we tried to fit flux values from the ^13^C metabolic flux data allowing only one exchange reaction flux to be non-zero, i.e., from the specific transporter of the corresponding carbon source. In turn, it was possible to find a feasible solution when we also allowed fluxes from other exchange reactions besides the exchange reaction of the corresponding carbon source to enter the system. Although the solution was found, the flux values from other exchange reactions were substantially high, which was unrealistic. Hence, we set up an optimization problem to find a reasonable boundary for each of these exchange reactions by letting the solution deviate from ^13^C metabolic flux data by maximal 0.1. After optimization, we obtained a sum of fluxes from other exchange reactions for each different condition. We then compared these values and applied the lowest possible value (sum of fluxes = 0.688) restricting the influx of all other metabolites (being not the metabolite of the corresponding condition) into the cell. The list of all exchange reactions besides the designated carbon sources of the corresponding minimal medium is provided in Additional file 1: Table S2. For a fair comparison between LPM-GEM and the benchmark methods (parsimonious enzyme usage Flux Balance Analysis (pFBA) [49], the integrative Metabolic Analysis Tool (iMAT) [31, 33], and metabolic Context-specificity Assessed by Deterministic Reaction Evaluation (mCADRE) [23]), the bound of these exchange reactions was consistently opened across all methods providing the best possible solution space to achieve correct flux predictions. Restricting fluxes of these exchange reactions was adjusted according to the flexibility of each method. While it was possible to limit the bound of these exchange reactions in pFBA, we received infeasible solutions for iMAT and mCADRE. Thus, we allowed no restriction for these two methods as it was found to be necessary for their implementation.

#### Defining the set of reactions for the optimization criterion

As described below, we benchmarked our models with a well-defined gold standard, i.e., flux values based on the ^13^C labeling data from the original publication. This gold standard data was available for 40 reactions, mostly covering central energy metabolism [14]. Hence, these reactions were used for the optimization of our metabolic model explained in the next section. These reactions are called core reactions in the following. To improve the model predictions, we added a selection of further reactions to the optimization function of our model, called associated reactions in the following. We added associated reactions following three criteria, (1) they needed to be reactions that were directly connected (via an exchanging metabolite) to the core reactions in central energy metabolism or amino acid biosynthesis, (2) important metabolites in glycolysis or tricarboxylic acid (TCA) cycle (e.g., glyceraldehyde 3-phosphate, pyruvate, oxaloacetate, α-ketoglutarate) are substrates or products of these reactions, and (3) at least one of the associated genes to the reactions needed to be differentially expressed in at least one out of the eight carbon sources of the first dataset when compared to the expression of *B. subtilis* in the control medium (*B. subtilis* grown in LB medium) [13]. For this, T-tests were performed comparing the expression value of the corresponding gene in each specific carbon source condition *versus* its expression in the control medium. The Benjamini-Hochberg method was used to correct for multiple testing across all genes [50]. The p-value cutoff was 0.05. By this, we assembled 119 genes and 138 reactions in total (Additional file 1: Table S1).

### Formulating the optimization criterion

We assumed that the metabolic flux correlates linearly with the expression value of the gene coding for the responsible enzyme of the corresponding reaction (Additional file 1: Figure S1). We also tested more complex transformations but found no improvement/reduction of the residuals of the regression models of the according transformed expression data with the ^13^C flux data (Additional file 1: Figure S2). We linearly mapped gene expression values to predicted fluxes formulated within the following optimization problem.

Let $v_{ri,c}^{fit}$ represent a gene expression-based flux for reaction $ri$ ($ri$ is a reaction that is part of the core or associated reactions) in condition $c$. $v_{ri,c}^{fit}$ is based on information from gene expression data and the flux range,

1 $$v_{ri,c}^{fit} = V_{ri}^{min} + \left( {\overline{g}_{ri,c} - g_{ri}^{min} } \right)\left[ {\frac{{\left( {V_{ri}^{max} - V_{ri}^{min} } \right)}}{{\left( {g_{ri}^{max} - g_{ri}^{min} } \right)}}} \right]$$

where $\overline{g}_{ri,c}$ is the averaged gene expression value of the gene associated with reaction $ri$ in condition $c$. $g_{ri}^{min}$ is the minimum gene expression value across all conditions of the gene associated with reaction $ri$, $g_{ri}^{max}$ is the maximum gene expression value. $V_{ri}^{min}$ is the minimum possible flux and $V_{ri}^{max}$ is the maximum possible flux across all conditions obtained from flux variability analysis (FVA, see below) for core reactions (CR) and associated reactions (AR).

Under the FBA framework, we assumed that the metabolism is in a steady state. Hence, there is no accumulation of mass, which means no change of metabolite concentration over time. $S_{r}$ is the stoichiometric matrix of the metabolic network, $v_{r,c}$ represents the predicted flux for reaction $r$ ($r$ is any reaction in the network) in condition $c$ in the metabolic network. The variable $v_{r,c}$ must satisfy the constraints from the stoichiometry, as well as lower $lb_{r}$ and upper bounds $ub_{r}$, i.e.,

2 $$S_{r} \cdot v_{r,c} = 0$$

3 $$lb_{r} \le v_{r,c} \le ub_{r}$$

Subject to constraints (1) to (3), we formulated the optimization problem by

4 $${\text{Minimize}}\;\mathop \sum \limits_{ri,c} w_{ri} \cdot \left| {v_{ri,c} - v_{ri,c}^{fit} } \right| + \alpha \mathop \sum \limits_{ro,c} v_{ro,c}$$

5 $$w_{ri} = \left\{ {\begin{array}{*{20}l} {\frac{1}{{V_{ri}^{weight} }},} \hfill & {\forall ri \in CR} \hfill \\ {\frac{1}{{V_{ri}^{weight} + 100}},} \hfill & {\forall ri \in AR} \hfill \\ \end{array} } \right.$$

The formulated objective function is a trade-off between two optimization criteria. The first term, $\mathop \sum \limits_{ri,c} w_{ri} \cdot \left| {v_{ri,c} - v_{ri,c}^{fit} } \right|$, minimizes an error between the predicted flux $v_{ri,c}$ and the gene expression-based flux $v_{ri,c}^{fit}$. The weight $w_{ri}$ is introduced to adjust the term through equation (5). The predicted flux $v_{ri,c}$ was adjusted by averaging the gene expression values using the weight $w_{ri}$ for each gene encoding the reaction $ri$.$V_{ri}^{weight}$ was obtained by selecting the maximum of absolute values of the maximum or minimum flux from the FVA derived maximal flux values. The weight was set as the reciprocal of this value to make reactions with small and high variances of fluxes equally important to the objective function. The associated reactions were down-weighted by adding the constant +100 in the denominator. Moreover, we discarded reactions for which $V_{ri}^{min}$ and $V_{ri}^{max}$ were zero. This resulted in lower numbers of reactions leading to 98 reactions basing on 116 genes (Additional file 1: Table S3).

The second term in formula (4), $\alpha \mathop \sum \limits_{ro,c} v_{ro,c}$, aims to minimize a sum of all predicted fluxes $v_{ro,c}$ from reactions being not CR nor AR coping for the problem of obtaining thermodynamically infeasible loops. To obtain an appropriate $\alpha$ value, the sum of sums of absolute values of fluxes $v_{ro,c}$ across all conditions, $\mathop \sum \limits_{i = 1}^{c} \mathop \sum \limits_{ro,c} \left| {v_{ro,c} } \right|$, and a total model mapping discrepancy were assessed for each $\alpha$ variation (Additional file 1: Figure S3). The total model mapping discrepancy $d$ is a coefficient used to measure an overall deviated distance between $v_{ri,c}$ and $v_{ri,c}^{fit}$ from all reactions $ri$ across all conditions. It reflects how good $v_{ri,c}$ resembles $v_{ri,c}^{fit}$ and was derived by

6 $$d = \mathop \sum \limits_{i = 1}^{c} \mathop \sum \limits_{ri,c} \left| {v_{ri,c} - v_{ri,c}^{fit} } \right|$$

After comparing the sum of sums of absolute values of fluxes $v_{ro,c}$ and the total model mapping discrepancy from different $\alpha$ values, the value of 0.01 was set and selected since the sum of sums of absolute values of fluxes $v_{ro,c}$ was considerably reduced while the total model mapping discrepancy was only moderately increased (Additional file 1: Figure S3).

A biomass constraint obtained from the growth rate was set for each condition *c* based on the data from Chubukov et al. and Buescher et al. [13, 14]. The biomass constraint was implemented as a lower limit by

7 $$c_{biomass, c}^{T} v_{biomass, c} \ge B_{c}$$

where $c_{biomass, c}^{T}$ is the transpose of the biomass reaction coefficient, $v_{biomass, c}$ is the predicted flux for the biomass reaction in condition $c$, and $B_{c}$ is the scalar product from the biomass production for condition $c$, as given by Chubokov et al. and Buescher et al. In our implementation, we opened the lower bounds for all eight carbon source exchange reactions to allow influxes of any possible carbon source during learning of the model based on the corresponding gene expression profiles. We took the maximum substrate rate (negative lower bounds) reported in Chubukov et al. [14] across all conditions for each carbon source. We set these lower bounds of all eight carbon source transporter reactions to the minimum values for all conditions. This setting made sure to predict carbon sources without prior knowledge of the carbon source in the certain carbon source condition.

#### Reducing the search space employing Iterative Feasible Flux Space Reduction (IFFPR)

To correctly map the expression data to the metabolic flux, we needed a realistic estimate of the lower and upper bounds for the reactions in the model. At different steady-state conditions, the feasible minimum and maximum flux within the solution space can differ from the initially set lower and upper bounds of each reaction. FVA is a well-known technique to determine flux ranges [51]. We applied FVA to determine the minimum and maximum possible fluxes as follows. For each reaction $r$ in condition $c$, in FVA, it is assumed that the metabolic network is in a steady state and the stoichiometry is fulfilled, as referred to equations (2) and (3). FVA minimizes and maximizes the flux $v_{r,c}$ to find an upper and lower bound for the respective reaction satisfying the FBA constraints (formula (2) and (3)).

In general, doing this for every reaction should narrow down the flux range of each reaction. However, we observed that the boundaries did not differ substantially after performing FVA for every core and associated reaction. To further reduce the solution space and limit its flexibility, we developed an iterative approach. The approach was part of the training scheme to narrow down the flux ranges; hence, we applied this only to the training data. The method was termed Iterative Feasible Flux Space Reduction (IFFPR). A figure of the workflow is given in the Additional file 1: Figure S4. IFFPR works as follows:

- $V_{ri}^{max}$ and $V_{ri}^{min}$ are acquired by the above-described FVA for each reaction.
- Absolute values of $V_{ri}^{max}$ and $V_{ri}^{min}$ from each reaction are compared and the maximum of these values used as the representative maximal bound for this reaction. Representative maximal bounds from all reactions are used to rank the reactions. The reaction with the highest value is placed at the top position (i = 1).
- The first reaction $ri$, with i = 1 is selected.
- $V_{ri, P}^{max}$ =$V_{ri}^{max}$ and $V_{ri, P}^{min}$= $V_{ri}^{min}$ is set.
- 8 $$V_{ri, C}^{max} = 0.5*V_{ri, P}^{max}$$
  9 $$V_{ri, C}^{min} = 0.5*V_{ri, P}^{min}$$
  in which $V_{ri, C}^{max}$ is the new maximal possible flux for the current iteration $C$, $V_{ri, C}^{min}$ is the new minimal possible flux for the current iteration $C$. $P$ is used to indicate the previous iteration. $V_{ri, P}^{max}$ and $V_{ri, P}^{min}$ are reduced by half every iteration. Since the reaction can be unidirectional or bi-directional, $V_{ri, P}^{max}$ and $V_{ri, P}^{min}$ can have similar or different signs. Equations (8) and (9) are applied to reduce $V_{ri, P}^{max}$ and $V_{ri, P}^{min}$. If $V_{ri, P}^{max}$ and $V_{ri, P}^{min}$ have the same sign, either equation (8) or (9) is used depending on the sign aiming to reduce the flux range.
- $V_{ri, C}^{max}$ and $V_{ri, C}^{min}$ are applied as $V_{ri}^{max}$ and $V_{ri}^{min}$ in formula (1) mapping gene expression values to flux.
- After optimizing the objective function in formula (4), the total model mapping discrepancies are compared between the previous and the new iteration. If the total model mapping discrepancy from the previous run is greater, the algorithm proceeds with the next iteration and proceeds with step e).
- The inner iterative process terminates for reaction i. The next reaction in the list is selected by setting i = i+1, and the algorithm proceeds with step d).
- The algorithm terminates if the total model mapping discrepancy becomes stable, or all reactions are processed.

We terminated the process before the algorithm reached the end of the list. As the algorithm processed around 80% of the reactions in the list, the total model mapping discrepancy became stable (Fig. 2). This was explainable as the rest of the reactions (~ 20%) already showed narrow flux ranges (Additional file 1: Table S4). Reducing the flux ranges for these reactions could not influence the total model mapping discrepancy any further but only cost more computational time (Fig. 2). Hence, the algorithm was stopped, and the sets of $V_{ri, C}^{max}$ and $V_{ri, C}^{min}$ were obtained.

### Reducing the number of thermodynamically infeasible loops

In constraint-based modeling, the thermodynamic loop law can get violated. The loop law is similar to Kirchhoff’s second law for electrical circuits [29]. It states that at steady state there must not be any closed cycle or loop in the metabolic network with a non-zero net flux. Such loops would disregard the second law of thermodynamics and are hence thermodynamically infeasible. The problem of avoiding thermodynamically infeasible loops (TIL) can be solved by imposing thermodynamic constraints such as standard-state free energy of reaction into the optimization. However, it is very challenging to acquire this information for the whole metabolic network as well as to implement it in optimization-based computations [30].

To solve this problem within the FBA framework, Schellenberger et al. [30] introduced a method called loopless-COBRA (ll-COBRA). Il-COBRA removes TIL from the network by integrating thermodynamic constraints obtained from flux directionality which readily exists inside every metabolic network with FBA. Although the problem becomes less complex, it is still computationally intensive. In order to speed up the process to remove TIL, we developed a novel iterative procedure to detect and remove TILs called REDucing the number of Thermodynamically Infeasible Loops (RED-TIL). After obtaining flux prediction results from the mapping procedure (see Materials and methods, Formulating the optimization criterion), the results were used as an input for a MILP problem to identify TILs and exclude them.

External reactions are not regarded. Applying a maximal flux value threshold (threshold = 0.01) for TIL to be allowed, the set of reactions $supp\left( v \right)$ known as the support of $v$ is assembled. $supp\left( v \right)$ contains a subset of the internal reactions ($v \ge$ 0.01). We applied the value of 0.01 as a trade-off between CPU time and reasonable results. Next, an optimization problem is put up to determine the length of a minimum-containing TIL in the solution by

10 $${\text{Minimize}}\;\mathop \sum \limits_{r} \lambda_{r}$$

subject to

11 $$\mathop \sum \limits_{r} S_{r} \cdot \lambda_{r} = 0$$

12 $$\lambda_{r} \ge inFC_{r}$$

13 $$\mathop \sum \limits_{r} inFC_{r} \ge 2$$

14 $$inFC_{r} \in \left\{ {0, 1} \right\}$$

where $\lambda_{r}$ is the flux of reaction $r$ ($\forall r \in supp\left( v \right)$), $S_{r}$ is a stoichiometric matrix of the metabolic network with metabolites and reactions, $inFC_{r}$ is a binary variable which equals to 1 for a reaction that is involved in the potential TIL. In a system that contains a TIL, there must be at least two reactions involved enforced by equation (13). If a solution of the problem put up by equations (10)–(14) is found, a TIL (of length k) is detected. A constraint is added not allowing this TIL by

15 $$\mathop \sum \limits_{i = 1}^{k} inFC_{{r_{i} }} \le k - 1$$

Equation (15) forces the algorithm to search for a solution that puts at least one of these variables $inFC_{{r_{1} }} ,inFC_{{r_{2} }} , \ldots , inFC_{{r_{k} }}$ to 0 which leads to the TIL to be discarded from the solution. In the next optimization iteration, the mapping procedure is re-optimized using equations (1) to (5) together with the newly added constraint from equation (15), followed by finding new TIL employing the MIP problem described by equations (10)–(14). The algorithm stops when no TIL above the threshold can be found.

### Workflow for validating the model

**Fig. 1:**
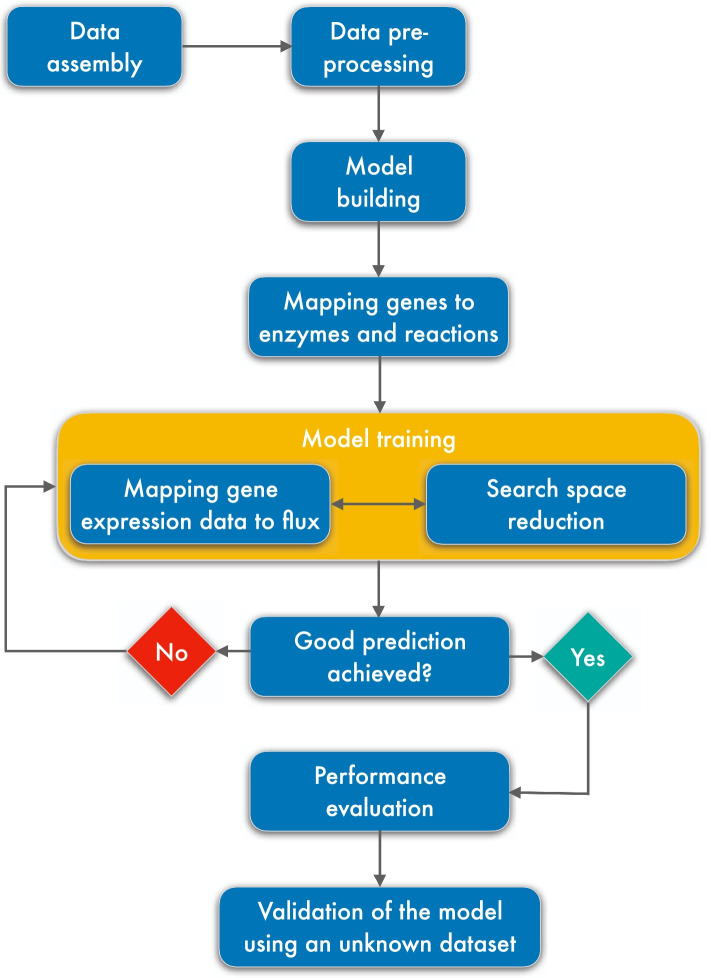
The workflow.

The overview of the entire process is illustrated in Fig. 1. We started by learning the model based on gene expression data as explained above, obtaining the best parameter setting. Then, we predicted the primary carbon source for each of the eight carbon source conditions by selecting the transporter (one out of eight potential transporters) with the highest flux in the corresponding condition. The prediction was validated by comparing it to the known carbon source of the according condition. Besides, we compared the flux predictions of the 40 core reactions with the flux of the original publication [14] derived by ^13^C tracer analysis and quantified the similarity by Pearson’s correlation and normalized error as described in the publication of Machado and Herrgard [52]. Furthermore, the model was applied to an unknown dataset, i.e., the data from the time series on spiked glucose on malate and spiked malate on glucose medium as described above.

### Implementation of the benchmarking methods

To benchmark LPM-GEM, we compared its prediction performance with three well-established methods, i.e., pFBA [49], iMAT [31, 33], and mCADRE [23]. For the implementation of pFBA, we followed the tutorial provided by the Cobra toolbox [23]. The same metabolic model used by LPM-GEM was used, and minimal lower bounds for all eight carbon source exchange reactions and biomass production information were taken from Chubukov et al. [14] to get flux predictions from the method. As iMAT is implemented inside the Cobra toolbox [23], we followed a tutorial from the iMAT protocol [31, 33] to ensure the correct implementation of the method. We employed the same metabolic model, the same gene expression data for the eight different carbon source conditions [40], and GPR mapping as in LPM-GEM. In line, the minimal lower bounds for all eight carbon source exchange reactions were taken from Chubukov et al. [14]. We set upper (a cutoff for non-zero flux reactions) and lower thresholds (a cutoff for zero flux reactions) equal to +/− 0.3 SD from gene expression values following the suggested iMAT discretization process [31, 33, 53]. To implement mCADRE, we followed a tutorial provided by a publication of mCADRE [32]. As for LPM-GEM and iMAT, we employed the same metabolic model, gene expression data for the eight different carbon source conditions [40], and GPR mapping. Minimal lower bounds for all eight carbon source exchange reactions were again taken from Chubukov et al. [14]. In order to binarize gene expression data and calculate ubiquity scores for mCADRE, the gene expression data was binarized by considering transcripts with expression values less than the 75^th^ percentile of expression values in the matrix as zero and as one otherwise [32, 54]. Also, other thresholds were tested but led to worse results (Additional File 1: Table S5). Then, ubiquity scores for genes were determined by calculating the number of samples with expressed genes divided by the total number of samples for each specific gene, as described in the original publication of mCADRE [32, 54].

### Implementation and statistics

All analyses were performed using R version 3.3.3 (www.r-project.org). The Cobra toolbox version 3.0 and Matlab version R2019a (www.mathworks.com) were used to obtain the initial stoichiometric matrix, lower and upper bounds, reversibility information, metabolite and reaction names from the initial metabolic network, and benchmarking flux predictions from iMAT, mCADRE and pFBA (see Results). All further analysis was performed using R. The Gurobi optimizer version 9.0.2 (www.gurobi.com) was used to solve mixed-integer linear programming problems. For assessing the carbon sources, z-scores were calculated for each transporter of the eight (eight-carbon-source study) or two (nutritional-shift study) different carbon sources across all conditions as a means to compare the results of the predictions across all the corresponding transporters. This enabled us to compare also transporters with lower differences in their fluxes among the different conditions to transporters with higher differences.

## Results

### Reducing the search space following three strategies improves flux predictions

Thermodynamically infeasible loops are problematic for constraint-based modeling leading to incorrect flux distributions [29, 30, 55]. Obtaining a realistic context-specific model was a main goal when integrating transcription profiles for building the metabolic model. To base our analyses on solutions that are thermodynamically feasible, we reduced TILs following three strategies, i.e.,

1. Reducing the search space employing Iterative Feasible Flux Space Reduction (IFFPR),
2. Applying our method RED-TIL, and
3. Penalizing high flux contributions of non-core and non-associated reactions.

All strategies improved our models as described in the following (the improvement by strategy (3) is described in a lower section (see Adding a penalty for the sum of fluxes led to improved predictions).

#### Improving the model by iterative feasible flux space reduction (IFFPR)

**Fig. 2:**
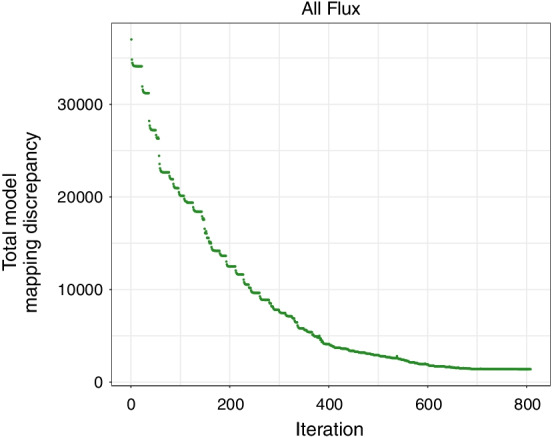
The total model mapping discrepancy calculated over all eight conditions (at $\alpha$ = 0.01, the penalty parameter  α is described below) is shown with respect to the number of iterations of the search space reduction algorithm (IFFPR). As the algorithm proceeds to the end of the list of reactions (> 600 iterations), the total model mapping discrepancy does not further decrease.

For all fitted reactions (core and associated reactions) of the model, we performed flux variability analysis (FVA) to reduce the maximal and minimal flux boundaries. However, we observed that for many reactions, the resulting flux ranges did not substantially differ from the original upper and lower bounds. These high flux ranges were expected not to reflect realistic situations and may facilitate using TILs by the optimization procedure when fitting the model to the transcription profiles. Hence, we developed a new method to iteratively reduce the flux boundaries from FVA by comparing the discrepancy between the fluxes derived from the expression values and the optimal feasible flux when obtaining newly adjusted maximal ($V_{ri}^{max}$) and minimal ($V_{ri}^{min}$) possible fluxes for every fitted reaction. In the following, this discrepancy is denoted as the “total model mapping discrepancy”. This led to new, considerably reduced bounds (Additional file 1: Table S4). Notably, the total model mapping discrepancy decreased considerably by 95.65% from the original discrepancy (Figure 2). All flux predictions with and without employing IFFPR are listed in Additional file 1: Tables S6 and S7.

In summary, reducing the search space improved integrating the experimental data more efficiently observed by a decreased total model mapping discrepancy.

#### Reducing thermodynamically infeasible loops within the FBA model

ll-COBRA is a well-established and efficient method to remove TIL in constraint-based modeling efficiently. ll-COBRA generates one large MILP problem finding an optimal solution while enforcing fluxes from internal reactions participating in all detected cycles to be zero [30]. The method is very powerful but computationally demanding. Hence, we developed a novel method (RED-TIL) based on MILP iteratively removing TIL to solve the same problem. Our iterative approach required considerably less running time. RED-TIL solves an FBA problem, identifies and removes iteratively from bottom-up TIL in the solution space. The process is repeated until no TIL above a certain threshold (threshold = 0.01) is detected. To compare these approaches, we implemented both methods using the same R programming environment and the same numerical solver, yielding very similar solutions (Pearson’s correlation coefficient r = 0.96, Additional file 1: Figure S5 and Table S8). Even though we yielded similar results, we observed different running times. Explicitly, when we performed FVA for every reaction in the network (1250 reactions equal to 2500 iterations per condition), RED-TIL needed 12.89 hours for all eight conditions while ll-COBRA required 42.83 hours (Additional file 1: Figure S6 shows running times for each carbon source).

In summary, for removing thermodynamically infeasible loops, our new approach RED-TIL led to similar models in much faster running time when compared to a well-established, commonly used method.

#### LPM-GEM identifies the correct carbon sources

**Fig. 3:**
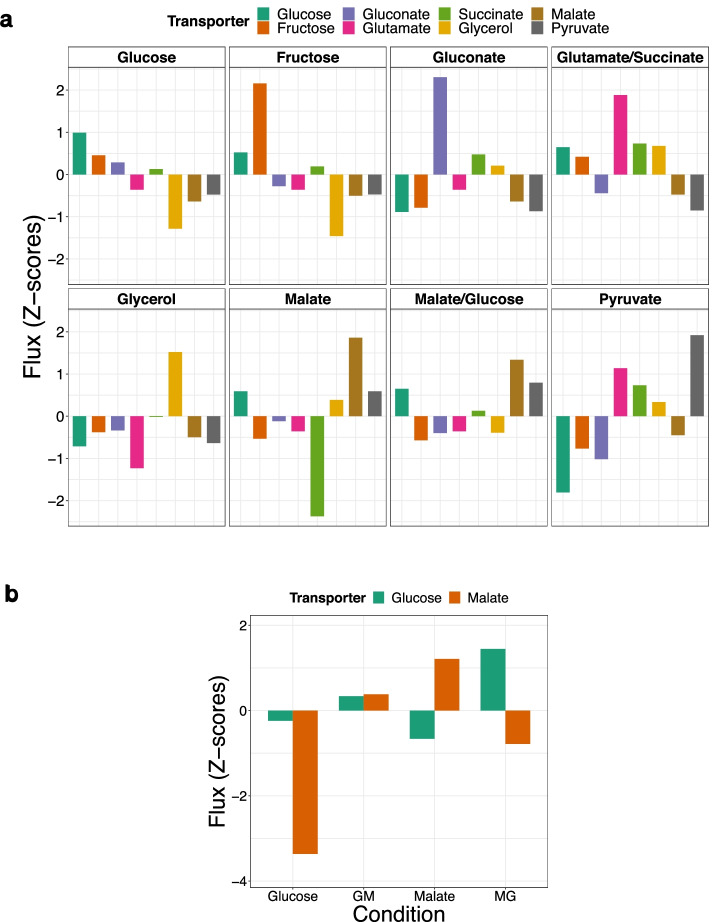
**a** In each panel, the bars show the z-scores of the predictions of the carbon source transporters are shown (of the eight-carbon-sources study). The headers of the panels indicate the true carbon source of the respective condition; **b** Prediction of the carbon source for the nutritional shift. GM: glucose to glucose plus malate, 90 min after adding malate; MG: malate to malate plus glucose, 90 min after adding glucose. For (**a**) and (**b**), a higher z-score indicates a higher probability for a specific carbon source.

We mapped gene expression levels onto the reactions by a regression approach (see Materials and Methods) and generated context-specific metabolic models employing RED-TIL and IFFPR. This was done across each of the eight carbon sources (glucose, fructose, gluconate, glutamate, succinate, glycerol, malate, pyruvate). Besides the core reactions, for which ^13^C metabolic flux data was available, we considered “associated reactions” for optimally fitting the optimal solution to the transcription profiles of the corresponding coding genes. The “associated reactions” were neighbors of the core reactions and were assumed to be important for the carbon source prediction (see Materials and Methods, Defining the set of reactions for the optimization criterion). After optimization, we assessed our prediction results of the eight transporter reactions (glucose, fructose, gluconate, glutamate, succinate, glycerol, malate, pyruvate) to identify the major carbon source for each condition. For this, we compared the z-scores of the corresponding carbon source transporters in each condition to predict the primary (highest z-score) and secondary (second highest z-score) carbon sources (Fig. 3a, Additional file 1: Figure S7a). For carbon source conditions with only one carbon source, all predictions were correct (n = 6, glucose, fructose, gluconate, glycerol, malate, pyruvate). For the two carbon sources, which consisted of two carbon sources (glutamate/succinate, malate/glucose), the primary carbon source was also correctly predicted. The secondary carbon source (succinate) was predicted correctly for the carbon sources glutamate and succinate. However, for the carbon sources malate and glucose, pyruvate was predicted as a second carbon source instead of glucose. Notably, the z-score of the glucose transporter was only slightly below the z-score of the pyruvate transporter (Fig. 3a).

Overall, our method could well predict the carbon sources based on gene expression profiles of the respective conditions.

#### Adding a penalty for the sum of fluxes led to improved predictions

In addition to optimizing the fitting of gene expression to the fluxes of the reactions, we also considered minimizing the sum of all predicted fluxes from reactions which were either core reactions or associated reactions to restrict the optimizer employing thermodynamically infeasible loops (see Materials and Methods, Formulating the optimization criterion). We tested different strengths of the penalty (ranging from $\alpha$ = 0 (no penalty) to α = 10 (high penalty)) gauging between low total model mapping discrepancy and a low total sum of fluxes $v_{ro,c}$ across all conditions before applying IFFPR and RED-TIL (Additional file 1: Figure S3). We selected $\alpha$ = 0.01 serving as a suitable trade-off parameter preventing high fluxes as we observed a considerable great decrease from the total sum of fluxes (compared to $\alpha$ = 0) while leaving the total model mapping discrepancy moderate.

### LPM-GEM outperforms existing methods

For benchmarking, we compared our method with three other well-known methods (iMAT [31, 33], mCADRE [32], and pFBA [49]). We constructed the metabolic models using the same metabolic network of *B. subtilis,* the same gene expression profiles from the eight carbon source conditions (glucose, fructose, gluconate, glutamate/succinate, glycerol, malate, malate/glucose, pyruvate), and we selected the same core reactions for iMAT and mCADRE. For pFBA, no gene expression data was used as the method does not require them. We followed the standard protocols from these three methods (see Materials and Methods) and compared their flux predictions to the gold standard (^13^C tracer derived fluxes), i.e., to all 40 reactions [14] for which ^13^C data was available.

Among all four approaches, only pFBA failed to give flux predictions according to the different biomass constraints from each condition. We got the same models across all eight conditions in this setting. The method could only distinguish different flux profiles when we restricted the specific one or two carbon sources corresponding to each specific condition to enter the system. Even though this was not the principal aim of our study (our principal aim was to predict the carbon sources and not the metabolic fluxes based on the prior knowledge of the carbon sources), we assessed the flux prediction performance based on biomass and the known carbon sources. We calculated Pearson’s correlation coefficients (r) and normalized errors [52] between pFBA predictions and the gold standard for these 40 reactions. The average Pearson’s correlation coefficient is 0.68 (SD = 0.32), and the normalized error is 0.90. Table S9 in Additional file 1 lists the predicted fluxes. Pearson’s correlation coefficients from these 40 reactions are provided in Table S10. We also compared the carbon source predictions assessed by the fluxes from the corresponding transporters. pFBA identified all six single carbon sources correctly, but the approach could only predict one out of two from two-carbon sources (Additional file 1: Figures S8–S9). Although pFBA performed well in the new setting, it did not suit the purpose of this study aiming to predict the carbon sources without prior knowledge of the used carbon sources. Hence, the method could not be compared with the other methods and was excluded from further comparisons.

**Fig. 4:**
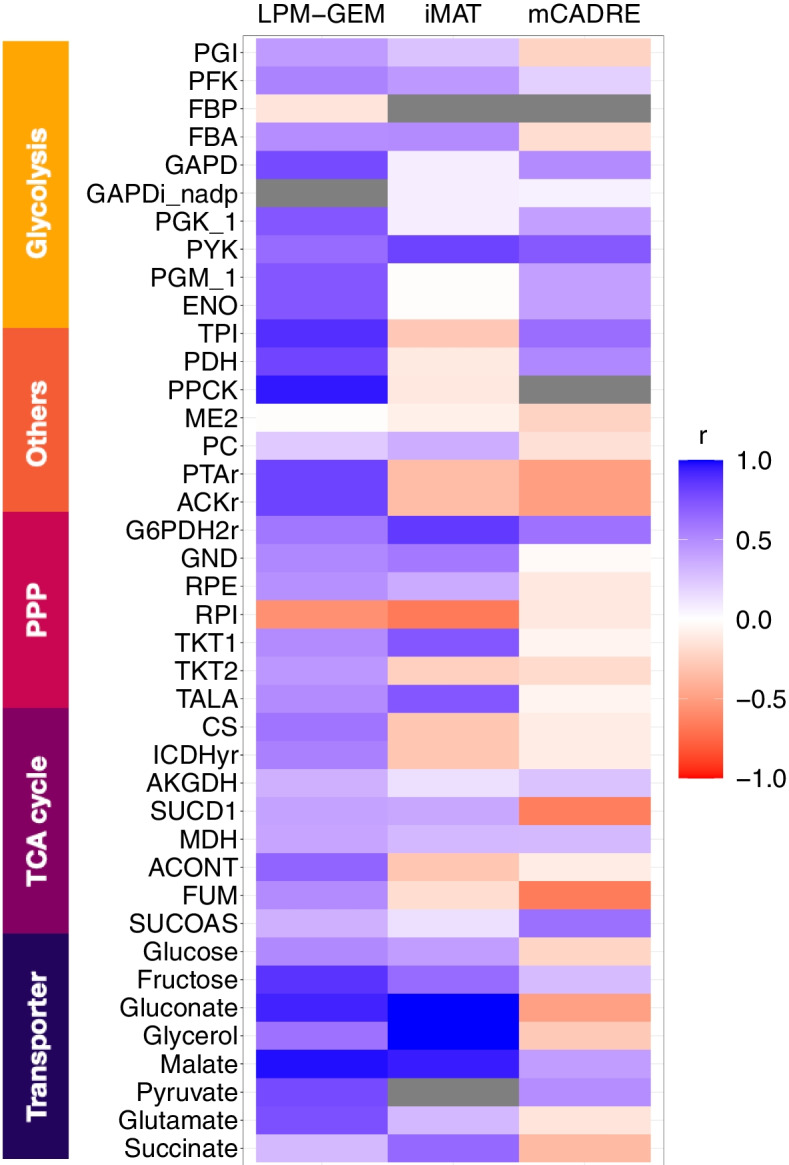
Prediction performance of our approach (LPM-GEM), iMAT, and mCADRE. For all 40 core reactions for which gold standard data (from ^13^C tracer analysis) was available, the Pearson’s correlation coefficients between the predicted fluxes and the fluxes from the gold standard are shown (grey: fluxes are predicted to be zero in every condition); PPP: pentose phosphate pathway, TCA: tricarboxylic acid.

**Fig. 5:**
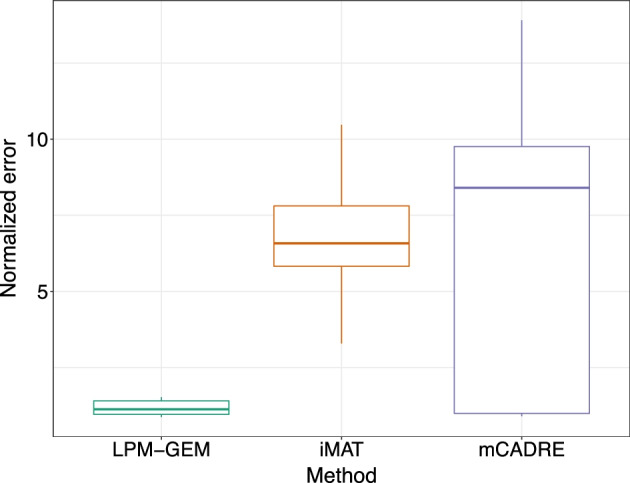
Comparison of normalized errors from different methods. Normalized errors of LPM-GEM, iMAT, and mCADRE from eight different conditions are shown in the square root scale (the normalized error is the Euclidean distance between ^13^C metabolic flux values and predicted flux values of the specific condition divided by the magnitude of ^13^C metabolic flux values of the same condition [52])

We then compared the flux predictions of LPM-GEM with iMAT, and mCADRE. The scatterplot of the predicted fluxes of all three methods (iMAT, LPM-GEM, and mCADRE) *versus* the measured fluxes (^13^C metabolic fluxes) is provided in Additional file 1: Figure S10. All available flux predictions are listed in Additional file 1: Table S7 and Tables S11- S12. To assess the flux prediction performance from all methods, we also calculated Pearson’s correlation coefficients and normalized errors [52] between predictions and the gold standard for these reactions as we did previously. On average, our method outperformed iMAT and mCADRE (averaged Pearson’s correlation coefficient, LPM-GEM: r = 0.55 (SD = 0.31), iMAT: r = 0.22 (SD = 0.44), mCADRE: r = 0.04 (SD = 0.39)). Pearson’s correlation coefficients for these reactions between the predictions and the gold standard are shown for LPM-GEM, iMAT, and mCADRE in Fig. 4 (and listed in Additional file 1: Tables S13-S15). For normalized errors, LPM-GEM also showed a lower normalized error than iMAT and mCADRE (averaged normalized error, LPM-GEM: 1.46, iMAT: 49.09, and mCADRE: 68.04). The normalized errors from eight different conditions are shown in Fig. 5. Next, we compared the carbon source predictions assessed by the fluxes from the corresponding transporters. iMAT predicted correctly only three out of six single carbon sources (us: all six out of six), and correctly all two-carbon sources (two out of two, us: one correct, for the other, only the primary source was correctly predicted) (details, see Additional file 1: Figures S11-S12). While iMAT gave several correct transporter reaction predictions, mCADRE did not provide any correct carbon source prediction. In summary, our method led to better flux predictions when compared to iMAT, mCADRE, and pFBA.

### LPM-GEM identifies the carbon sources of the validation set but shows limitations when predicting time-lapse fluxes

So far, we showed how we trained and tested our model utilizing gene expression data to predict carbon sources for *B. subtilis* in eight different steady-state conditions. However, in a natural environment, the bacteria may need to switch from one carbon source to another. Particularly, glucose and malate are preferred carbon sources for which such a switch may occur [56, 57]. We applied our approach to a publicly available time-series dataset consisting of two nutritional shifts, i.e., from glucose to glucose plus malate and from malate to malate plus glucose. In these shifts, *B. subtilis* was grown on a single substrate leading to a steady-state-like initial condition. Then, the other substrate was added. Transcription profiles and ^13^C flux data were generated in a time series until the shift was performed (at an endpoint at 90 min) according to the authors of the original study [13]. We applied the model which had been trained on the first dataset. We investigated the predictions of the two major carbon sources for the two initial conditions (before adding the other carbon source—only glucose and only malate at steady state) and for the two endpoint conditions (90 min after adding malate to glucose (glucose to glucose plus malate), and 90 min after adding glucose to malate (malate to malate plus glucose)). For this, as for the study with the eight carbon sources (described above), we computed the z-scores of the transporters across these steady-state conditions and compared our predicted results with the gold standard. All four out of four steady-state conditions were predicted correctly (Fig. 3b, Additional file 1: Figure S7b).

Next, we investigated how our model predicted the time-series of the shifts and again compared the predicted fluxes of the malate and glucose transporters with the fluxes from the gold standard across all time points (Additional file 1: Figure S13 and Table S16). When the carbon source was shifted from malate to malate plus glucose, our predicted fluxes from glucose and malate transporters correlated quite well with the gold standard (r = 0.68 for the glucose transporter, r = 0.48 for the malate transporter). We also expected good prediction results for the shift from glucose to glucose plus malate, as although the order was changed, the conditions were based on the same carbon sources. While the prediction for the malate transporter was very good (r = 0.98 for the shift of glucose to glucose plus malate), the prediction for the glucose transporter was very poor, i.e., the flux prediction was even negatively correlated to the gold standard (r = −0.21). We explain this discrepancy in Discussion. The flux predictions from both shifts are provided in Additional file 1: Tables S17 and S18.

In summary, applying the model trained with the data from the first study (eight carbon sources) to the unknown data from the validation set (glucose/malate carbon source shift), the model correctly identified the carbon sources at baseline and at the endpoints of the carbon source shifts. For the prediction of the time lapse, the model predicted the time-lapse behavior of the main nutrients for the shift from malate to malate plus glucose correctly. However, the model had major difficulties for the shift from glucose to glucose plus malate, and this will be discussed below.

## Discussion

We established a novel method employing gene expression profiles to estimate metabolic fluxes in a systems view. During implementing the method, we observed that the optimization to fit the fluxes to expression profiles utilized thermodynamically infeasible loops (TILs), leading to low prediction performances. We addressed this issue following three different strategies. First, we came up with a novel approach (IFFPR) for reducing the upper and lower bounds by iteratively reducing the flux ranges of each considered reaction. Furthermore, we recognized that using an existing well-established method to reduce TIL (ll-COBRA) was very powerful, but it was quite CPU intensive for our purposes. We addressed this issue and introduced our new method RED-TIL as an alternative method. ll-COBRA formulates one large problem and searches for an optimal solution in a predefined-thermodynamic feasible region. In turn, employing a bottom-up design, RED-TIL splits the overall problem into smaller problems by detecting a TIL in the optimal solution, excluding it from the solution space, and re-optimizing the solution iteratively until no TIL (within a certain limit) is detected. While the results were comparable, the computational speed for RED-TIL was considerably faster. On average, RED-TIL removed TIL from the relevant solution space three times faster than ll-COBRA. Such a speed-up was relevant for our study as we needed to generate solutions for a larger range of different parameter settings, particularly for optimizing the upper and lower bounds for each reaction when running IFFPR. As a third means to reduce TIL, we penalized the sum of fluxes of non-core reactions. All three methods to reduce TIL improved our predictions. Using these methods, we set up an FBA model based on a linear fit between the expression of the encoding genes for an enzyme and its predicted flux. We aimed to predict the main carbon source for the model organism *B. subtilis*. We identified the correct major carbon sources for all eight conditions based on the corresponding gene expression profiles. LPM-GEM was developed to be used as a comparative method to identify the major carbon sources and was tested here for single or two carbon sources. In a typical application, LPM-GEM may be also applied to investigations in rich media conditions. As a future aspect, the performance of LPM-GEM may be tested with data from different rich media conditions by e.g. comparing the predictions with the consumption rates of the cells from the supernatant. Moreover, for most reactions in substrate uptakes, glycolysis, and TCA cycle, the flux prediction results correlated well with ^13^C metabolic flux data. We could very well model the major intracellular changes in carbon metabolism when the carbon sources changed, and particularly the direction of fluxes and the switch between glycolysis (using NAD-dependent GapA) and gluconeogenesis (using NADP-dependent GapB).

We benchmarked our method with the well-known methods (iMAT, mCADRE, and pFBA). Without prior knowledge of carbon sources, pFBA failed to distinguish flux profiles. The method only showed a good flux prediction performance when carbon sources were known. It was then excluded from further comparisons as it did not suit the principal aim of the study (i.e., the prediction of carbon sources). Compared to iMAT and mCADRE, our method yielded better flux predictions and, on average, better predictions of the carbon source. A reason for this may be that our method requires no binarization/discretization of the transcription profiles. iMAT, mCADRE, and other approaches [27, 28, 31–33] need expression level thresholds to decide whether a reaction needs to be active (constrained or part of the optimization to have a non-zero flux) or not. In turn, our method makes use of the continuous nature of gene expression based on a linear regression model to fit the metabolic fluxes.

Using the model trained with the dataset of the eight carbon sources, we validated our approach with a second publicly available dataset in which a shift in the carbon source from glucose to glucose plus malate and from malate to malate plus glucose was investigated [13]. Our model correctly predicted the carbon sources of the initial setting and the endpoints. Also, the model predictions of the shift from malate to malate plus glucose correlated well with the gold standard (^13^C tracer derived flux from the original study). Although gene expression data is scalable and easy to obtain compared to ^13^C metabolic flux data [34–36], it provides only indirect information for the estimation of metabolic fluxes. For some conditions or settings, the metabolic flux may not be controlled by transcription of the corresponding enzyme coding genes. Other flux control mechanisms, e.g., related to limitations due to substrate or product concentrations, translational regulation, covalent modification of the enzymes, or allosteric regulation, can influence the metabolic flux [58]. We observed this limitation in the nutrient shift from glucose to glucose plus malate. Here, we observed very poor prediction results suggesting that this shift might not be controlled by transcriptional regulation. The result from our model is in line with the observations reported in the original study by Buescher et al. [13]. They assumed that these shifts are mediated by fundamentally different control mechanisms. In order to confirm their assumption, Buescher et al. performed a multi-omics analysis of time-lapse profiles from promoter activity, mRNA, and protein abundance to identify post-transcriptional events [13]. After correlating the gene expression levels with the protein levels, they observed high positive correlations in gene-protein pairs related to glycolysis such as phosphoglycerate mutase (r = 0.96), PTS glucose transporter (r = 0.88), and glyceraldehyde 3-phosphate dehydrogenase (r = 0.96) for the shift from malate to malate plus glucose. However, they could not find correlations in gene-protein pairs related to glycolysis in the glucose to glucose plus malate shift. From this, they concluded that the shift from glucose to glucose plus malate was dominantly controlled by post-transcriptional mechanisms (in contrast to the malate to malate plus glucose shift), or proteins for glycolysis are constitutively expressed. The latter is reasonable. As the benefit of glucose consumption is very high compared to malate, it may be beneficial to keep proteins for glycolysis constitutively expressed under the malate condition. This observation serves as a good example of a limitation of our approach. The method relies on gene expression profiles to predict metabolic fluxes. Hence, it requires a basic understanding of the investigated biology beforehand to avoid studying mechanisms that are likely to depend on other regulation mechanisms than transcriptional regulation. The core reactions used in this study are well-known reactions in central energy metabolism and are commonly used as core reactions in metabolic modelling studies. We mainly used core reactions for which ^13^C tracer derived flux data was available. In principle, our method enables using any set of enzymes as core reactions which are of particular interest. Still, also here, the selection needs a sufficient biological background knowledge to circumvent studying unrealistic scenarios which may be due to the fact that the enzymes under study are not regulated on the gene expression level or are not the relevant pacemakers in the studied conditions. Even though LPM-GEM showed a distinctive better flux prediction performance compared to other methods tested (iMAT, mCADRE, and pFBA), a limitation of our approach is that it needs more CPU runtime to build the models. For our set of core reactions, this was not limiting our analyses. However, future studies need to be performed to test if models basing on much larger sets of core reactions can also be built in reasonable time.

## Conclusions

We have introduced a novel computational approach integrating gene expression profiles into a metabolic network supported by new methods to reduce TIL. With this, we could well predict the carbon sources of *B. subtilis*. Our study supports the idea that FBA analysis based on gene expression profiles can serve as an alternative to ^13^C tracer analysis. Since our approach adjusts flux levels continuously to gene transcript levels, it circumvents defining thresholds and discretization of the expression data. Still, one should be aware of the limitations when studying processes that are not regulated by gene transcription.

## Supplementary Information

**Additional file 1**. LPM-GEM supplementary information.

## Data Availability

The datasets analyzed during the current study are publicly available. They can be found in the following locations: 1. The first dataset: gene expression data can be retrieved from the publication of Nicolas et al. (Table S2) [40], and ^13^C metabolic flux data from ^13^C isotope labeling experiments can be accessed from the publication of Chubukov et al. (Table S4) [14]. 2. The second dataset: gene expression data and ^13^C metabolic flux data are available from the publication of Buescher et al. (https://basysbio.ethz.ch/openbis/basysbio_openbis.html) [13]

## Abbreviations

FBA: Flux balance analysis
LPM-GEM: Linear programming based gene expression model
CBM: Constraint-based modeling
*B. subtilis*: *Bacillus subtilis*
FVA: Flux variability analysis
IFFPR: Iterative feasible flux space reduction
TIL: Thermodynamically infeasible loop
RED-TIL: REDucing the number of thermodynamically infeasible loops
iMAT: integrative Metabolic Analysis Tool
mCADRE: metabolic Context-specificity Assessed by Deterministic Reaction Evaluation
pFBA: parsimonious enzyme usage Flux Balance Analysis
