# Supplementary material for: Linear programming based gene expression model (LPM-GEM) predicts the carbon source for Bacillus subtilis
Source: BMC Bioinformatics. 2022 Jun 10;23:226. doi: 10.1186/s12859-022-04742-7 (PMC9188260; doi:10.1186/s12859-022-04742-7)
Supplement: Supplementary file 1 — Additional file 1. LPM-GEM supplementary information. [file 12859_2022_4742_MOESM1_ESM.pdf]

# Linear Programming based Gene Expression Model (LPM-GEM) predicts the carbon source for *Bacillus subtilis*

**Kulwadee Thanamit[1], Franziska Hoerhold[1], Marcus Oswald[1], Rainer Koenig[1]**

[1]Systems Biology Research Group, Institute for Infectious Diseases and Infection Control (IIMK), Jena University Hospital, Kollegiengasse 10, 07743, Jena, Germany

## Supplementary information

## (Figure S1-S13, Table S1-S18)

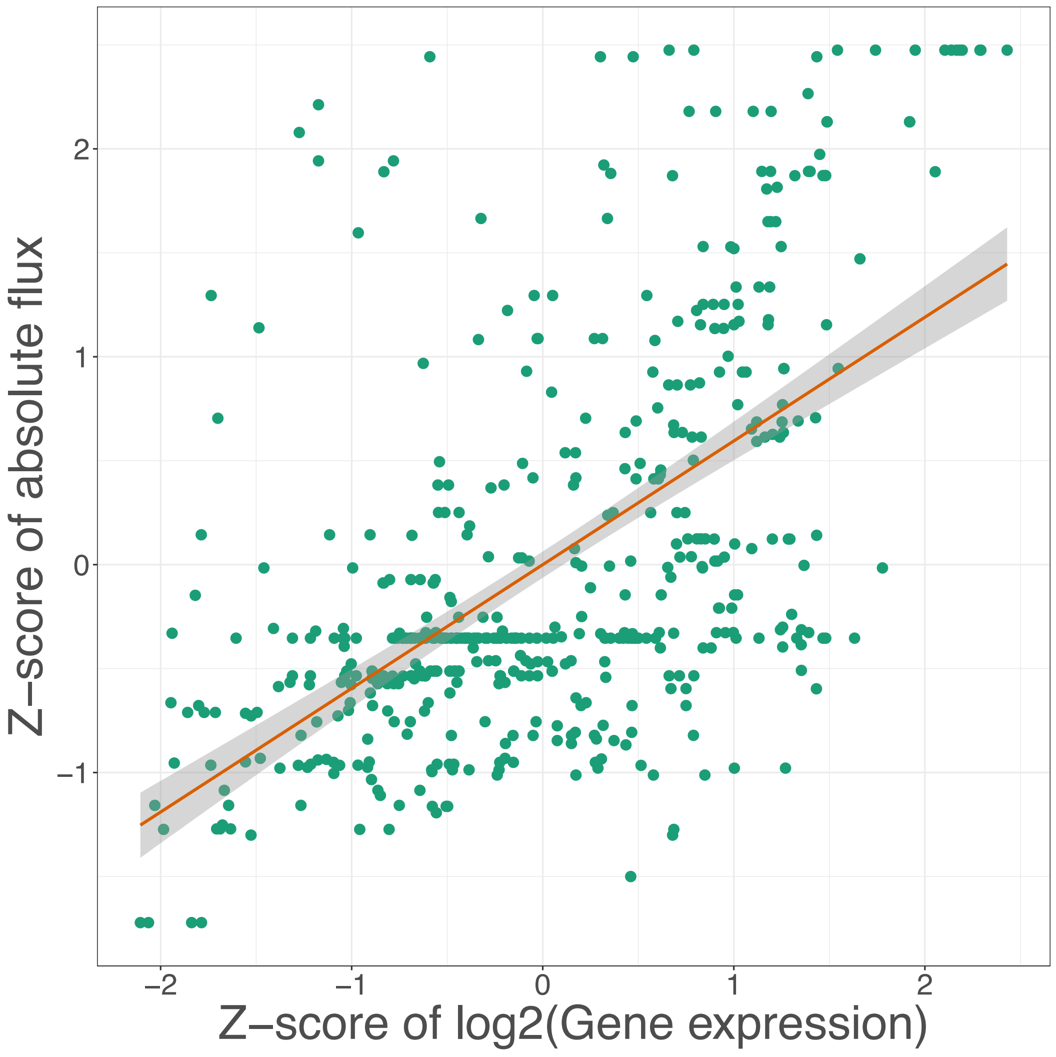


**Figure S1:** Z-scores of the absolute values of the $^{13}C$ tracer derived metabolic flux data from 40 reactions of the gold standard and corresponding gene expression values from the same carbon source conditions, and a linear regression line are plotted. The scatterplot shows a linear tendency, Pearson's correlation coefficient was significant ($r = 0.59$, p-value = $2.13 \times 10^{-53}$).

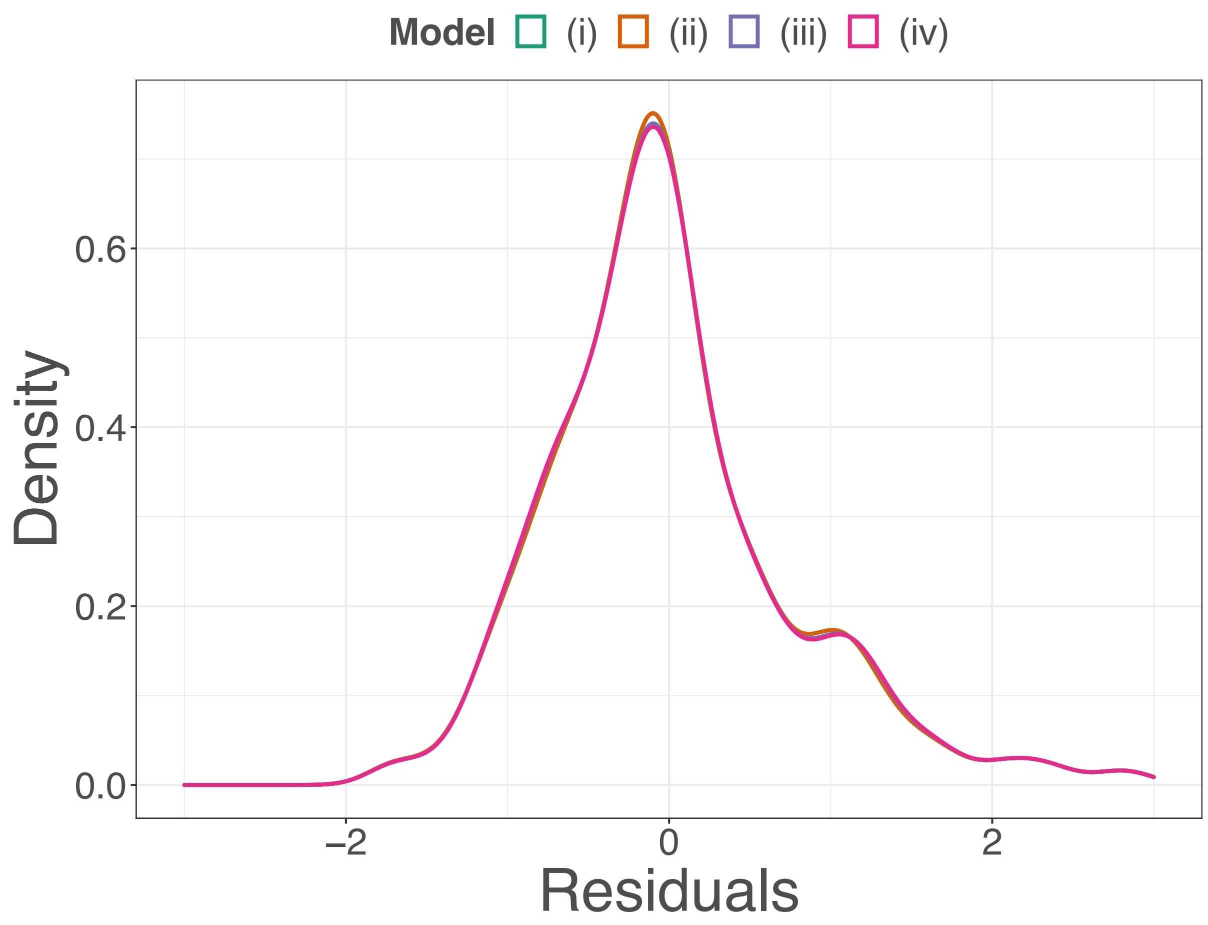


**Figure S2:** Four different transformations of the gene expression data were compared with the $^{13}C$ flux data values. We tested

(i) a log-linear model (the model used in our study, see also Figure S1), i.e.

$$y = z_{|flux|},\ x = z_{\log_2(gene\ expression)},$$

in which $z_{|flux|}$ are the z-transformed absolute values of the fluxes from the $^{13}C$ flux data, and $z_{\log_2(gene\ expression)}$ are the z-transformed values of the log2 transformed values of the gene expression values;

(ii) a linear model,

$$y = z_{\log_2(|flux|+1)},\ x = z_{\log_2(gene\ expression)},$$

in which $z_{\log_2(|flux|+1)}$ are the z-transformed values of the log2 transformed absolute values of the fluxes. A constant of +1 was added to cope for zeros in the logarithm. To note, non-

logarithmic values were not suitable for this investigation as we performed a linear regression (see below) which needs normally distributed data. Next, we tested

(iii) a model using the square root of the expression data, i.e.

$$y = z_{|flux|},\ x = z_{\sqrt{\log_2(gene\ expression)}},$$

in which the y values were derived as described above and the x values were the z-transformed values of the square root of the log2 transformed gene expression values; and finally

(iv) a model using the cubic square root of the expression data, i.e.

$$\mathrm{y} = z_{|flux|},\ x = z_{\sqrt[3]{\log_2(gene\ expression)}},$$

in which the y values were derived as described above and the x values were the z-transformed values of the cubic square root of the log2 transformed gene expression values.

The x and y values were used to calculate a linear regression model. We did not see large differences in the residuals as depicted in the figure, i.e. σ was 0.75, 0.75, 0.76 and 0.76 for models (i) to (iv), respectively. To confirm this by a statistical test, the residuals of the respective regression models were compared performing an F-test, in which the null hypothesis was model (i) and the alternative hypotheses were models (ii), (iii) and (iv), respectively. All tests showed that the residuals were not significantly different.

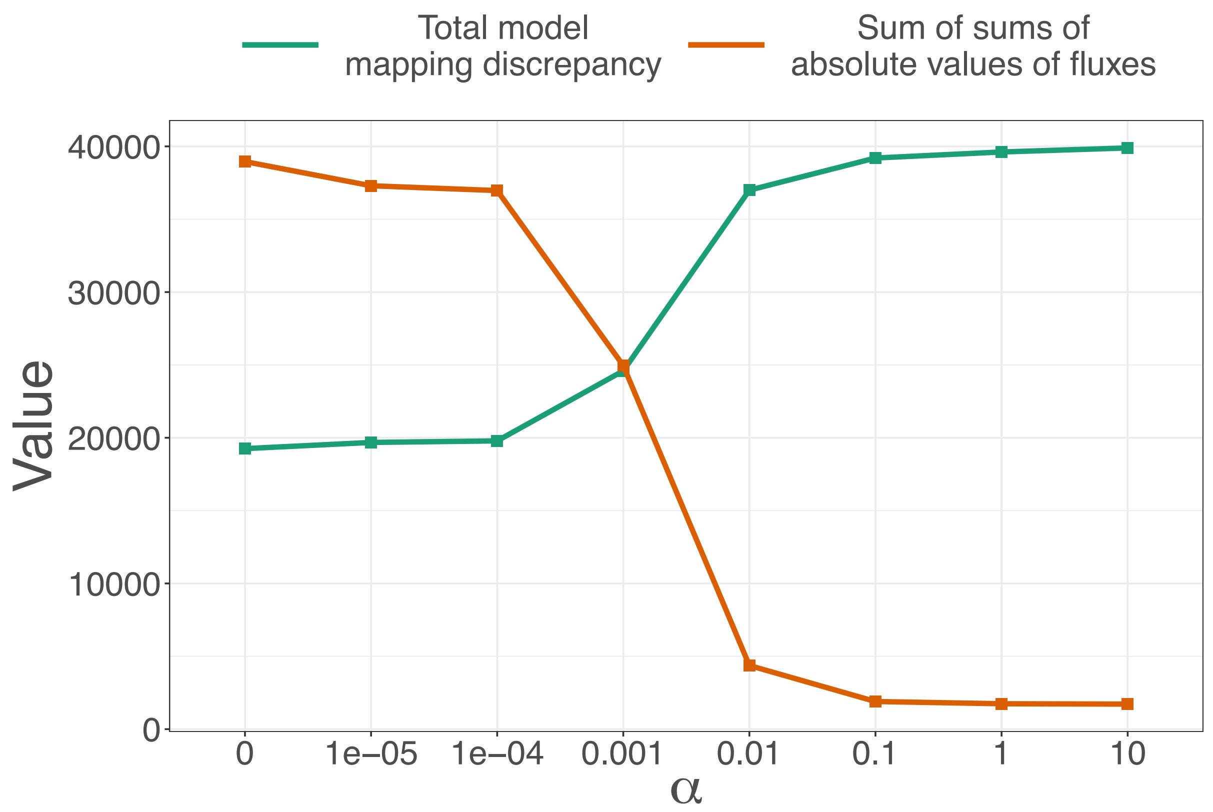


**Figure S3:** Trade-off between the sum of sums of absolute values of fluxes from non-core and non-associated reactions and the total model mapping discrepancy calculated across all eight conditions at different values of parameter $\alpha$ before applying IFFPR and RED-TIL. At $\alpha$ = 0.01, the sum of sums of absolute values of fluxes drops considerably while the total model mapping discrepancy only moderately increases.

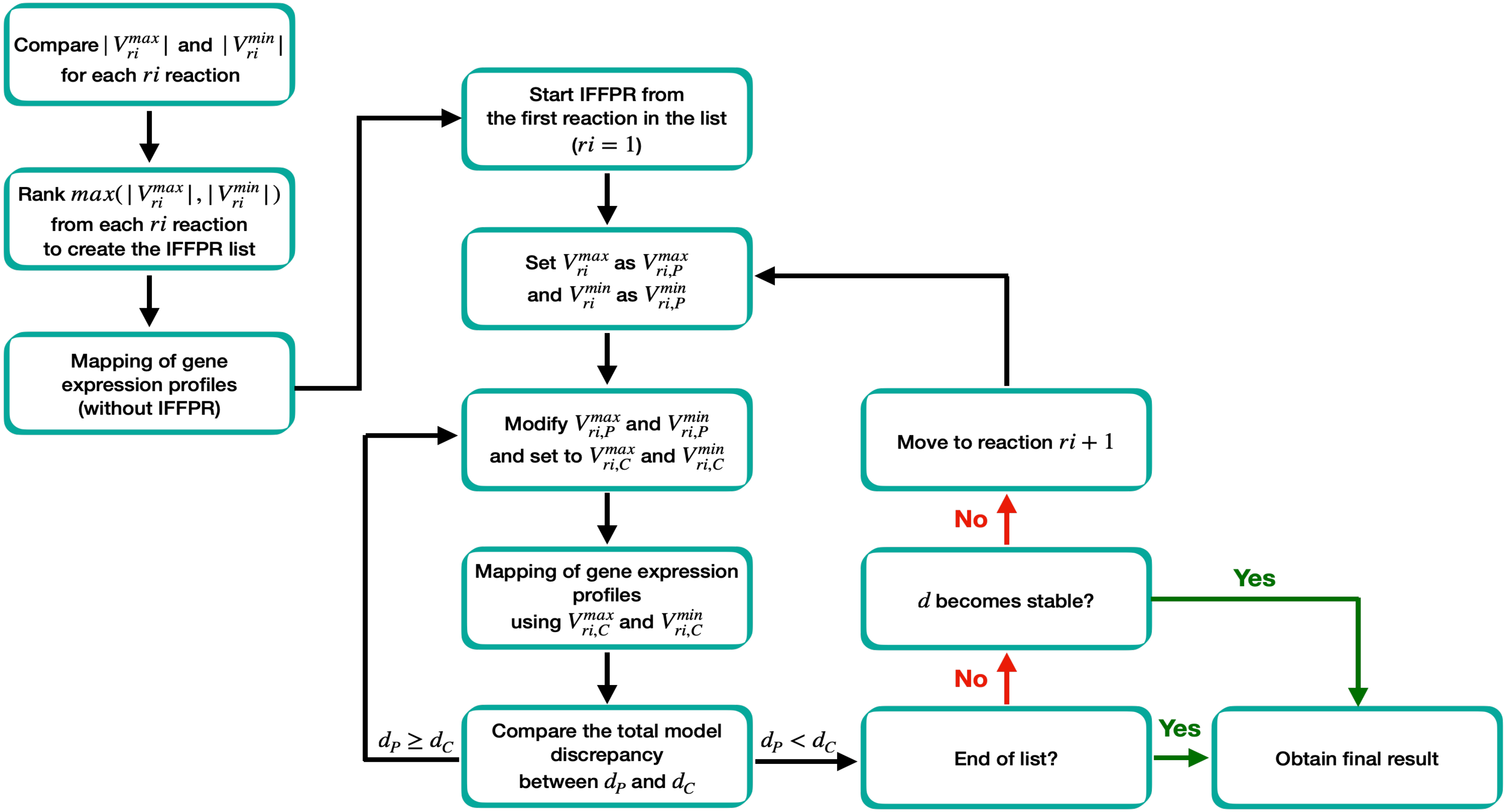


**Figure S4:** The workflow of IFFPR.

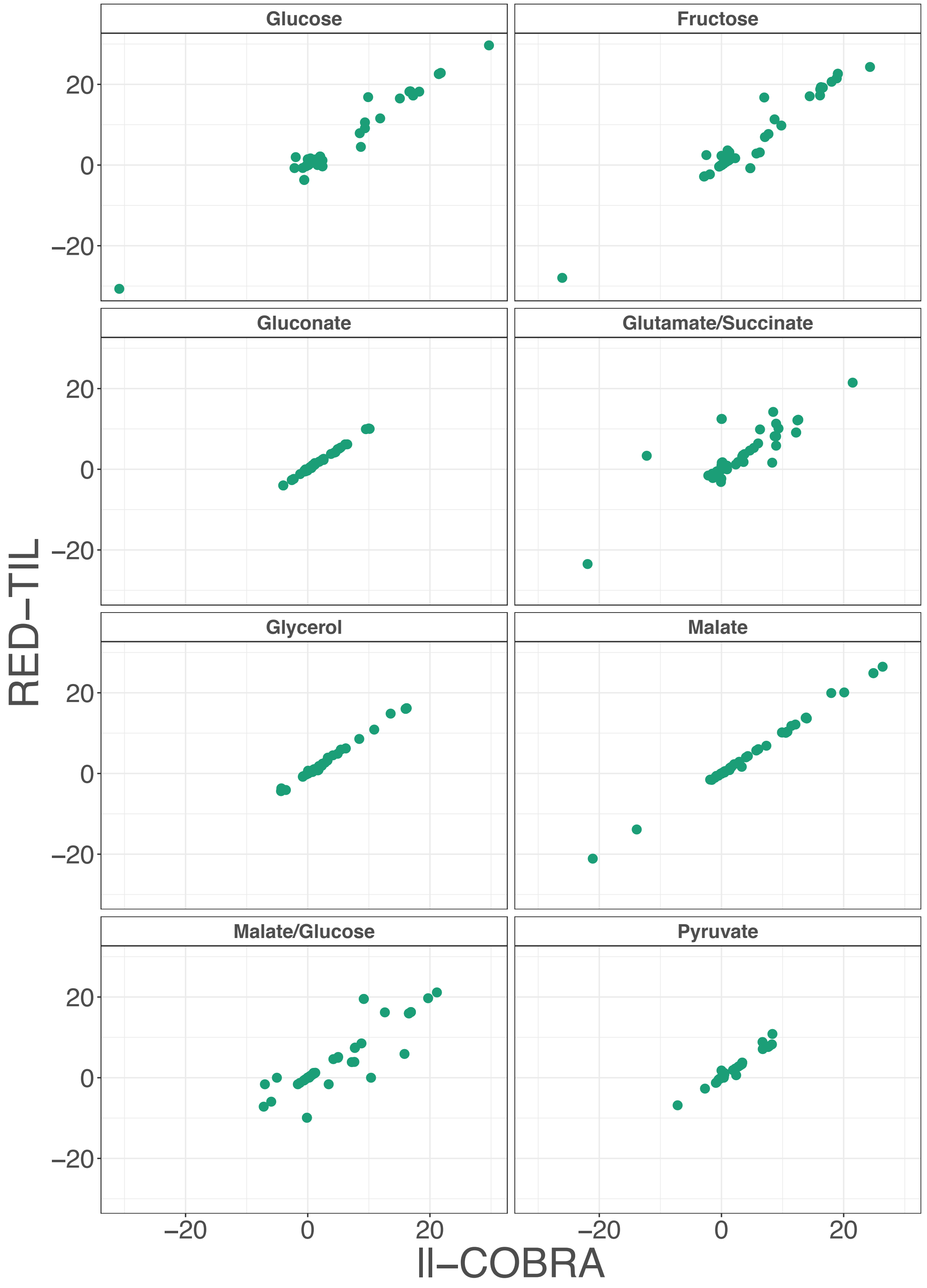


**Figure S5:** Scatterplots show the predicted fluxes from RED-TIL of the core and associated reactions (98 reactions) *versus* the predicted fluxes from ll-COBRA.

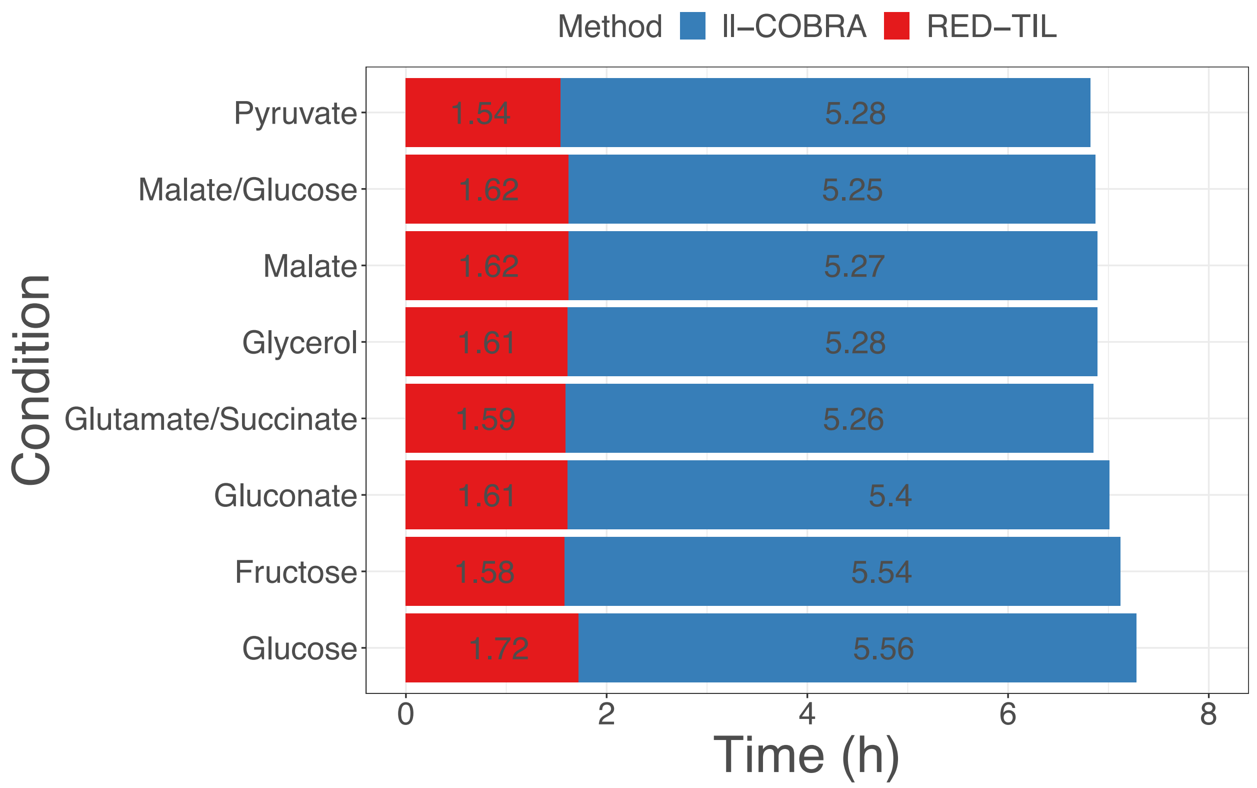


**Figure S6:** Computational running time (hours) of ll-COBRA and RED-TIL for each carbon source condition.

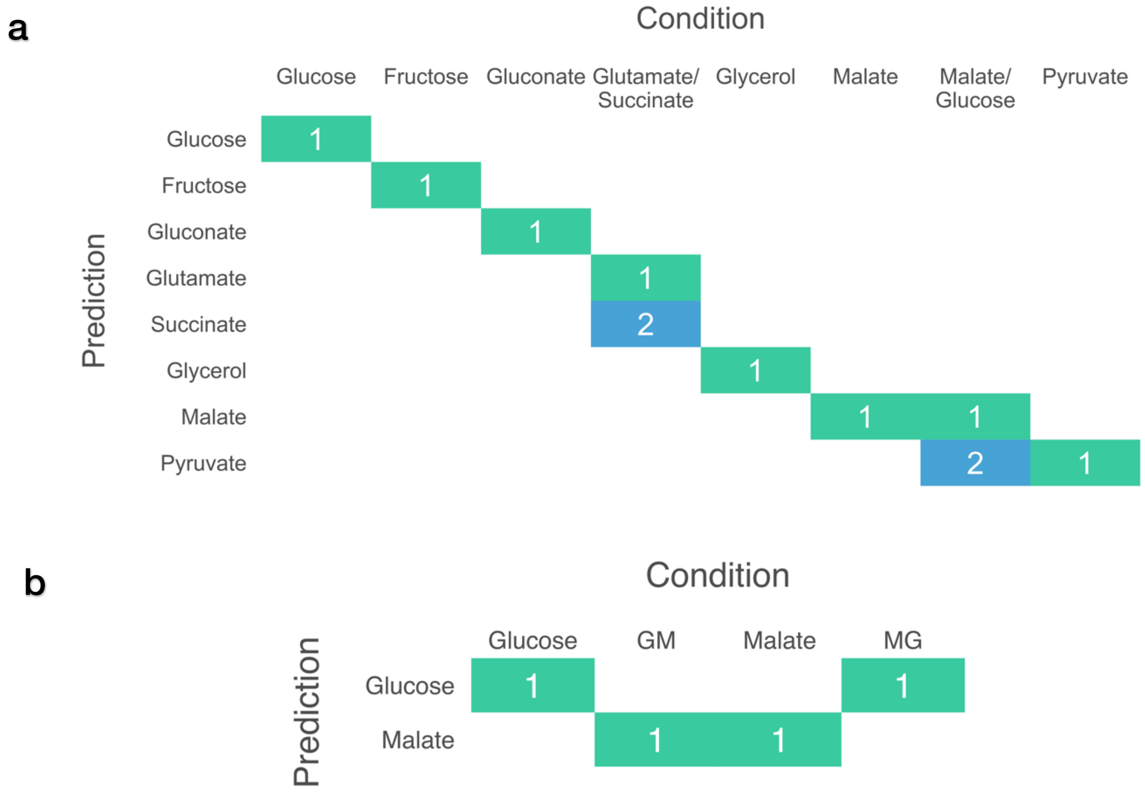


**Figure S7: a** Carbon source predictions of the eight-carbon-sources study. 1: first prediction, 2: second prediction; **b** Prediction of the nutritional shift for the initial and the endpoint conditions, GM: glucose to glucose plus malate shift, 90 min after adding malate; MG: malate to malate plus glucose shift, 90 min after adding glucose.

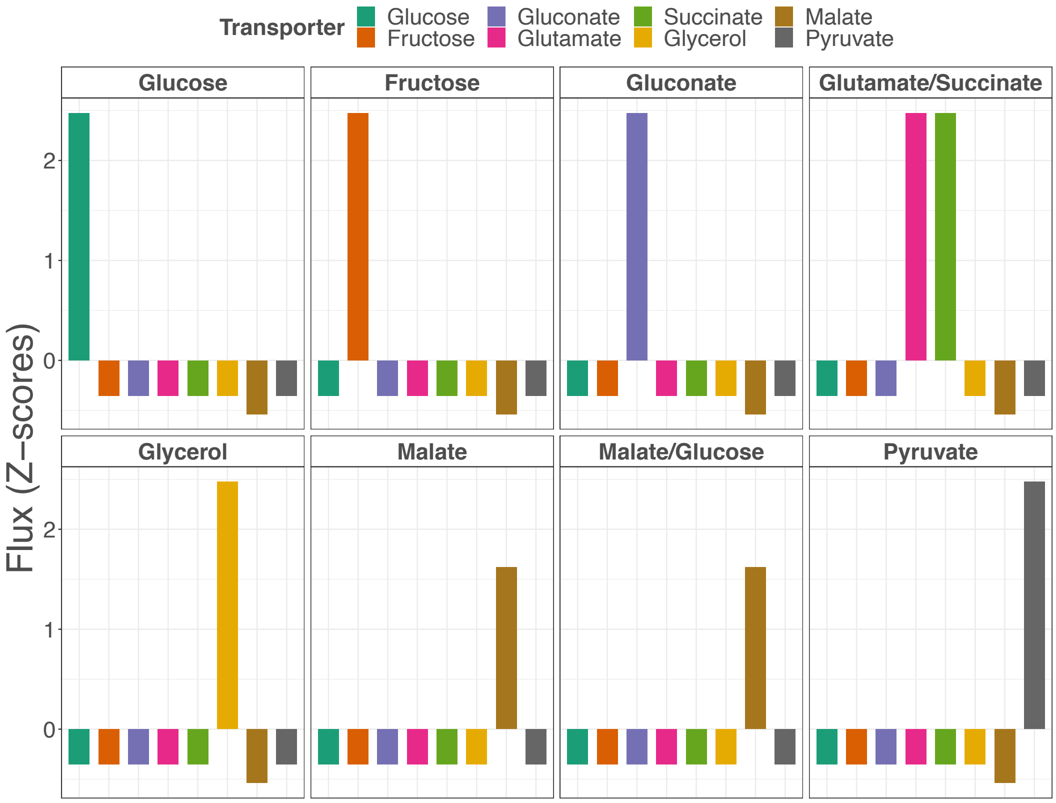


**Figure S8:** Predictions of the carbon source of the first dataset using pFBA. In this scenario, one or two carbon sources were allowed according to each specific condition. A higher z-score indicates a higher probability for a specific carbon source.

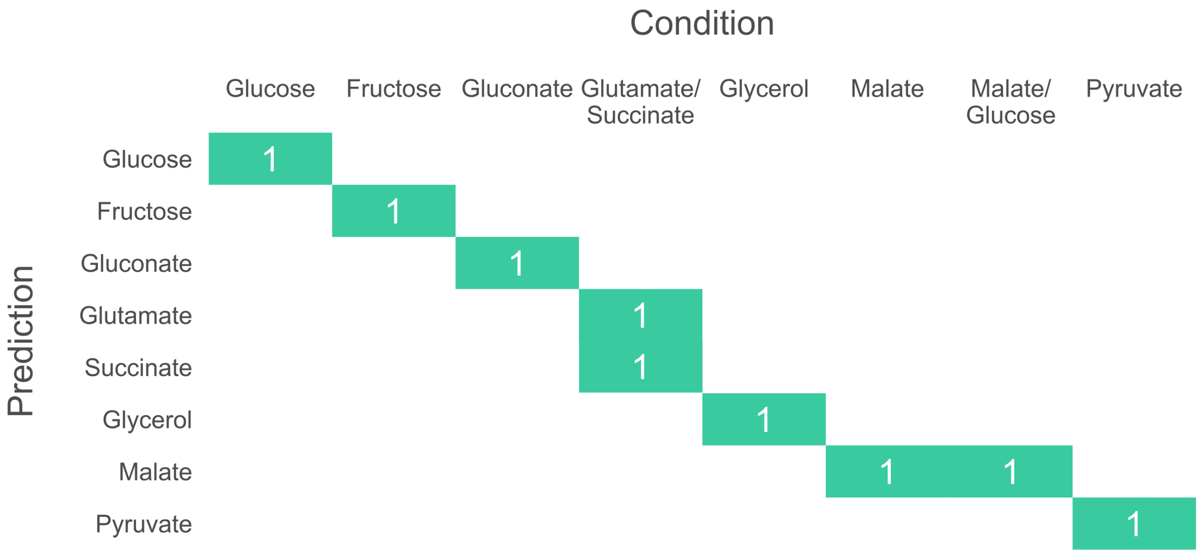


**Figure S9:** Prediction of the carbon source for the first dataset from pFBA (one or two carbon sources allowed according to each specific condition).

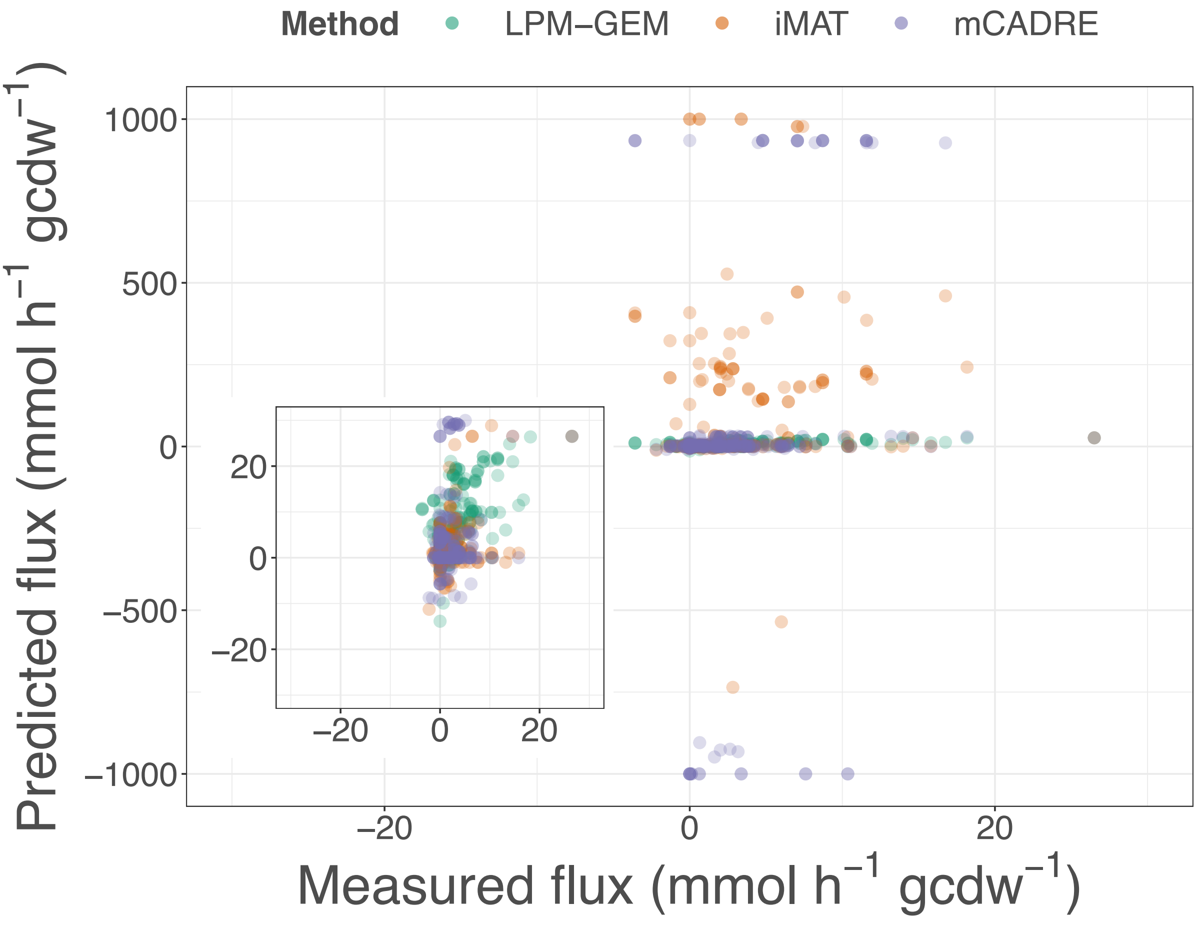


**Figure S10:** Scatterplot of predicted *versus* the measured fluxes (from $^{13}C$ tracer derived metabolic fluxes).

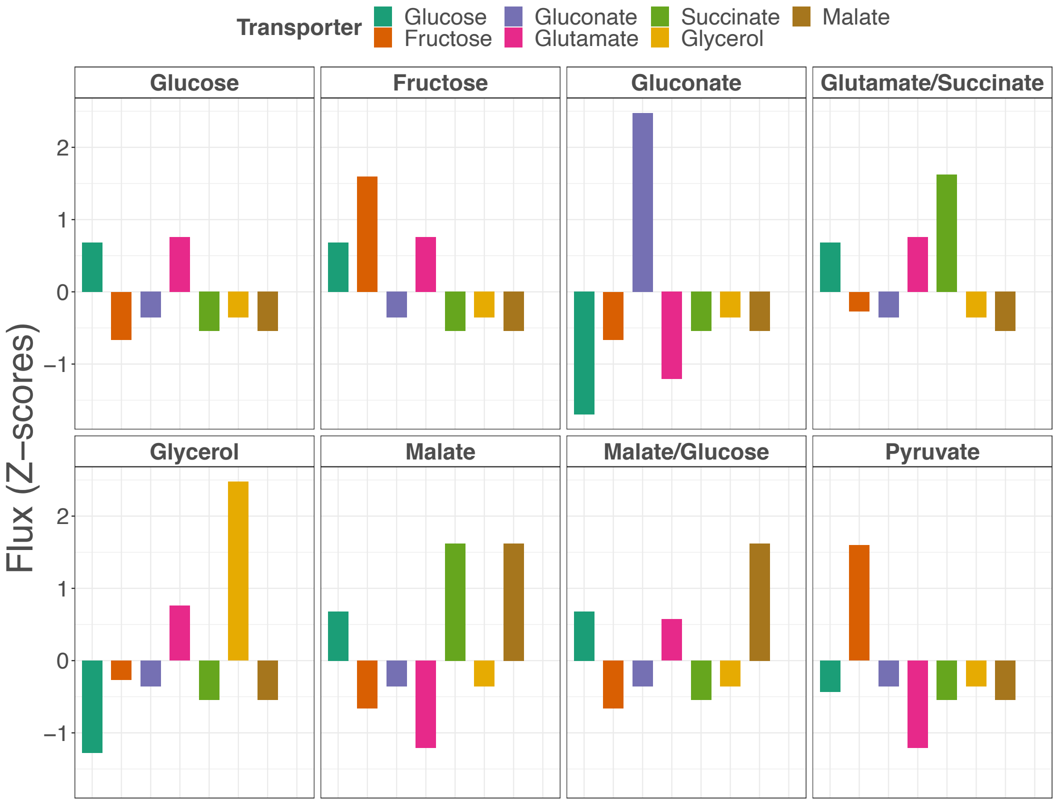


**Figure S11:** Predictions of the carbon source of the first dataset using iMAT. A higher z-score indicates a higher probability for a specific carbon source.

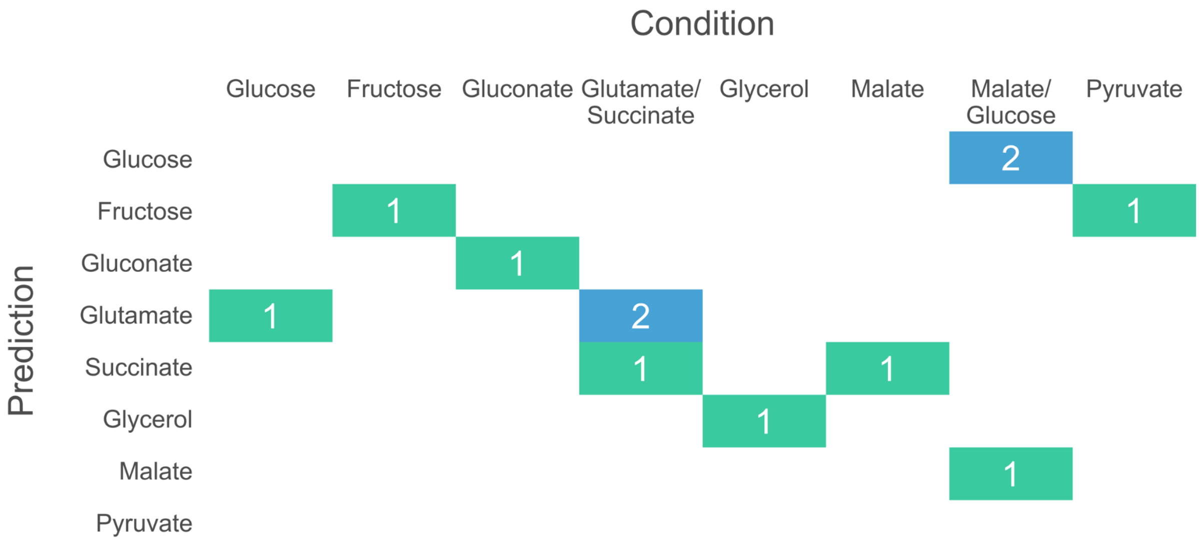


**Figure S12:** Prediction of the carbon source for the first dataset from iMAT.

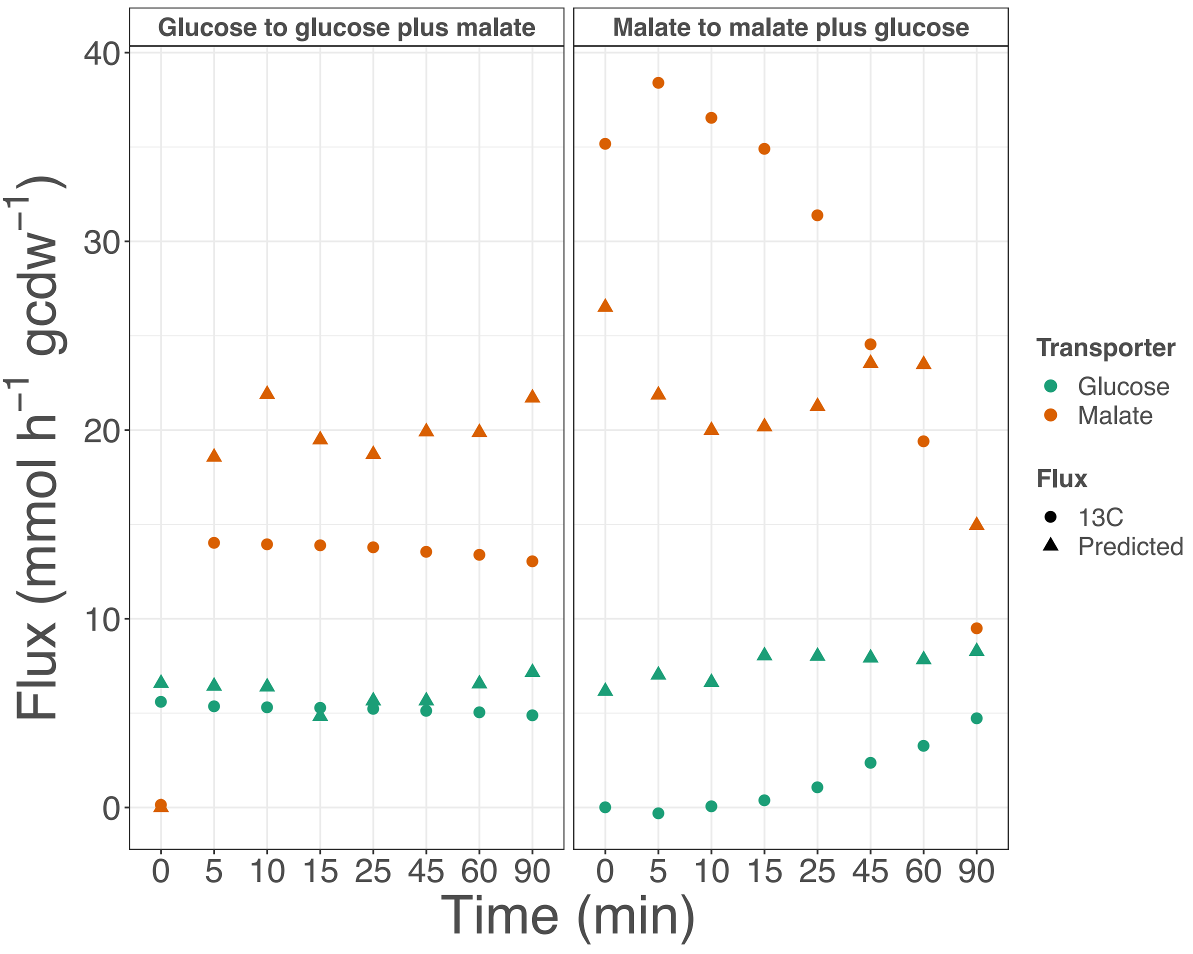


**Figure S13:** Carbon source shifts between glucose and malate. After adding the second substrate, the malate transporter quickly adjusts the uptake rate as shown in both shifts. In contrast, the glucose transporter delays its response.

**Table S1:** List of gene-protein-reaction (GPR) associations from the central energy metabolism of *B. subtilis*. The remaining GPRs after applying P-value = 0.05 cutoff are highlighted in yellow.

| Gene symbol | Gene ID | Flux name |
|---|---|---|
| ackA | BSU29470 | ACKr |
| citA | BSU09440 | CS |
| citB | BSU18000 | ACONT |
| citZ | BSU29140 | CS |
| dctP | BSU04470 | SUCCt2r |
| eno | BSU33900 | ENO |
| fbaA | BSU37120 | FBA |
| fbp | BSU40190 | FBP |
| fruA | BSU14400 | FRUpts |
| fumC | BSU33040 | FUM |
| gapA | BSU33940 | GAPD |
| gapB | BSU29020 | GAPDi_nadp |
| glpF | BSU09280 | GLYCt |
| gltP | BSU02340 | GLUt2r |
| gndA | BSU23860 | GND |
| gntP | BSU40070 | GLCNt2ir |
| gntZ | BSU40080 | GND |
| icd | BSU29130 | ICDHyr |
| levD | BSU27070 | FRUpts |
| levE | BSU27060 | FRUpts |
| levF | BSU27050 | FRUpts |
| levG | BSU27040 | FRUpts |
| ptsH | BSU13900 | FRUpts |
| ptsI | BSU13910 | FRUpts |
| lrgA | BSU28910 | PYRt2 |
| lrgB | BSU28900 | PYRt2 |
| maeA | BSU37050 | ME2 |
| maeN | BSU31580 | MALt4 |
| yflS | BSU07570 | MALt4 |
| maeN | BSU31580 | MALt10 |
| yflS | BSU07570 | MALt10 |
| maeN | BSU31580 | MALt2r |
| yflS | BSU07570 | MALt2r |
| malS | BSU29880 | ME2 |
| mdh | BSU29120 | MDH |
| mleA | BSU23550 | ME2 |
| odhA | BSU19370 | AKGDH |
| odhB | BSU19360 | AKGDH |
| pckA | BSU30560 | PPCK |

| Gene symbol | Gene ID | Flux name |
|---|---|---|
| pdhA | BSU14580 | PDH |
| pdhB | BSU14590 | PDH |
| pdhC | BSU14600 | PDH |
| pdhD | BSU14610 | PDH |
| pfkA | BSU29190 | PFK |
| pgi | BSU31350 | PGI |
| pgk | BSU33930 | PGK_1 |
| pgm | BSU33910 | PGM_1 |
| pps | BSU18830 | PPS |
| pta | BSU37660 | PTAr |
| ptsG | BSU13890 | GLCpts |
| ptsH | BSU13900 | GLCpts |
| ptsI | BSU13910 | GLCpts |
| pycA | BSU14860 | PC |
| pyk | BSU29180 | PYK |
| rpe | BSU15790 | RPE |
| sdhA | BSU28440 | SUCD1 |
| sdhB | BSU28430 | SUCD1 |
| sdhC | BSU28450 | SUCD1 |
| sucC | BSU16090 | SUCOAS |
| sucD | BSU16100 | SUCOAS |
| tkt | BSU17890 | TKT1 |
| tkt | BSU17890 | TKT2 |
| tpiA | BSU33920 | TPI |
| ykgB | BSU13010 | G6PDH2r |
| ytsJ | BSU29220 | ME2 |
| ywjH | BSU37110 | TALA |
| ywlF | BSU36920 | RPI |
| zwf | BSU23850 | G6PDH2r |
| fruK | BSU14390 | FRUK |
| glpK | BSU09290 | GLYK |
| serA | BSU23070 | PGCD |
| serC | BSU10020 | PSERT |
| yoaD | BSU18560 | PGCD |
| rsbX | BSU04740 | PSP_L |
| sdaAB | BSU15850 | SERD_L |
| sdaAA | BSU15860 | SERD_L |
| fbaA | BSU37120 | FBA2 |
| gntK | BSU40060 | GNKr |
| glxK | BSU40040 | GLYCK |
| nadB | BSU27870 | ASPO1 |
| aspB | BSU22370 | ASPTA |
| mtnE | BSU13580 | UNK5 |
| citM | BSU07610 | CITt10 |
| citM | BSU07610 | CITt11 |
| citM | BSU07610 | CITt12 |

| Gene symbol | Gene ID | Flux name |
|---|---|---|
| citM | BSU07610 | CITt13 |
| citM | BSU07610 | CITt14 |
| citH | BSU39060 | CITt14 |
| citM | BSU07610 | CITt15 |
| cimH | BSU38770 | CITt2r |
| yraO | BSU26860 | CITt2r |
| yfiY | BSU08440 | FEDCabc |
| yfhA | BSU08460 | FEDCabc |
| yfiZ | BSU08450 | FEDCabc |
| citM | BSU07610 | ICITt10 |
| citM | BSU07610 | ICITt2 |
| metA | BSU21910 | HSST |
| scoA | BSU38990 | OCOAT1 |
| scoB | BSU38980 | OCOAT1 |
| prpB | BSU24120 | MCITL2 |
| yodQ | BSU19710 | SDPDS |
| metI | BSU11870 | SHSL1_1 |
| metI | BSU11870 | SHSL2 |
| metI | BSU11870 | SHSL3 |
| metI | BSU11870 | SHSL4r |
| gabD | BSU03910 | SSALy |
| purB | BSU06440 | ADSL1r |
| purB | BSU06440 | ADSL2r |
| argH | BSU29440 | ARGSL |
| ansB | BSU23570 | ASPT |
| yflS | BSU07570 | FUMt2r |
| dctP | BSU04470 | FUMt2r |
| menD | BSU30820 | 2S6HCCi |
| gabT | BSU03900 | ABTA |
| argD | BSU11220 | ACOTA |
| yflS | BSU07570 | AKGt2r |
| yoaB | BSU18540 | AKGt2r |
| yugH | BSU31400 | ALATA_L |
| patA | BSU14000 | APTA1i |
| gudB | BSU22960 | GLUDxi |
| rocG | BSU37790 | GLUDxi |
| gltA | BSU18450 | GLUSy |
| gltB | BSU18440 | GLUSy |
| hisH | BSU34890 | HSTPTr |
| hisC | BSU22620 | HSTPTr |
| rocD | BSU40340 | ORNTA_1 |
| menD | BSU30820 | OXGDC |
| hisC | BSU22620 | PHETA1 |
| hisH | BSU34890 | PHETA1 |
| hisC | BSU22620 | TYRTA |
| hisH | BSU34890 | TYRTA |

| Gene symbol | Gene ID | Flux name |
|---|---|---|
| ywiE | BSU37240 | CLPNS2_BS |
| clsB | BSU37190 | CLPNS2_BS |
| clsA | BSU36590 | CLPNS2_BS |
| yhdN | BSU09530 | ALCD19y |
| iolS | BSU39780 | ALCD19y |
| yhdN | BSU09530 | ALCD19_L |
| iolS | BSU39780 | ALCD19_L |
| glpQ | BSU02130 | GPDDA4 |
| bacD | BSU37710 | LAAL24 |
| bacD | BSU37710 | LAAL25 |
| bacD | BSU37710 | LAAL27 |
| bacD | BSU37710 | LAAL26 |
| bacD | BSU37710 | LAAL28 |
| speA | BSU14630 | ARGDC |
| argI | BSU40320 | ARGN_1 |
| artP | BSU23980 | ARGabc |
| artQ | BSU23970 | ARGabc |
| artR | BSU23960 | ARGabc |
| lysP | BSU33330 | ARGt2r |
| rocE | BSU40330 | ARGt2r |
| rocC | BSU37760 | ARGt2r |
| aldX | BSU39860 | LCADi |
| aldY | BSU38830 | LCADi |
| dhaS | BSU19310 | LCADi |
| ldh | BSU03050 | LDH_L |
| lctP | BSU03060 | L_LACt2r |
| yvfH | BSU34190 | L_LACt2r |
| bacD | BSU37710 | LAAL33 |
| bacD | BSU37710 | LAAL34 |
| trpB | BSU22640 | TRPS1 |
| trpA | BSU22630 | TRPS1 |
| trpP | BSU10010 | TRPt2r |
| cysK | BSU00730 | AHSERL4 |
| cysK | BSU00730 | CYSS_2 |
| mccB | BSU27250 | CYSTGL_1 |
| bacD | BSU37710 | LAAL9 |
| coaBC | BSU15700 | PPNCL |
| mccB | BSU27250 | TRPAS1 |
| hutH | BSU39350 | HISDr |
| hisD | BSU34910 | HISTD |
| hutM | BSU39390 | HISt2r |
| ybgF | BSU02400 | HISt2r |
| bacD | BSU37710 | LAAL40 |
| bacD | BSU37710 | LAAL41 |
| bacD | BSU37710 | LAAL42 |
| bacD | BSU37710 | LAAL10 |

| Gene symbol | Gene ID | Flux name |
|---|---|---|
| bacD | BSU37710 | LAAL16 |
| bacD | BSU37710 | LAAL22 |
| bacD | BSU37710 | LAAL4 |
| tdh | BSU16990 | THRD |
| ilvA | BSU21770 | THRD_L |
| thrC | BSU32250 | THRS |
| pssA | BSU02270 | CDPDSP_BS |
| mccA | BSU27260 | CYSTS_2 |
| glyA | BSU36900 | GHMT2r |
| bacD | BSU37710 | LAAL12 |
| bacD | BSU37710 | LAAL14 |
| bacD | BSU37710 | LAAL18 |
| bacD | BSU37710 | LAAL21 |
| bacD | BSU37710 | LAAL3 |
| bacD | BSU37710 | LAAL31 |
| bacD | BSU37710 | LAAL37 |
| bacD | BSU37710 | LAAL6 |
| bacD | BSU37710 | LAAL7 |
| bacD | BSU37710 | LAAL8 |
| cysE | BSU00930 | SERAT |
| aldX | BSU39860 | ALDD31_1 |
| aldY | BSU38830 | ALDD31_1 |
| dhaS | BSU19310 | ALDD31_1 |
| kbl | BSU17000 | GLYAT |
| thiO | BSU11670 | GLYO1 |
| bacD | BSU37710 | LAAL1 |
| bacD | BSU37710 | LAAL13 |
| bacD | BSU37710 | LAAL19 |
| bacD | BSU37710 | LAAL2 |
| bacD | BSU37710 | LAAL29 |
| bacD | BSU37710 | LAAL35 |
| purD | BSU06530 | PRAGSr |
| thiO | BSU11670 | SARCOX |

**Table S2:** List of other exchange reactions in the metabolic model of *B. subtilis* (excluding carbon sources used in this study). Common exchange reactions used by the model are highlighted in yellow.

| Reaction | Glucose | Fructose | Gluconate | Glutamate/ Succinate | Glycerol | Malate | Malate/ Glucose | Pyruvate |
|---|---|---|---|---|---|---|---|---|
| EX_2ddglcn(e) | 0 | 0 | 0 | 0 | 0 | 0 | 0 | 0 |
| EX_2hxmp(e) | 0 | 0 | 0 | 0 | 0 | 0 | 0 | 0 |
| EX_2pg(e) | 0 | 0 | 0 | 0 | 0 | 0 | 0 | 0 |
| EX_2pglyc(e) | 0 | 0 | 0 | 0 | 0 | 0 | 0 | 0 |
| EX_3amba(e) | 0 | 0 | 0 | 0 | 0 | 0 | 0 | 0 |
| EX_3amp(e) | 0 | 0 | 0 | 0 | 0 | 0 | 0 | 0 |
| EX_3cmp(e) | 0 | 0 | 0 | 0 | 0 | 0 | 0 | 0 |
| EX_3gmp(e) | 0 | 0 | 0 | 0 | 0 | 0 | 0 | 0 |
| EX_3pg(e) | -0.20875 | 0 | -0.1486 | 0 | 0 | 0 | 0 | 0 |
| EX_3ump(e) | 0 | 0 | 0 | 0 | 0 | 0 | 0 | 0 |
| EX_4abut(e) | 0 | 0 | 0 | 0 | 0 | 0 | 0 | 0 |
| EX_5mtr(e) | 0 | 0 | 0 | 0 | 0 | 0 | 0 | 0 |
| EX_6pgc(e) | 0 | 0 | 0 | 0 | 0 | 0 | 0 | 0 |
| EX_Larab(e) | 0 | 0 | 0 | 0 | 0 | 0 | 0 | 0 |
| EX_Lcyst(e) | 0 | 0 | 0 | 0 | 0 | 0 | 0 | 0 |
| EX_abt__L(e) | 0 | 0 | 0 | 0 | 0 | 0 | 0 | 0 |
| EX_ac(e) | 3.43207 | 0.68196 | 0.04276 | 0.03494 | 0.28084 | 10.60145 | 7.71411 | 0.05534 |
| EX_acac(e) | 0.67875 | 0.59655 | 0.5033 | 0.21154 | 0.03756 | 0.46672 | 0.5999 | 1.01945 |
| EX_acgam(e) | 0 | 0 | 0 | 0 | 0 | 0 | 0 | 0 |
| EX_acmana(e) | 0 | 0 | 0 | 0 | 0 | 0 | 0 | 0 |
| EX_acnam(e) | -0.16737 | -0.12965 | -0.00676 | 0 | 0 | 0 | -0.24523 | 0 |
| EX_actn__R(e) | 0 | 0 | 0 | 0 | 0 | 0 | 0 | 0 |

| Reaction | Glucose | Fructose | Gluconate | Glutamate/ Succinate | Glycerol | Malate | Malate/ Glucose | Pyruvate |
|---|---|---|---|---|---|---|---|---|
| EX_ade(e) | 0 | 0 | 0 | 0 | 0 | 0 | 0 | 0 |
| EX_adn(e) | 0 | 0 | 0 | 0 | 0 | 0 | 0 | 0 |
| EX_akg(e) | 0 | 0 | 0 | 0 | 0 | 0 | 0 | 0 |
| EX_ala_B(e) | 0 | 0 | 0 | 0 | 0 | 0 | 0 | 0 |
| EX_ala__D(e) | 0 | 0 | 0 | 0 | 0 | 0 | 0 | 0 |
| EX_ala_L_Thr__L(e) | 0 | 0 | 0 | 0 | 0 | 0 | 0 | 0 |
| EX_ala_L_asp__L(e) | 0 | 0 | 0 | 0 | 0 | 0 | 0 | 0 |
| EX_ala__L(e) | 0 | 0 | 0 | 0 | 0 | 0 | 0 | 0 |
| EX_ala_L_gln__L(e) | 0 | 0 | 0 | 0 | 0 | 0 | 0 | 0 |
| EX_ala_L_glu__L(e) | 0 | 0 | 0 | 0 | 0 | 0 | 0 | 0 |
| EX_L_alagly(e) | 0 | 0 | 0 | 0 | 0 | 0 | 0 | 0 |
| EX_ala_L_his__L(e) | 0 | 0 | 0 | 0 | 0 | 0 | 0 | 0 |
| EX_ala_L_leu__L(e) | 0 | 0 | 0 | 0 | 0 | 0 | 0 | 0 |
| EX_alaala(e) | -0.21887 | -0.41258 | -0.2427 | 0 | 0 | 0 | -1.91166 | -0.0613 |
| EX_alltn(e) | 0 | 0 | 0 | 0 | 0 | 0 | 0 | 0 |
| EX_amp(e) | -0.10784 | -1.11902 | -0.07677 | -0.04021 | -0.07311 | -0.21309 | -0.13708 | -0.03107 |
| EX_amylase(e) | 0 | 0 | 0 | 0 | 0 | 0 | 0 | 0 |
| EX_antim(e) | 0 | 0 | 0 | 0 | 0 | 0 | 0 | 0 |
| EX_arab__D(e) | 0 | 0 | 0 | 0 | 0 | 0 | 0 | 0 |
| EX_arab__L(e) | 0 | 0 | 0 | 0 | 0 | 0 | 0 | 0 |
| EX_arbt(e) | 0 | 0 | 0 | 0 | 0 | 0 | 0 | 0 |
| EX_arg__L(e) | 0 | 0 | 0 | 0 | 0 | 0 | 0 | 0 |
| EX_argp(e) | 0 | 0 | 0 | 0.06133 | -0.00673 | 0 | 0.15793 | 0.0458 |
| EX_arsenb(e) | 0 | 0 | 0 | 0 | 0 | 0 | 0 | 0 |
| EX_arsna(e) | 0 | 0 | 0 | 0 | 0 | 0 | 0 | 0 |
| EX_arsni2(e) | 0 | 0 | 0 | 0 | 0 | 0 | 0 | 0 |
| EX_asn__L(e) | 0 | 0 | 0 | 0 | 0 | 0 | 0 | 0 |
| EX_asp__L(e) | 0 | 0 | 0 | 0 | 0 | 0 | 0 | 0 |

| Reaction | Glucose | Fructose | Gluconate | Glutamate/ Succinate | Glycerol | Malate | Malate/ Glucose | Pyruvate |
|---|---|---|---|---|---|---|---|---|
| EX_bilea(e) | 0 | 0 | 0 | 0 | 0 | 0 | 0 | 0 |
| EX_btd_RR(e) | 0 | 0 | 0 | 0 | 0 | 0 | 0 | 0 |
| EX_buts(e) | 0 | 0 | 0 | 0 | 0 | 0 | 0 | 0 |
| EX_cbl2(e) | 0 | 0 | 0 | 0 | 0 | 0 | 0 | 0 |
| EX_cd2(e) | 0 | 0 | 0 | 0 | 0 | 0 | 0 | 0 |
| EX_cellb(e) | 0 | 0 | 0 | 0 | 0 | 0 | 0 | 0 |
| EX_cgly(e) | 0 | 0 | 0 | 0 | 0 | 0 | 0 | 0 |
| EX_chitob(e) | 0 | 0 | 0 | 0 | 0 | 0 | 0 | 0 |
| EX_chol(e) | 0 | 0 | 0 | 0 | 0 | 0 | 0 | 0 |
| EX_chols(e) | 0 | 0 | 0 | 0 | 0 | 0 | 0 | 0 |
| EX_chor(e) | 0 | 0 | 0 | 0 | 0 | 0 | 0 | 0 |
| EX_cit(e) | 0 | 0 | 0 | 0 | 0 | 0 | 0 | 0 |
| EX_citr__L(e) | 0 | 0 | 0 | 0 | 0 | 0 | 0 | 0 |
| EX_cmp(e) | 0 | 0 | 0 | 0 | 0 | 0 | 0 | 0 |
| EX_cobalt2(e) | 0 | 0 | 0 | 0 | 0 | 0 | 0 | 0 |
| EX_crn(e) | 0 | 0 | 0 | 0 | 0 | 0 | 0 | 0 |
| EX_cro4(e) | 0 | 0 | 0 | 0 | 0 | 0 | 0 | 0 |
| EX_csn(e) | 0 | 0 | 0 | 0 | 0 | 0 | 0 | 0 |
| EX_ctbt(e) | 0 | 0 | 0 | 0 | 0 | 0 | 0 | 0 |
| EX_cu2(e) | 0 | 0 | 0 | 0 | 0 | 0 | 0 | 0 |
| EX_cys__D(e) | 0 | 0 | 0 | 0 | 0 | 0 | 0 | 0 |
| EX_cys__L(e) | -0.03362 | -0.0302 | -0.02394 | 0 | 0 | 0 | 0 | 0 |
| EX_cyst__L(e) | 0 | 0 | 0 | 0 | 0 | 0 | 0 | 0 |
| EX_cytd(e) | 0 | 0 | 0 | 0 | 0 | 0 | 0 | 0 |
| EX_dad_2(e) | 0 | 0 | 0 | 0 | 0 | 0 | 0 | 0 |
| EX_dcyt(e) | 0 | 0 | 0 | 0 | 0 | 0 | 0 | 0 |
| EX_dextrin(e) | 0 | 0 | 0 | 0 | 0 | 0 | 0 | 0 |
| EX_dha(e) | 0 | 0 | 0 | 0 | 0 | 0 | 0 | 0 |

| Reaction | Glucose | Fructose | Gluconate | Glutamate/ Succinate | Glycerol | Malate | Malate/ Glucose | Pyruvate |
|---|---|---|---|---|---|---|---|---|
| EX_diact(e) | 0 | 0 | 0 | 0 | 0 | 0 | 0 | 0 |
| EX_djenk(e) | 0 | 0 | 0 | 0 | 0 | 0 | 0 | 0 |
| EX_drib(e) | 0 | 0 | 0 | 0 | 0 | 0 | 0 | 0 |
| EX_dtmp(e) | 1.11111 | 0.98512 | 0.82796 | 0.42767 | 0.53194 | 1.31045 | 1.72614 | 0.27993 |
| EX_ectoine(e) | 0 | 0 | 0 | 0 | 0 | 0 | 0 | 0 |
| EX_etha(e) | 0 | 0 | 0 | 0 | 0 | 0 | 0 | 0 |
| EX_eths(e) | 0 | 0 | 0 | -0.03747 | 0 | 0 | 0 | 0 |
| EX_etoh(e) | 0 | 0 | 0 | 0 | 0 | 0 | 0 | 0 |
| EX_fe2(e) | 0 | 0 | 0 | 0 | 0 | 0 | 0 | 0 |
| EX_ferrich(e) | 0 | 0 | 0 | 0 | 0 | 0 | 0 | 0 |
| EX_ferxa(e) | 0 | 0 | 0 | 0 | 0 | 0 | 0 | 0 |
| EX_fol(e) | 0.13867 | 0.10828 | 0.09973 | 0.04177 | 0 | 0.11918 | 0.19745 | 0.02314 |
| EX_for(e) | 0 | 0 | 0 | 0.04185 | 0 | 0 | 0 | 0 |
| EX_fum(e) | 0.11388 | 0.1013 | 0.08107 | 0.10379 | 0.07048 | 0.25705 | 0.30269 | 0.07861 |
| EX_g1p(e) | 0 | 0 | 0 | 0 | 0 | 0 | 0 | 0 |
| EX_g6p(e) | 0 | 0 | 0 | 0 | 0 | 0 | 0 | 0 |
| EX_gal(e) | 0 | 0 | 0 | 0 | 0 | 0 | 0 | 0 |
| EX_galctr__D(e) | 0 | 0 | 0 | -0.21642 | 0 | 0 | 0 | -0.1611 |
| EX_galt(e) | 0 | 0 | 0 | 0 | 0 | 0 | 0 | 0 |
| EX_galur(e) | 0 | 0 | 0 | 0 | 0 | 0 | 0 | 0 |
| EX_gam6p(e) | 0 | 0 | 0 | 0 | 0 | 0 | 0 | 0 |
| EX_gam(e) | 0 | 0 | 0 | 0 | 0 | 0 | 0 | 0 |
| EX_gbbtn(e) | 0 | 0 | 0 | 0 | 0 | 0 | 0 | 0 |
| EX_glcr(e) | 0 | 0 | 0 | 0 | 0 | 0 | 0 | 0 |
| EX_glcur(e) | 0 | 0 | 0 | 0 | 0 | 0 | 0 | 0 |
| EX_gln__L(e) | 0 | 0 | 0 | 0 | 0 | 0 | 0 | 0 |
| EX_glu__D(e) | 0 | 0 | 0 | 0 | 0 | 0 | 0 | 0 |
| EX_glx(e) | 0 | 0 | 0 | 0 | 0 | 0 | 0 | 0 |

| Reaction | Glucose | Fructose | Gluconate | Glutamate/ Succinate | Glycerol | Malate | Malate/ Glucose | Pyruvate |
|---|---|---|---|---|---|---|---|---|
| EX_gly_asn__L(e) | 0 | 0 | 0 | 0 | 0 | 0 | 0 | 0 |
| EX_gly_asp__L(e) | 0 | 0 | 0 | 0 | 0 | 0 | 0 | 0 |
| EX_gly(e) | 0 | 0 | 0 | 0 | 0 | 0 | 0 | 0 |
| EX_gly_gln__L(e) | 0 | 0 | 0 | 0 | 0 | 0 | 0 | 0 |
| EX_gly_glu__L(e) | 0 | 0 | 0 | 0 | 0 | 0 | 0 | 0 |
| EX_gly_met__L(e) | 0 | 0 | 0 | 0 | 0 | 0 | 0 | 0 |
| EX_gly_pro__L(e) | 0 | 0 | 0 | 0 | 0 | 0 | 0 | 0 |
| EX_glyb(e) | 0 | 0 | 0 | 0 | 0 | 0 | 0 | 0 |
| EX_glyc3p(e) | 0 | 0 | 0 | 0 | 0 | -0.32222 | 0 | 0 |
| EX_glyclt(e) | 0 | 0 | 0 | 0 | 0 | 0 | 0 | 0 |
| EX_glycogen(e) | 0 | 0 | 0 | 0 | 0 | 0 | 0 | 0 |
| EX_gmp(e) | -1.25968 | -1.19739 | -0.86378 | 0 | -0.00025 | 0 | -0.19699 | -0.02304 |
| EX_gsn(e) | 0 | 0 | 0 | 0 | 0 | 0 | 0 | 0 |
| EX_gthox(e) | 0 | 0 | 0 | 0 | 0 | 0 | 0 | 0 |
| EX_gthrd(e) | 0 | 0 | 0 | 0 | 0 | 0 | 0 | 0 |
| EX_gua(e) | 0 | 0 | 0 | -0.00383 | 0 | -0.00992 | 0 | 0 |
| EX_h2o2(e) | 0 | 0 | 0 | 0 | 0 | 0 | 0 | 0 |
| EX_hexs(e) | 0 | 0 | 0 | 0 | 0 | 0 | 0 | 0 |
| EX_hg2(e) | 0 | 0 | 0 | 0 | 0 | 0 | 0 | 0 |
| EX_his__L(e) | 0 | 0 | 0 | 0 | 0 | 0 | 0 | 0 |
| EX_hqn(e) | 0 | 0 | 0 | 0 | 0 | 0 | 0 | 0 |
| EX_hxan(e) | 0 | 0 | 0 | -0.03781 | 0 | 0 | 0 | 0 |
| EX_icit(e) | 0 | 0 | 0 | 0 | 0 | 0 | 0 | 0 |
| EX_ile__L(e) | -0.224 | -0.20122 | -0.15946 | -0.08352 | -0.12907 | 0 | -0.28474 | -0.06454 |
| EX_inost(e) | 0 | 0 | 0 | 0 | 0 | 0 | 0 | 0 |
| EX_ins(e) | 0 | 0 | 0 | 0 | 0 | 0 | 0 | 0 |
| EX_istnt(e) | 0 | 0 | 0 | 0 | 0 | 0 | 0 | 0 |
| EX_lac__L(e) | 0 | 0 | 0 | 0 | 0 | 0 | 0 | 0 |

| Reaction | Glucose | Fructose | Gluconate | Glutamate/ Succinate | Glycerol | Malate | Malate/ Glucose | Pyruvate |
|---|---|---|---|---|---|---|---|---|
| EX_lanth(e) | 0 | 0 | 0 | 0 | 0 | 0 | 0 | 0 |
| EX_lcts(e) | 0 | 0 | 0 | 0 | 0 | 0 | 0 | 0 |
| EX_leu__L(e) | 0 | 0 | 0 | 0 | 0 | 0 | 0 | 0 |
| EX_lipt(e) | 0 | 0 | 0 | 0 | 0 | 0 | 0 | 0 |
| EX_lys__L(e) | -0.19192 | -0.17241 | -0.13662 | -0.07156 | 0 | 0 | -0.24397 | -0.0553 |
| EX_madg(e) | 0 | 0 | 0 | 0 | 0 | 0 | 0 | 0 |
| EX_mal__D(e) | 0 | 0 | 0 | 0 | 0 | 0 | 0 | 0 |
| EX_malt(e) | 0 | 0 | 0 | 0 | 0 | 0 | 0 | 0 |
| EX_malttr(e) | 0 | 0 | -0.01575 | -0.16903 | -0.08622 | -0.03993 | -0.0039 | -0.02536 |
| EX_man1p(e) | 0 | 0 | 0 | 0 | 0 | 0 | 0 | 0.25541 |
| EX_man6p(e) | 0 | 0 | 0 | 0.71407 | 0.17121 | 0 | 0 | 0 |
| EX_man(e) | 0 | 0 | 0 | 0 | 0 | 0 | 0 | 0 |
| EX_mbdg(e) | 0 | 0 | 0 | 0 | 0 | 0 | 0 | 0 |
| EX_melib(e) | 0 | 0 | 0 | 0 | 0 | 0 | 0 | 0 |
| EX_meoh(e) | 0 | 0 | 0 | 0 | 0 | 0 | 0 | 0 |
| EX_met__D(e) | 0 | 0 | 0 | 0 | 0 | 0 | 0 | 0 |
| EX_met_L_ala__L(e) | 0 | 0 | 0 | 0 | 0 | 0 | 0 | 0 |
| EX_met__L(e) | -0.06686 | -0.06006 | -0.0476 | 0 | -0.06813 | 0 | -0.08499 | -0.01927 |
| EX_metox__R(e) | 0 | 0 | 0 | 0 | 0 | 0 | 0 | 0 |
| EX_metox(e) | 0 | 0 | 0 | 0 | 0 | 0 | 0 | 0 |
| EX_mn2(e) | 0 | 0 | 0 | 0 | 0 | 0 | 0 | 0 |
| EX_mnl(e) | 0 | 0 | 0 | 0 | 0 | 0 | 0 | 0 |
| EX_mobd(e) | 0 | 0 | 0 | 0 | 0 | 0 | 0 | 0 |
| EX_mops(e) | 0 | 0 | 0 | 0 | 0 | 0 | 0 | 0 |
| EX_mso3(e) | 0 | 0 | 0 | 0 | 0 | 0 | 0 | 0 |
| EX_nac(e) | 0 | 0 | 0 | -0.00381 | 0 | -0.00987 | 0 | -0.00294 |
| EX_ni2(e) | 0 | 0 | 0 | 0 | 0 | 0 | 0 | 0 |
| EX_no2(e) | 0 | 0 | 0 | 0 | 0 | 0 | 0 | 0 |

| Reaction | Glucose | Fructose | Gluconate | Glutamate/ Succinate | Glycerol | Malate | Malate/ Glucose | Pyruvate |
|---|---|---|---|---|---|---|---|---|
| EX_no3(e) | 0 | 0 | 0 | 0 | 0 | 0 | 0 | 0 |
| EX_orn__L(e) | 0 | 0 | 0 | 0 | 0 | 0 | 0 | 0 |
| EX_pala(e) | 0 | 0 | 0 | 0 | 0 | 0 | 0 | 0 |
| EX_pep(e) | 0 | 0 | 0 | 0 | 0 | 0 | 0 | 0 |
| EX_phe__L(e) | -0.04424 | -0.0316 | -0.04244 | 0 | 0 | 0 | 0 | 0 |
| EX_pnto__R(e) | 0 | 0 | 0 | 0 | 0 | 0 | 0 | 0 |
| EX_ppa(e) | 0 | 0 | 0 | 0 | 0.34583 | 0.97445 | 0 | 0 |
| EX_ppi(e) | 0 | 0 | 0 | 0 | 0 | 0 | 0 | 0 |
| EX_pro__L(e) | 0 | 0 | 0 | 0 | 0 | 0 | 0 | 0 |
| EX_prolb(e) | 0 | 0 | 0 | 0 | 0 | 0 | 0 | 0 |
| EX_pser__D(e) | 0 | 0 | 0 | 0 | 0 | 0 | 0 | 0 |
| EX_pser__L(e) | 0 | -0.13217 | 0 | 0 | 0 | -0.21103 | 0 | 0 |
| EX_pur(e) | 0 | 0 | 0 | 0 | 0 | 0 | 0 | 0 |
| EX_raffin(e) | 0 | 0 | 0 | -0.05536 | 0 | 0 | 0 | -0.04277 |
| EX_rib__D(e) | 1.4243 | 2.35997 | 0.95105 | 0.05566 | 0.1229 | 0.20614 | 0.54269 | 0.06318 |
| EX_ribflv(e) | 0 | 0 | 0 | 0 | 5e-04 | 0 | 0 | 0 |
| EX_rmn(e) | -0.28348 | -0.32153 | -0.23812 | -0.03255 | -1.95986 | 0 | -0.355 | -0.00719 |
| EX_salcn(e) | 0 | 0 | 0 | 0 | 0 | 0 | 0 | 0 |
| EX_sbt__D(e) | 0 | 0 | 0 | 0 | 0 | 0 | 0 | 0 |
| EX_ser__D(e) | 0 | 0 | 0 | 0 | 0 | 0 | 0 | 0 |
| EX_ser__L(e) | 0 | 0 | 0 | 0 | 0 | 0 | 0 | 0 |
| EX_spmd(e) | 0 | 0 | 0 | 0 | 0 | 0.14702 | 0 | 0 |
| EX_srb__L(e) | 0 | 0 | 0 | 0 | 0 | 0 | 0 | 0 |
| EX_starch(e) | 0 | 0 | 0 | 0 | 0 | 0 | 0 | 0 |
| EX_subtilisin(e) | 0 | 0 | 0 | 0 | 0 | 0 | 0 | 0 |
| EX_sucr(e) | 0 | 0 | 0 | -0.0046 | 0 | 0 | 0 | -0.00969 |
| EX_sula(e) | 0 | 0 | 0 | 0 | 0 | 0 | 0 | 0 |
| EX_taur(e) | 0 | 0 | 0 | 0 | 0 | 0 | 0 | 0 |

| Reaction | Glucose | Fructose | Gluconate | Glutamate/ Succinate | Glycerol | Malate | Malate/ Glucose | Pyruvate |
|---|---|---|---|---|---|---|---|---|
| EX_thiog(e) | 0 | 0 | 0 | 0 | 0 | 0 | 0 | 0 |
| EX_thr__L(e) | 0 | 0 | 0 | 0 | 0 | 0 | 0 | 0 |
| EX_thym(e) | -1.12478 | -0.9974 | -0.83769 | -0.23573 | -0.43991 | -1.3235 | -0.84341 | -0.13358 |
| EX_thymd(e) | 0 | 0 | 0 | 0 | 0 | 0 | 0 | 0 |
| EX_tmp(e) | 0 | 0 | 0 | 0 | 0 | 0 | 0 | 0 |
| EX_tre(e) | 0 | 0 | 0 | 0 | 0 | 0 | 0 | 0 |
| EX_trp__L(e) | -0.03206 | -0.0288 | -0.02282 | -0.01195 | -0.02173 | -0.03097 | -0.04075 | -0.00924 |
| EX_tyr__L(e) | 0 | 0 | 0 | 0 | 0.02204 | 0 | 0 | 0 |
| EX_ump(e) | 0 | 0 | 0 | 0 | 0 | 0 | 0 | 0 |
| EX_ura(e) | 0 | 0 | 0 | 0 | 0 | 0 | 0 | 0 |
| EX_urate(e) | 0 | 0 | 0 | 0 | 0 | 0 | 0 | 0 |
| EX_urea(e) | 0 | 0 | 0 | 0 | 0 | 0 | 0 | 0 |
| EX_uri(e) | 0 | 0 | 0 | 0 | 0 | 0 | 0 | 0 |
| EX_val__L(e) | -0.42728 | -0.16257 | -0.30416 | -0.11344 | 0 | 0 | 0 | -0.04149 |
| EX_xan(e) | 0 | 0 | 0 | 0 | 0 | 0 | 0 | 0 |
| EX_xtsn(e) | 0 | 0 | 0 | 0 | 0 | 0 | 0 | 0 |
| EX_xyl__D(e) | 0 | 0 | 0 | 0 | 0 | 0 | 0 | 0 |
| EX_zn2(e) | 0 | 0 | 0 | 0 | 0 | 0 | 0 | 0 |
| EX_f6p(e) | 0 | 0 | 0 | 0 | 0 | 0 | 0 | 0 |

**Table S3:** List of gene-protein-reaction (GPR) associations from the central energy metabolism of *B. subtilis* (only of genes showing significant differential expression and reactions for which Vmax > 0).

| Gene symbol | Gene ID | Flux name |
|---|---|---|
| ackA | BSU29470 | ACKr |
| citA | BSU09440 | CS |
| citB | BSU18000 | ACONT |
| citZ | BSU29140 | CS |
| dctP | BSU04470 | SUCCt2r |
| eno | BSU33900 | ENO |
| fbaA | BSU37120 | FBA |
| fbp | BSU40190 | FBP |
| fruA | BSU14400 | FRUpts |
| fumC | BSU33040 | FUM |
| gapA | BSU33940 | GAPD |
| gapB | BSU29020 | GAPDi_nadp |
| glpF | BSU09280 | GLYCt |
| gltP | BSU02340 | GLUt2r |
| gndA | BSU23860 | GND |
| gntP | BSU40070 | GLCNt2ir |
| gntZ | BSU40080 | GND |
| icd | BSU29130 | ICDHyr |
| levD | BSU27070 | FRUpts |
| levE | BSU27060 | FRUpts |
| levF | BSU27050 | FRUpts |
| levG | BSU27040 | FRUpts |
| ptsH | BSU13900 | FRUpts |
| ptsI | BSU13910 | FRUpts |
| lrgA | BSU28910 | PYRt2 |
| lrgB | BSU28900 | PYRt2 |
| maeA | BSU37050 | ME2 |
| maeN | BSU31580 | MALt4 |
| yflS | BSU07570 | MALt4 |
| maeN | BSU31580 | MALt10 |
| yflS | BSU07570 | MALt10 |
| maeN | BSU31580 | MALt2r |
| yflS | BSU07570 | MALt2r |
| malS | BSU29880 | ME2 |
| mdh | BSU29120 | MDH |
| mleA | BSU23550 | ME2 |
| odhA | BSU19370 | AKGDH |
| odhB | BSU19360 | AKGDH |
| pckA | BSU30560 | PPCK |

| Gene symbol | Gene ID | Flux name |
|---|---|---|
| pdhA | BSU14580 | PDH |
| pdhB | BSU14590 | PDH |
| pdhC | BSU14600 | PDH |
| pdhD | BSU14610 | PDH |
| pfkA | BSU29190 | PFK |
| pgi | BSU31350 | PGI |
| pgk | BSU33930 | PGK_1 |
| pgm | BSU33910 | PGM_1 |
| pps | BSU18830 | PPS |
| pta | BSU37660 | PTAr |
| ptsG | BSU13890 | GLCpts |
| ptsH | BSU13900 | GLCpts |
| ptsI | BSU13910 | GLCpts |
| pycA | BSU14860 | PC |
| pyk | BSU29180 | PYK |
| rpe | BSU15790 | RPE |
| sdhA | BSU28440 | SUCD1 |
| sdhB | BSU28430 | SUCD1 |
| sdhC | BSU28450 | SUCD1 |
| sucC | BSU16090 | SUCOAS |
| sucD | BSU16100 | SUCOAS |
| tkt | BSU17890 | TKT1 |
| tkt | BSU17890 | TKT2 |
| tpiA | BSU33920 | TPI |
| ykgB | BSU13010 | G6PDH2r |
| ytsJ | BSU29220 | ME2 |
| ywjH | BSU37110 | TALA |
| ywlF | BSU36920 | RPI |
| zwf | BSU23850 | G6PDH2r |
| fruK | BSU14390 | FRUK |
| glpK | BSU09290 | GLYK |
| serA | BSU23070 | PGCD |
| yoaD | BSU18560 | PGCD |
| sdaAB | BSU15850 | SERD_L |
| sdaAA | BSU15860 | SERD_L |
| fbaA | BSU37120 | FBA2 |
| gntK | BSU40060 | GNKr |
| nadB | BSU27870 | ASPO1 |
| aspB | BSU22370 | ASPTA |
| mtnE | BSU13580 | UNK5 |
| citM | BSU07610 | CITt10 |
| citM | BSU07610 | CITt14 |
| citM | BSU07610 | CITt15 |
| cimH | BSU38770 | CITt2r |
| yfiY | BSU08440 | FEDCabc |
| yfhA | BSU08460 | FEDCabc |

| Gene symbol | Gene ID | Flux name |
|---|---|---|
| yfiZ | BSU08450 | FEDCabc |
| citM | BSU07610 | ICITt10 |
| citM | BSU07610 | ICITt2 |
| prpB | BSU24120 | MCITL2 |
| metI | BSU11870 | SHSL1_1 |
| metI | BSU11870 | SHSL2 |
| metI | BSU11870 | SHSL4r |
| argH | BSU29440 | ARGSL |
| ansB | BSU23570 | ASPT |
| yflS | BSU07570 | FUMt2r |
| dctP | BSU04470 | FUMt2r |
| menD | BSU30820 | 2S6HCCi |
| argD | BSU11220 | ACOTA |
| yflS | BSU07570 | AKGt2r |
| yoaB | BSU18540 | AKGt2r |
| gudB | BSU22960 | GLUDxi |
| rocG | BSU37790 | GLUDxi |
| gltA | BSU18450 | GLUSy |
| gltB | BSU18440 | GLUSy |
| hisH | BSU34890 | HSTPTr |
| hisC | BSU22620 | HSTPTr |
| menD | BSU30820 | OXGDC |
| hisC | BSU22620 | PHETA1 |
| hisH | BSU34890 | PHETA1 |
| hisC | BSU22620 | TYRTA |
| hisH | BSU34890 | TYRTA |
| ywiE | BSU37240 | CLPNS2_BS |
| clsA | BSU36590 | CLPNS2_BS |
| yhdN | BSU09530 | ALCD19y |
| iolS | BSU39780 | ALCD19y |
| artP | BSU23980 | ARGabc |
| artQ | BSU23970 | ARGabc |
| artR | BSU23960 | ARGabc |
| rocC | BSU37760 | ARGt2r |
| dhaS | BSU19310 | LCADi |
| ldh | BSU03050 | LDH_L |
| lctP | BSU03060 | L_LACt2r |
| yvfH | BSU34190 | L_LACt2r |
| trpB | BSU22640 | TRPS1 |
| trpA | BSU22630 | TRPS1 |
| cysK | BSU00730 | CYSS_2 |
| mccB | BSU27250 | CYSTGL_1 |
| mccB | BSU27250 | TRPAS1 |
| hisD | BSU34910 | HISTD |
| ybgF | BSU02400 | HISt2r |
| tdh | BSU16990 | THRD |

| Gene symbol | Gene ID | Flux name |
|---|---|---|
| ilvA | BSU21770 | THRD_L |
| thrC | BSU32250 | THRS |
| pssA | BSU02270 | CDPDSP_BS |
| mccA | BSU27260 | CYSTS_2 |
| glyA | BSU36900 | GHMT2r |
| cysE | BSU00930 | SERAT |
| dhaS | BSU19310 | ALDD31_1 |
| thiO | BSU11670 | GLYO1 |
| purD | BSU06530 | PRAGSr |

**Table S4:** Initial and final bounds after the search space reduction process.

| Reaction | Minimum flux (Before) | Maximum flux (Before) | Minimum flux (After) | Maximum flux (After) |
|---|---|---|---|---|
| 2S6HCCi | 5e-05 | 0.00028 | 5e-05 | 0.00028 |
| ACKr | -210.19189 | 209.68905 | -0.41053 | 0.40955 |
| ACONT | -6.02243 | 45.16508 | -3.01122 | 22.58254 |
| ACOTA | -136.19379 | 0 | -0.0665 | 0 |
| AKGDH | 0 | 44.6085 | 0 | 22.30425 |
| AKGt2r | 0 | 0.68788 | 0 | 0.68788 |
| ALCD19y | -6.22024 | 5.72 | -6.22024 | 5.72 |
| ALDD31_1 | 0 | 0.68788 | 0 | 0.68788 |
| ARGSL | -0.65506 | 42.09664 | -0.00128 | 0.08222 |
| ARGabc | 0 | 0.68788 | 0 | 0.68788 |
| ARGt2r | 0 | 0.68788 | 0 | 0.68788 |
| ASPO1 | 0 | 118.66166 | 0 | 29.66541 |
| ASPT | 0 | 185.0167 | 0 | 0.00565 |
| ASPTA | -186.21329 | 0.3619 | -46.55332 | 0.09048 |
| CDPDSP_BS | 0.00952 | 0.059 | 0.00952 | 0.059 |
| CITt10 | 0 | 0.68788 | 0 | 0.68788 |
| CITt14 | 0 | 839.79376 | 0 | 8e-04 |
| CITt15 | 0 | 839.79376 | 0 | 0.10251 |
| CITt2r | -839.79193 | 0.68788 | -0.10251 | 8e-05 |
| CLPNS2_BS | 8e-05 | 0.00053 | 8e-05 | 0.00053 |
| CS | 0 | 45.16504 | 0 | 22.58252 |
| CYSS_2 | 0 | 78.8513 | 0 | 0.0385 |
| CYSTGL_1 | 0 | 25.07966 | 0 | 0.01225 |
| CYSTS_2 | 0 | 118.27695 | 0 | 0.0018 |
| ENO | -116.33363 | 28.31823 | -29.08341 | 7.07956 |
| FRUK | 0 | 6.22024 | 0 | 6.22024 |
| FBA | -23.36851 | 9.13029 | -11.68426 | 4.56514 |
| FBA2 | -6.22024 | 5.72 | -6.22024 | 5.72 |
| FBP | 0 | 23.36851 | 0 | 0.02282 |
| FEDCabc | 0 | 0.00364 | 0 | 0.00364 |
| FRUpts | 0 | 5.72 | 0 | 5.72 |
| FUM | -15.42879 | 208.51309 | -1.9286 | 26.06414 |
| FUMt2r | -17.13865 | 0.68788 | -0.1339 | 0.00537 |
| G6PDH2r | 0 | 72.07422 | 0 | 0.03519 |
| GAPD | 0 | 17.68403 | 0 | 17.68403 |
| GAPDi_nadp | 0 | 30.62267 | 0 | 0.0299 |
| GHMT2r | 6e-05 | 48.38152 | 0 | 0.0945 |
| GLCNt2ir | 0 | 5.13 | 0 | 5.13 |
| GLCpts | 0 | 9.00576 | 0 | 9.00576 |
| GLUDxi | 0 | 132.77219 | 0 | 0.0081 |
| GLUSy | 0 | 135.9922 | 0 | 0.00104 |
| GLUt2r | 0 | 3.58576 | 0 | 3.58576 |
| GLYCt | -2.92906 | 6.22 | -2.92906 | 6.22 |

| Reaction | Minimum flux (Before) | Maximum flux (Before) | Minimum flux (After) | Maximum flux (After) |
|---|---|---|---|---|
| GLYK | 0 | 6.22024 | 0 | 6.22024 |
| GLYO1 | 0 | 49.7265 | 0 | 3.10791 |
| GNKr | 0 | 5.13 | 0 | 5.13 |
| HISTD | 0 | 17.25995 | 0 | 2e-05 |
| HISt2r | 0 | 0.68788 | 0 | 0.68788 |
| HSTPTr | 0 | 17.25995 | 0 | 0.01686 |
| ICDHyr | -6.02243 | 45.16508 | -3.01122 | 22.58254 |
| ICITt10 | 0 | 0.68788 | 0 | 0.68788 |
| ICITt2 | -11.2552 | 0.68788 | -5.6276 | 0.34394 |
| LCADi | 0 | 0.68788 | 0 | 0.68788 |
| LDH_L | -17.49439 | 0.68788 | -4.3736 | 0.17197 |
| L_LACt2r | -17.4957 | 27.19788 | -4.37393 | 6.79947 |
| MALt10 | 0 | 26.51 | 0 | 1.65688 |
| MALt2r | 0 | 26.51 | 0 | 26.51 |
| MALt4 | 0 | 26.51 | 0 | 0.02589 |
| MDH | -185.0167 | 138.68804 | -23.12709 | 17.336 |
| ME2 | 0 | 235.02309 | 0 | 0.00045 |
| MCITL2 | 0 | 24.86325 | 0 | 0.01214 |
| OXGDC | 0 | 44.4981 | 0 | 0.02173 |
| PC | 0 | 209.93311 | 0 | 0.0032 |
| PDH | 0 | 24.86325 | 0 | 24.86325 |
| PFK | 0 | 9.12999 | 0 | 9.12999 |
| PGCD | -0.55455 | 118.79942 | -0.00108 | 0.23203 |
| GND | -6.15562 | 72.07422 | -0.38473 | 4.50464 |
| PGI | -72.21764 | 9.54518 | -9.0272 | 1.19315 |
| PGK_1 | -30.62269 | 17.68403 | -30.62269 | 17.68403 |
| PGM_1 | -116.33363 | 28.31823 | -29.08341 | 7.07956 |
| PHETA1 | -3.97414 | 0 | -3.97414 | 0 |
| PPCK | 0 | 235.0073 | 0 | 3.67199 |
| PPS | 0 | 209.93265 | 0 | 0.0032 |
| PRAGSr | 0 | 18.7662 | 0 | 0.00229 |
| PTAr | -210.19189 | 209.68905 | -0.41053 | 0.40955 |
| PYK | 0 | 236.48892 | 0 | 14.78056 |
| PYRt2 | -20.82133 | 8.26 | -10.41067 | 4.13 |
| RPE | -25.10204 | 47.8109 | -0.78444 | 1.49409 |
| RPI | -25.10204 | 1.8482 | -1.56888 | 0.11551 |
| SERAT | 0 | 78.8513 | 0 | 0.0385 |
| SERD_L | 0 | 118.24446 | 0 | 9e-04 |
| SHSL1_1 | 0 | 25.14425 | 0 | 0.00614 |
| SHSL2 | 0 | 25.14425 | 0 | 0.09822 |
| SHSL4r | 0 | 25.07966 | 0 | 0.02449 |
| SUCCt2r | -13.87046 | 3.35 | -13.87046 | 3.35 |
| SUCD1 | -0.68788 | 48.04948 | -0.34394 | 24.02474 |
| SUCOAS | -187.05087 | 44.5412 | -23.38136 | 5.56765 |
| TALA | -12.45362 | 24.00274 | -0.38918 | 0.75009 |
| THRD | 0 | 43.88868 | 0 | 10.97217 |

| Reaction | Minimum flux (Before) | Maximum flux (Before) | Minimum flux (After) | Maximum flux (After) |
|---|---|---|---|---|
| THRD_L | 0 | 25.07966 | 0 | 0.09797 |
| THRS | 0 | 44.20647 | 0 | 5.52581 |
| TKT1 | -12.45362 | 24.00274 | -0.77835 | 1.50017 |
| TKT2 | -12.64841 | 23.80816 | -0.79053 | 1.48801 |
| TPI | -23.71431 | 12.67605 | -11.85715 | 6.33802 |
| TRPAS1 | 0 | 78.89095 | 0 | 0.07704 |
| TRPS1 | 0 | 0.05725 | 0 | 0.05725 |
| TYRTA | -3.93702 | 0 | -3.93702 | 0 |
| UNK5 | 0 | 1.3786 | 0 | 1.3786 |

**Table S5:** List of average Pearson's correlation coefficients (r) and average normalized errors between $^{13}C$ metabolic flux data. and mCADRE flux prediction results using different percentile cut-offs

| **Percentile cut-off** | **Average r** | **Average normalized error** |
|---|---|---|
| 25 | -0.3 | 132.82 |
| 50 | -0.3 | 132.82 |
| 65 | -0.2 | 125.55 |
| 75 | 0.04 | 68.04 |
| 85 | -0.16 | 31.35 |

**Table S6:** Predicted flux from RED-TIL before implementing IFFPR.

| Reaction | Glucose | Fructose | Gluconate | Glutamate/ Succinate | Glycerol | Malate | Malate/ Glucose | Pyruvate |
|---|---|---|---|---|---|---|---|---|
| 2S6HCCi | 0.00016 | 0.00014 | 0.00011 | 6e-05 | 0.00011 | 0.00015 | 2e-04 | 5e-05 |
| ACKr | -0.28562 | -0.25657 | -2.26591 | -0.1065 | 2.46979 | -0.27594 | 19.56525 | -0.07261 |
| ACONT | 17.26898 | 19.1058 | 3.87967 | 15.74668 | 2.52468 | 27.31511 | -1.62242 | 7.63315 |
| ACOTA | -0.11388 | 0 | 0 | -0.04246 | 0 | 0 | 0 | 0 |
| AKGDH | 18.90287 | 20.90058 | 2.93434 | 16.41885 | 4.04551 | 27.83383 | 0 | 7.40157 |
| AKGt2r | 0 | 0 | 0 | 0 | 0 | 0 | 0 | 0 |
| ALCD19y | -0.31052 | 4.72214 | -0.54423 | -0.36273 | 0 | -0.82612 | -0.63553 | -0.48972 |
| ALDD31_1 | 0 | 0 | 0 | 0 | 0 | 0 | 0 | 0 |
| ARGSL | 0.11388 | 0 | 0 | 0.04246 | 0 | 0 | 0 | 0 |
| ARGabc | 0 | 0 | 0 | 0 | 0 | 0 | 0 | 0 |
| ARGt2r | 0 | 0 | 0 | 0 | 0 | 0 | 0 | 0 |
| ASPO1 | 46.84012 | 54.13017 | 0 | 45.2078 | 0 | 39.5407 | 0 | 2.40756 |
| ASPT | 0 | 0 | 1.62029 | 0 | 0.20567 | 0 | 0 | 0 |
| ASPTA | -48.02375 | -57.57759 | -2.60015 | -51.17198 | -0.88447 | -43.79949 | -1.28574 | -2.69899 |
| CDPDSP_BS | 0.03304 | 0.02968 | 0.02352 | 0.01232 | 0.0224 | 0.03192 | 0.042 | 0.00952 |
| CITt10 | 0 | 0 | 0 | 0 | 0 | 0 | 0 | 0 |
| CITt14 | 0.00189 | 0.0017 | 0.00135 | 0.00071 | 0.00128 | 0.00183 | 0.0024 | 0.00054 |
| CITt15 | 0.41672 | 0.37435 | 0 | 0.15539 | 0 | 0.4026 | 0 | 0.12007 |
| CITt2r | -0.42269 | -0.3797 | -0.00424 | -0.15761 | -0.65426 | -0.40511 | -1.62728 | -0.12179 |
| CLPNS2_BS | 0.00029 | 0.00026 | 0.00021 | 0.00011 | 2e-04 | 0.00028 | 0.00038 | 8e-05 |
| CS | 17.26898 | 19.1058 | 3.87967 | 15.74668 | 3.17765 | 27.31511 | 0 | 7.63315 |
| CYSS_2 | 0.03362 | 0.0302 | 0 | 0.01254 | 0 | 0.03248 | 0 | 0 |
| CYSTGL_1 | 0 | 0 | 0.15946 | 0 | 0.0228 | 0 | 0.04274 | 0 |
| CYSTS_2 | 0 | 0 | 0.02394 | 0 | 0.0228 | 0 | 0.04274 | 0 |

| Reaction | Glucose | Fructose | Gluconate | Glutamate/ Succinate | Glycerol | Malate | Malate/ Glucose | Pyruvate |
|---|---|---|---|---|---|---|---|---|
| ENO | 25.17167 | 25.79165 | 11.74637 | 18.18548 | 16.40287 | 13.90917 | 16.34064 | -2.74637 |
| FRUK | 1.18699 | 0.99786 | 0.54423 | 2.63898 | 0.78677 | 1.31452 | 1.05034 | 0.52508 |
| FBA | 10.31698 | 8.68986 | 4.56295 | 7.53391 | 5.09392 | 6.41429 | 8.25103 | -1.66235 |
| FBA2 | 1.0937 | 4.72214 | -0.54423 | -0.29755 | 0 | -0.82612 | -0.63553 | -0.48972 |
| FBP | 0 | 0 | 0 | 0 | 0 | 0 | 0 | 2.18743 |
| FEDCabc | 0.00204 | 0.00183 | 0.00145 | 0.00076 | 0 | 0.00034 | 0.00123 | 0.00059 |
| FRUpts | 2.28068 | 5.72 | 0 | 2.34143 | 0.78677 | 0.48841 | 0.41481 | 0.03536 |
| FUM | 19.06498 | 21.25129 | 6.47981 | 19.82929 | 5.05994 | 13.96337 | -11.14188 | 6.97985 |
| FUMt2r | 0 | 0 | 0 | 0 | 0 | 0 | -11.14188 | -3.77172 |
| G6PDH2r | 1.65298 | 1.06168 | 0 | 0 | 0 | 0 | 2.8369 | 0 |
| GAPD | 24.12271 | 24.75697 | 11.93238 | 15.49136 | 16.58003 | 12.52081 | 16.67281 | 0 |
| GAPDi_nadp | 0 | 0 | 0 | 0 | 0 | 0 | 0 | 2.70003 |
| GHMT2r | 0.12897 | 0.06026 | 0.04775 | 0.04809 | 0.04548 | 0.06481 | 0.08527 | 6e-05 |
| GLCNt2ir | 2.11047 | 1.14314 | 5.13 | 0.8888 | 1.05032 | 1.38732 | 1.17797 | 0 |
| GLCpts | 8.79082 | 6.27277 | 0.59994 | 3.61337 | 1.76104 | 3.32104 | 7.83926 | 0 |
| GLUDxi | 0 | 0 | 0 | 0 | 0 | 0 | 0 | 0 |
| GLUSy | 19.17815 | 11.72376 | 1.38737 | 0 | 0 | 21.58601 | 0.96324 | 0 |
| GLUt2r | 0.56656 | 1.17465 | 1.19307 | 3.50561 | 0.86529 | 0.60063 | 0 | 3.0638 |
| GLYCt | 1.96619 | -2.47656 | 2.388 | 3.75833 | 6.22 | 2.90501 | 0.63515 | 2.75541 |
| GLYK | 1.65597 | 2.24584 | 1.84398 | 3.39571 | 6.2202 | 2.07918 | 0 | 2.26578 |
| GLYO1 | 0 | 2.53883 | 0 | 5.52283 | 0 | 3.28162 | 0 | 0 |
| GNKr | 2.11047 | 1.14314 | 5.13 | 0.8888 | 1.05032 | 1.38732 | 1.17797 | 0 |
| HISTD | 0.04823 | 0 | 0 | 0.01798 | 0 | 0 | 0 | 0 |
| HISt2r | 0 | 0.04332 | 0.03433 | 0 | 0.0327 | 0.04659 | 0.0613 | 0.0139 |
| HSTPTr | 0.04823 | 0 | 0 | 0.01798 | 0 | 0 | 0 | 0 |
| ICDHyr | 17.26898 | 19.1058 | 1.05338 | 13.12806 | 2.52468 | 26.07039 | -1.62242 | 4.47096 |
| ICITt10 | 0.06001 | 0.05391 | 0.04272 | 0.02238 | 0.04069 | 0.05798 | 0 | 0.01729 |
| ICITt2 | -0.06001 | -0.05391 | -2.869 | -2.641 | -0.04069 | -1.30269 | 0 | -3.17948 |

| Reaction | Glucose | Fructose | Gluconate | Glutamate/ Succinate | Glycerol | Malate | Malate/ Glucose | Pyruvate |
|---|---|---|---|---|---|---|---|---|
| LCADi | 0 | 0 | 0 | 0 | 0 | 0 | 0 | 0 |
| LDH_L | -7.59968 | -12.42649 | -6.40277 | -9.87178 | -13.74103 | -4.37613 | -15.01479 | -2.00669 |
| L_LACt2r | -7.59968 | -11.0667 | -6.40277 | -8.23782 | -12.93366 | 17.18356 | -10.50732 | -0.12647 |
| MALt10 | 0 | 1.3598 | 0 | 1.63396 | 0.80737 | 21.55969 | 4.50746 | 1.88022 |
| MALt2r | 0 | 1.3598 | 0 | 1.63396 | 0.80737 | 0 | 19.70507 | 1.88022 |
| MALt4 | 0 | 1.3598 | 0 | 1.63396 | 0.80737 | 4.95031 | 2.29746 | 1.88022 |
| MDH | 18.45261 | 22.55322 | 6.47981 | 21.71086 | 7.48205 | 31.57389 | 14.74815 | 10.71682 |
| ME2 | 0.61237 | 2.77747 | 0 | 3.02033 | 0 | 8.89948 | 0.61997 | 1.9037 |
| MCITL2 | 0 | 0 | 0 | 0 | 0 | 0 | 0 | 0 |
| OXGDC | 0 | 0 | 0 | 0 | 0 | 0 | 0 | 0 |
| PC | 0 | 0 | 0 | 0 | 0 | 0 | 0 | 0 |
| PDH | 18.32127 | 17.25636 | 0 | 10.61623 | 6.35895 | 24.86325 | 21.14455 | 7.82367 |
| PFK | 9.12999 | 7.692 | 4.01872 | 4.89493 | 4.30715 | 5.09977 | 7.20069 | 0 |
| PGCD | 0.35526 | 0.23473 | 0.18602 | 0.13247 | 0.17716 | 0.25245 | 0.33217 | 0.04634 |
| GND | 3.76345 | 2.20483 | 5.13 | -3.81354 | 1.05032 | -3.43116 | 4.01486 | -3.14088 |
| PGI | 6.98939 | 6.40157 | 0.49426 | 7.57464 | 1.6604 | 7.48658 | 4.81365 | -0.04277 |
| PGK_1 | 24.12271 | 24.75697 | 11.93238 | 15.49136 | 16.58003 | 12.52081 | 16.67281 | -2.70003 |
| PGM_1 | 25.17167 | 25.79165 | 11.74637 | 18.18548 | 16.40287 | 13.90917 | 16.34064 | -2.74637 |
| PHETA1 | -0.1038 | 0 | 0 | -0.03871 | 0 | -0.10029 | -0.10674 | 0 |
| PPCK | 0 | 0 | 0 | 0 | 3.41993 | 0 | 13.46241 | 2.79223 |
| PPS | 0 | 0 | 0 | 0 | 0 | 0 | 0 | 0 |
| PRAGSr | 0 | 0 | 0 | 0 | 0 | 0 | 0 | 0 |
| PTAr | -0.28562 | -0.25657 | -2.26591 | -0.1065 | 2.46979 | -0.27594 | 19.56525 | -0.07261 |
| PYK | 13.63728 | 13.74464 | 11.241 | 12.05808 | 17.36505 | 9.96788 | 21.25874 | 0 |
| PYRt2 | 2.32471 | 2.34571 | 0 | 0.09799 | 1.37828 | 8.26 | 8.26 | 8.26 |
| RPE | 2.33352 | 1.46372 | 3.52446 | -2.60778 | 0.81697 | -2.3275 | 2.63227 | -2.09588 |
| RPI | -1.42993 | -0.74111 | -1.60554 | 1.20576 | -0.23335 | 1.10366 | -1.38259 | 1.045 |
| SERAT | 0.03362 | 0.0302 | 0 | 0.01254 | 0 | 0.03248 | 0 | 0 |

| Reaction | Glucose | Fructose | Gluconate | Glutamate/ Succinate | Glycerol | Malate | Malate/ Glucose | Pyruvate |
|---|---|---|---|---|---|---|---|---|
| SERD_L | 0 | 0 | 0 | 0 | 0 | 0 | 0 | 0 |
| SHSL1_1 | 0 | 0 | 0.13552 | 0 | 0 | 0 | 0 | 0 |
| SHSL2 | 0.06686 | 0.06006 | 0.07153 | 0.02493 | 0.06813 | 0.0646 | 0.12774 | 0 |
| SHSL4r | 0 | 0 | 0 | 0 | 0 | 0 | 0 | 0 |
| SUCCt2r | 0 | 0.35071 | 1.92519 | 3.35 | 0.80876 | -13.87046 | 0 | 3.35 |
| SUCD1 | 18.90287 | 21.25129 | 4.85952 | 19.76885 | 4.85427 | 13.96337 | 0 | 10.75157 |
| SUCOAS | 18.83601 | 20.84051 | 2.72728 | 16.39391 | 3.97739 | 27.76924 | -0.12774 | 7.40157 |
| TALA | 1.26746 | 0.73193 | 1.76223 | -1.26634 | 0.40849 | -1.11353 | 1.36961 | -1.04794 |
| THRD | 0.11192 | 2.69496 | 0.39085 | 5.56456 | 0.11784 | 3.44954 | 0.22095 | 0.06935 |
| THRD_L | 0.224 | 0.20122 | 0 | 0.08352 | 0.12907 | 0.2164 | 0.242 | 0.06454 |
| THRS | 0.44584 | 2.99493 | 0.4691 | 5.68907 | 0.32143 | 3.77214 | 0.60268 | 0.16556 |
| TKT1 | 1.26746 | 0.73193 | 1.76223 | -1.26634 | 0.40849 | -1.11353 | 1.36961 | -1.04794 |
| TKT2 | 1.06606 | 0.73179 | 1.76223 | -1.34144 | 0.40849 | -1.21397 | 1.26267 | -1.04794 |
| TPI | 12.70871 | 15.33631 | 5.61518 | 9.28735 | 11.07838 | 7.32155 | 7.16051 | 0.01057 |
| TRPAS1 | 0 | 0 | 0 | 0 | 0 | 0 | 0 | 0 |
| TRPS1 | 0.03206 | 0 | 0 | 0.01195 | 0 | 0 | 0 | 0 |
| TYRTA | -0.06539 | 0 | 0 | -0.02438 | 0 | 0 | 0 | 0 |
| UNK5 | 0 | 0 | 0 | 0 | 0 | 0 | 0 | 0 |

**Table S7:** Predicted flux from RED-TIL after implementing IFFPR (LPM-GEM).

| Reaction | Glucose | Fructose | Gluconate | Glutamate/ Succinate | Glycerol | Malate | Malate/ Glucose | Pyruvate |
|---|---|---|---|---|---|---|---|---|
| 2S6HCCi | 0.00016 | 0.00014 | 0.00011 | 6e-05 | 0.00011 | 0.00015 | 2e-04 | 5e-05 |
| ACKr | -0.28438 | -0.25531 | -2.65871 | -0.10555 | 1.76043 | 9.90946 | 18.957 | -0.07261 |
| ACONT | 17.24565 | 19.30042 | 2.59554 | 8.1623 | 1.83771 | 13.95744 | -1.62242 | 7.63315 |
| ACOTA | -0.11388 | 0 | 0 | -0.04246 | 0 | 0 | 0 | 0 |
| AKGDH | 18.0321 | 19.24829 | 4.24718 | 6.77297 | 3.23589 | 13.87046 | 0 | 3.77844 |
| AKGt2r | 0 | 0 | 0 | 0 | 0 | 0 | 0 | 0 |
| ALCD19y | 2.40937 | 4.72214 | -0.54423 | -0.36273 | 0 | -0.82612 | -0.63553 | -0.48972 |
| ALDD31_1 | 0 | 0 | 0 | 0 | 0 | 0 | 0 | 0 |
| ARGSL | 0.11388 | 0 | 0 | 0.04246 | 0 | 0 | 0 | 0 |
| ARGabc | 0 | 0 | 0 | 0 | 0 | 0 | 0 | 0 |
| ARGt2r | 0 | 0 | 0 | 0 | 0 | 0 | 0 | 0 |
| ASPO1 | 29.66541 | 24.31364 | 0 | 21.47431 | 0 | 20.09286 | 0 | 0.65039 |
| ASPT | 0 | 0 | 0.00092 | 0 | 0.00073 | 0 | 0 | 1.75717 |
| ASPTA | -30.84904 | -27.95568 | -1.19046 | -21.91566 | -0.67953 | -21.07003 | -1.28574 | -2.69899 |
| CDPDSP_BS | 0.03304 | 0.02968 | 0.02352 | 0.01232 | 0.0224 | 0.03192 | 0.042 | 0.00952 |
| CITt10 | 0.06001 | 0 | 0 | 0.02238 | 0 | 0.05798 | 0 | 0.01729 |
| CITt14 | 0.00189 | 0.0017 | 0.00135 | 0.00071 | 0.00128 | 0.00183 | 0.0024 | 0.00054 |
| CITt15 | 0.01362 | 0.08378 | 0 | 0 | 0 | 0.02195 | 0 | 0 |
| CITt2r | -0.07959 | -0.08913 | -2.37278 | -2.09494 | -4.16207 | -0.08244 | -2.20219 | -0.01901 |
| CLPNS2_BS | 0.00029 | 0.00026 | 0.00021 | 0.00011 | 2e-04 | 0.00028 | 0.00038 | 8e-05 |
| CS | 17.24565 | 19.30042 | 4.96422 | 10.23264 | 5.99851 | 13.95744 | 0.57492 | 7.63315 |
| CYSS_2 | 0.03239 | 0.02894 | 0 | 0.01158 | 0.01875 | 0.02667 | 0.03333 | 0 |
| CYSTGL_1 | 0.00668 | 0.00598 | 0.02553 | 0.00506 | 0.00405 | 0.00909 | 0.00941 | 0 |
| CYSTS_2 | 0.00124 | 0.00126 | 0.02394 | 0.00095 | 0.00405 | 0.00582 | 0.00941 | 0 |

| Reaction | Glucose | Fructose | Gluconate | Glutamate/ Succinate | Glycerol | Malate | Malate/ Glucose | Pyruvate |
|---|---|---|---|---|---|---|---|---|
| ENO | 21.45335 | 22.06447 | 10.08625 | 12.44567 | 16.02458 | 10.53099 | 16.5552 | 0.06956 |
| FRUK | 0 | 0.99786 | 0.54423 | 2.70416 | 0.78677 | 1.31452 | 1.05034 | 0.40919 |
| FBA | 9.36225 | 8.68986 | 4.345 | 8.70341 | 4.8981 | 7.34887 | 8.79482 | 0.10609 |
| FBA2 | 2.40937 | 4.72214 | -0.54423 | -0.36273 | 0 | -0.82612 | -0.63553 | -0.37382 |
| FBP | 0 | 0 | 0 | 0 | 0 | 0 | 0 | 0.3031 |
| FEDCabc | 0.00204 | 0.00183 | 0.00138 | 0.00076 | 0 | 0.00034 | 0.00123 | 0.00059 |
| FRUpts | 2.40937 | 5.72 | 0 | 2.34143 | 0.78677 | 0.48841 | 0.41481 | 0.03536 |
| FUM | 18.14598 | 19.59901 | 6.1547 | 10.12915 | 2.42786 | -0.1339 | -9.91651 | 8.84991 |
| FUMt2r | 0 | 0 | -0.01858 | -0.04548 | 0 | -0.1339 | -9.91651 | -0.0357 |
| G6PDH2r | 0.03519 | 0 | 0 | 0 | 0 | 0 | 0.23321 | 0 |
| GAPD | 21.76039 | 20.93248 | 10.22622 | 12.56935 | 16.18848 | 10.78344 | 16.88737 | 0 |
| GAPDi_nadp | 0 | 0 | 0 | 0 | 0 | 0 | 0 | 0 |
| GHMT2r | 0.08075 | 0.06026 | 0.0017 | 0.0393 | 0.04548 | 0.06481 | 0.08527 | 6e-05 |
| GLCNt2ir | 2.01161 | 1.14314 | 5.13 | 0.8888 | 1.05032 | 1.38732 | 0.95337 | 0 |
| GLCpts | 8.69437 | 6.27277 | 0.59994 | 3.61337 | 1.76104 | 3.32104 | 7.63 | 0 |
| GLUDxi | 0 | 0 | 0 | 0 | 0 | 0 | 0 | 0 |
| GLUSy | 0.00104 | 0.00086 | 0 | 0 | 0 | 0.00062 | 0 | 0 |
| GLUt2r | 0.56656 | 1.17465 | 1.19307 | 3.48803 | 0.86529 | 0.60063 | 0 | 3.0638 |
| GLYCt | -1.96619 | -2.47656 | 2.388 | 3.75833 | 6.22 | 2.90501 | 0.63515 | 2.75541 |
| GLYK | 0.44347 | 2.24584 | 1.84398 | 3.39571 | 6.2202 | 2.07918 | 0 | 2.26578 |
| GLYO1 | 0 | 2.73345 | 0 | 0 | 0 | 0 | 0 | 0 |
| GNKr | 2.01161 | 1.14314 | 5.13 | 0.8888 | 1.05032 | 1.38732 | 0.95337 | 0 |
| HISTD | 0 | 0 | 0 | 0.00919 | 0 | 0 | 0 | 0 |
| HISt2r | 0.04823 | 0.04332 | 0.03433 | 0.00879 | 0.0327 | 0.04659 | 0.0613 | 0.0139 |
| HSTPTr | 0 | 0 | 0 | 0.00919 | 0 | 0 | 0 | 0 |
| ICDHyr | 16.39821 | 17.45352 | 2.29875 | 3.49976 | 1.83771 | 12.10702 | -1.62242 | 0.84783 |
| ICITt10 | 0 | 0.05391 | 0.04272 | 0 | 0.04069 | 0 | 0 | 0 |
| ICITt2 | -0.84743 | -1.90081 | -0.3395 | -4.66254 | -0.04069 | -1.85041 | 0 | -6.78532 |

| Reaction | Glucose | Fructose | Gluconate | Glutamate/ Succinate | Glycerol | Malate | Malate/ Glucose | Pyruvate |
|---|---|---|---|---|---|---|---|---|
| LCADi | 0 | 0 | 0 | 0 | 0 | 0 | 0 | 0 |
| LDH_L | -1.89491 | -2.85166 | -4.00767 | -2.25549 | -4.3736 | 0 | -7.20753 | -1.29442 |
| L_LACt2r | -1.89491 | -2.76667 | -4.00767 | -2.15337 | -3.68141 | 1.61909 | -5.97596 | -1.17691 |
| MALt10 | 0 | 0.08499 | 0 | 0.10212 | 0.69219 | 1.61909 | 1.23157 | 0.11751 |
| MALt2r | 0 | 1.3598 | 0 | 1.63396 | 0.80737 | 24.8812 | 19.70507 | 1.88022 |
| MALt4 | 0 | 0.00133 | 0 | 0.0016 | 0.00079 | 0.00971 | 0.01924 | 0.00184 |
| MDH | 18.14598 | 21.04512 | 6.15467 | 11.86682 | 3.92821 | 26.37572 | 6.05529 | 10.84919 |
| ME2 | 0 | 0 | 2e-05 | 1e-05 | 0 | 0.00039 | 4.98408 | 3e-04 |
| MCITL2 | 0 | 0 | 0 | 0 | 0 | 0 | 0 | 0 |
| OXGDC | 0 | 0 | 0 | 0 | 0 | 0 | 0 | 0 |
| PC | 0.28329 | 1.89733 | 0 | 0 | 2.74983 | 0 | 0 | 0 |
| PDH | 18.24971 | 17.25636 | 0 | 10.61623 | 8.48919 | 24.86325 | 21.14455 | 7.82367 |
| PFK | 9.36225 | 7.692 | 3.80078 | 5.99926 | 4.11133 | 6.03435 | 7.74448 | 0 |
| PGCD | 0.30703 | 0.23473 | 0.13997 | 0.12368 | 0.17716 | 0.25245 | 0.33217 | 0.04634 |
| GND | 2.0468 | 1.14314 | 1.49772 | -2.16584 | 1.05032 | -1.58063 | 1.18658 | -0.31437 |
| PGI | 8.51072 | 7.10936 | 1.00459 | 5.29615 | 1.6604 | 5.70133 | 7.20808 | -0.04277 |
| PGK_1 | 21.76039 | 20.93248 | 10.22622 | 12.56935 | 16.18848 | 10.78344 | 16.88737 | 0 |
| PGM_1 | 21.45335 | 22.06447 | 10.08625 | 12.44567 | 16.02458 | 10.53099 | 16.5552 | 0.06956 |
| PHETA1 | -0.1038 | 0 | 0 | -0.03871 | 0 | -0.06711 | -0.00211 | 0 |
| PPCK | 0 | 0 | 0 | 1.19283 | 0 | 11.44112 | 4.19463 | 1.16744 |
| PPS | 0 | 0 | 0 | 0 | 0 | 0 | 0 | 0 |
| PRAGSr | 0 | 0 | 0 | 0 | 0 | 0 | 0 | 0 |
| PTAr | -0.28438 | -0.25531 | -2.65871 | -0.10555 | 1.76043 | 9.90946 | 18.957 | -0.07261 |
| PYK | 9.88674 | 10.01746 | 9.63141 | 7.51111 | 13.56683 | 17.97011 | 12.62404 | 1.19113 |
| PYRt2 | 1.16235 | 1.17285 | 0 | 0.049 | 0.68914 | 4.31954 | 4.93258 | 8.26 |
| RPE | 1.22113 | 0.75593 | 0.9984 | -1.50346 | 0.8169 | -1.08275 | 0.78162 | -0.21155 |
| RPI | -0.82567 | -0.38721 | -0.49931 | 0.66239 | -0.23342 | 0.49788 | -0.40495 | 0.10283 |
| SERAT | 0.03239 | 0.02894 | 0 | 0.01158 | 0.01875 | 0.02667 | 0.03333 | 0 |

| Reaction | Glucose | Fructose | Gluconate | Glutamate/ Succinate | Glycerol | Malate | Malate/ Glucose | Pyruvate |
|---|---|---|---|---|---|---|---|---|
| SERD_L | 0 | 0 | 0 | 0 | 0 | 0 | 0 | 0 |
| SHSL1_1 | 0.00544 | 0.00472 | 0.00159 | 0.0041 | 0 | 0.00327 | 0 | 0 |
| SHSL2 | 0.0681 | 0.06133 | 0.02549 | 0.02588 | 0.04938 | 0.07041 | 0.09441 | 0 |
| SHSL4r | 0 | 0 | 0.00636 | 0 | 0 | 0.01306 | 0 | 0 |
| SUCCt2r | 0 | 0.35071 | 1.92519 | 3.35 | -0.80876 | -13.87046 | 0 | 3.35 |
| SUCD1 | 18.0321 | 19.59901 | 6.17237 | 10.12297 | 2.42713 | 0 | 0 | 7.12844 |
| SUCOAS | 17.95856 | 19.18225 | 4.21375 | 6.74298 | 3.18651 | 13.78372 | -0.09441 | 3.77844 |
| TALA | 0.71127 | 0.37804 | 0.4992 | -0.71418 | 0.40845 | -0.50775 | 0.39197 | -0.10577 |
| THRD | 0.16014 | 2.88958 | 0.6393 | 0.05052 | 0.11784 | 0.16792 | 0.22095 | 0.06935 |
| THRD_L | 0.21732 | 0.19524 | 0.12757 | 0.07847 | 0.14781 | 0.19425 | 0.27533 | 0.06454 |
| THRS | 0.48739 | 3.18357 | 0.84513 | 0.16998 | 0.34018 | 0.46837 | 0.63602 | 0.16556 |
| TKT1 | 0.71127 | 0.37804 | 0.4992 | -0.71418 | 0.40845 | -0.50775 | 0.39197 | -0.10577 |
| TKT2 | 0.50986 | 0.3779 | 0.4992 | -0.78928 | 0.40845 | -0.57501 | 0.38966 | -0.10577 |
| TPI | 11.85715 | 11.86571 | 5.38996 | 4.64367 | 10.88256 | 4.01063 | 7.70429 | 0 |
| TRPAS1 | 0 | 0 | 0 | 0 | 0 | 0 | 0 | 0 |
| TRPS1 | 0.03206 | 0 | 0 | 0.01195 | 0 | 0 | 0 | 0 |
| TYRTA | -0.06539 | 0 | 0 | -0.02438 | 0 | 0 | 0 | 0 |
| UNK5 | 0 | 0 | 0 | 0 | 0 | 0 | 0 | 0 |

**Table S8:** Predicted fluxes from ll-COBRA.

| Reaction | Glucose | Fructose | Gluconate | Glutamate/ Succinate | Glycerol | Malate | Malate/ Glucose | Pyruvate |
|---|---|---|---|---|---|---|---|---|
| 2S6HCCi | 0.00016 | 0.00014 | 0.00011 | 6e-05 | 0.00011 | 0.00015 | 2e-04 | 5e-05 |
| ACKr | -0.28438 | -0.25531 | -2.61004 | -0.10555 | 2.28961 | 9.90641 | 9.17438 | -0.07261 |
| ACONT | 17.24565 | 16.28515 | 2.59554 | 8.94193 | 1.83771 | 13.96049 | 3.42769 | 7.63315 |
| ACOTA | -0.11388 | 0 | 0 | -0.04246 | 0 | 0 | 0 | 0 |
| AKGDH | 16.71143 | 16.23303 | 4.54396 | 12.23272 | 3.23589 | 13.87046 | 0 | 3.40282 |
| AKGt2r | 0 | 0 | 0 | 0 | 0 | 0 | 0 | 0 |
| ALCD19y | 2.40937 | 4.72214 | -0.54423 | -0.36273 | 0 | -0.82612 | -0.63553 | -0.48972 |
| ALDD31_1 | 0 | 0 | 0 | 0 | 0 | 0 | 0 | 0 |
| ARGSL | 0.11388 | 0 | 0 | 0.04246 | 0 | 0 | 0 | 0 |
| ARGabc | 0 | 0 | 0 | 0 | 0 | 0 | 0 | 0 |
| ARGt2r | 0 | 0 | 0 | 0 | 0 | 0 | 0 | 0 |
| ASPO1 | 29.66541 | 24.31364 | 0 | 21.47431 | 0.08301 | 20.09286 | 0 | 2.40756 |
| ASPT | 0 | 0 | 0.02907 | 0 | 0 | 0 | 0 | 0 |
| ASPTA | -30.84904 | -26.06101 | -1.24814 | -21.91566 | -0.76181 | -21.07003 | -1.28574 | -2.69899 |
| CDPDSP_BS | 0.03304 | 0.02968 | 0.02352 | 0.01232 | 0.0224 | 0.03192 | 0.042 | 0.00952 |
| CITt10 | 0.06001 | 0.00654 | 0 | 0 | 0 | 0.05798 | 0.07629 | 0 |
| CITt14 | 0.00189 | 0.0017 | 0.00135 | 0.00071 | 0.00128 | 0.00183 | 0.0024 | 0.00054 |
| CITt15 | 0.01362 | 0.08378 | 0 | 0.08493 | 0 | 0.01187 | 0 | 0 |
| CITt2r | -0.07959 | -0.09567 | -2.29043 | -0.08716 | -3.57765 | -0.07236 | -7.01098 | -0.00172 |
| CLPNS2_BS | 0.00029 | 0.00026 | 0.00021 | 0.00011 | 2e-04 | 0.00028 | 0.00038 | 8e-05 |
| CS | 17.24565 | 16.28515 | 4.88187 | 8.94193 | 5.41408 | 13.96049 | 10.35753 | 7.63315 |
| CYSS_2 | 0.03239 | 0.02894 | 0 | 0.01158 | 0.01875 | 0.02667 | 0.03333 | 0 |
| CYSTGL_1 | 0.00668 | 0.00598 | 0.02553 | 0.00506 | 0.00405 | 0.00909 | 0.00941 | 0 |
| CYSTS_2 | 0.00124 | 0.00126 | 0.02394 | 0.00095 | 0.00405 | 0.00582 | 0.00941 | 0 |

| Reaction | Glucose | Fructose | Gluconate | Glutamate/ Succinate | Glycerol | Malate | Malate/ Glucose | Pyruvate |
|---|---|---|---|---|---|---|---|---|
| ENO | 21.45335 | 19.0492 | 9.982 | 12.43674 | 16.02664 | 10.53099 | 16.5552 | 0.06956 |
| FRUK | 0 | 0.99786 | 0.54423 | 2.70416 | 0.78677 | 1.31452 | 1.05034 | 0.40919 |
| FBA | 9.36225 | 8.68986 | 4.345 | 8.70341 | 4.8981 | 7.34887 | 8.79482 | 0.10609 |
| FBA2 | 2.40937 | 4.72214 | -0.54423 | -0.36273 | 0 | -0.82612 | -0.63553 | -0.37382 |
| FBP | 0 | 0 | 0 | 0 | 0 | 0 | 0 | 0.3031 |
| FEDCabc | 0.00204 | 0.00183 | 0.00138 | 0.00076 | 0 | 0.00034 | 0.00123 | 0.00059 |
| FRUpts | 2.40937 | 5.72 | 0 | 2.34143 | 0.78677 | 0.48841 | 0.41481 | 0.03536 |
| FUM | 16.82531 | 16.58374 | 6.13001 | 0.06045 | 2.42713 | -0.13084 | -0.1339 | 6.71712 |
| FUMt2r | 0 | 0 | -0.36821 | 0 | 0 | -0.13084 | -0.1339 | -0.0357 |
| G6PDH2r | 0.03519 | 0 | 0 | 0 | 0 | 0 | 0.23321 | 0 |
| GAPD | 21.76039 | 18.86455 | 10.11839 | 12.56935 | 16.18848 | 10.78344 | 16.88737 | 0 |
| GAPDi_nadp | 0 | 0 | 0 | 0 | 0 | 0 | 0 | 0 |
| GHMT2r | 0.08075 | 0.06026 | 0.00182 | 0.04823 | 0.04548 | 0.06481 | 0.08527 | 6e-05 |
| GLCNt2ir | 2.01161 | 1.14314 | 5.13 | 0.8888 | 1.05032 | 1.38732 | 0.95337 | 0 |
| GLCpts | 8.69437 | 6.27277 | 0.59994 | 3.61337 | 1.76104 | 3.32104 | 7.63 | 0 |
| GLUDxi | 0 | 0 | 0 | 0 | 0 | 0 | 0 | 0 |
| GLUSy | 0.00104 | 0.00086 | 0 | 0 | 0 | 0.00062 | 0 | 0 |
| GLUt2r | 0.56656 | 1.17465 | 1.19307 | 3.50561 | 0.86529 | 0.60063 | 0 | 3.0638 |
| GLYCt | -1.96619 | -2.47656 | 2.388 | 3.75833 | 6.22 | 2.90501 | 0.63515 | 2.75541 |
| GLYK | 0.44347 | 2.24584 | 1.84398 | 3.39571 | 6.2202 | 2.07918 | 0 | 2.26578 |
| GLYO1 | 0 | 0.83878 | 0 | 0 | 0 | 0 | 0 | 0 |
| GNKr | 2.01161 | 1.14314 | 5.13 | 0.8888 | 1.05032 | 1.38732 | 0.95337 | 0 |
| HISTD | 0 | 0 | 0 | 0.01798 | 0 | 0 | 0 | 0 |
| HISt2r | 0.04823 | 0.04332 | 0.03433 | 0 | 0.0327 | 0.04659 | 0.0613 | 0.0139 |
| HSTPTr | 0 | 0 | 0 | 0.01798 | 0 | 0 | 0 | 0 |
| ICDHyr | 15.07754 | 14.43825 | 2.59554 | 8.94193 | 1.83771 | 12.10702 | -1.62242 | 0.4722 |
| ICITt10 | 0 | 0.04737 | 0.04272 | 0.02238 | 0.04069 | 0 | 0 | 0.01729 |
| ICITt2 | -2.16811 | -1.89427 | -0.04272 | -0.02238 | -0.04069 | -1.85347 | -5.05011 | -7.17824 |

| Reaction | Glucose | Fructose | Gluconate | Glutamate/ Succinate | Glycerol | Malate | Malate/ Glucose | Pyruvate |
|---|---|---|---|---|---|---|---|---|
| LCADi | 0 | 0 | 0 | 0 | 0 | 0 | 0 | 0 |
| LDH_L | -0.57424 | -2.85166 | -4.00767 | -1.42079 | -4.3736 | 0 | -7.20753 | -0.9188 |
| L_LACt2r | -0.57424 | -2.76667 | -4.00767 | -1.31866 | -4.32314 | 1.61909 | -5.97596 | -0.80129 |
| MALt10 | 0 | 0.08499 | 0 | 0.10212 | 0.05046 | 1.61909 | 1.23157 | 0.11751 |
| MALt2r | 0 | 1.3598 | 0 | 8.30439 | 0.80737 | 24.8812 | 19.70507 | 1.88022 |
| MALt4 | 0 | 0.00133 | 0 | 0.0016 | 0.00079 | 0.00971 | 0.01924 | 0.00184 |
| MDH | 16.82531 | 18.02986 | 6.13001 | 8.46856 | 3.28575 | 26.3788 | 15.8379 | 8.34077 |
| ME2 | 0 | 0 | 0 | 0 | 0 | 0.00036 | 4.98408 | 0.37592 |
| MCITL2 | 0 | 0 | 0 | 0 | 0 | 0 | 0 | 0 |
| OXGDC | 0 | 0 | 0 | 0 | 0 | 0 | 0 | 0 |
| PC | 1.60397 | 0.00266 | 0 | 0.91472 | 2.80713 | 0 | 0 | 0 |
| PDH | 18.24971 | 16.13576 | 0 | 9.33444 | 8.43395 | 24.86325 | 21.14455 | 7.82367 |
| PFK | 9.36225 | 7.692 | 3.80078 | 5.99926 | 4.11133 | 6.03435 | 7.74448 | 0 |
| PGCD | 0.30703 | 0.23473 | 0.14008 | 0.13261 | 0.17716 | 0.25245 | 0.33217 | 0.04634 |
| GND | 2.0468 | 1.14314 | 1.15252 | -2.15705 | 1.05032 | -1.58063 | 1.18658 | -0.31437 |
| PGI | 8.51072 | 7.10946 | 1.00459 | 5.29615 | 1.6604 | 5.70133 | 7.20808 | -0.04277 |
| PGK_1 | 21.76039 | 18.86455 | 10.11839 | 12.56935 | 16.18848 | 10.78344 | 16.88737 | 0 |
| PGM_1 | 21.45335 | 19.0492 | 9.982 | 12.43674 | 16.02664 | 10.53099 | 16.5552 | 0.06956 |
| PHETA1 | -0.1038 | 0 | 0 | -0.03871 | 0 | -0.06711 | -0.00211 | 0 |
| PPCK | 0 | 0 | 0 | 0 | 0 | 11.44115 | 4.19463 | 0.41619 |
| PPS | 0 | 0 | 0 | 0 | 0 | 0 | 0 | 0 |
| PRAGSr | 0 | 0 | 0 | 0 | 0 | 0 | 0 | 0 |
| PTAr | -0.28438 | -0.25531 | -2.61004 | -0.10555 | 2.28961 | 9.90641 | 9.17438 | -0.07261 |
| PYK | 9.88674 | 7.00219 | 9.51977 | 6.30935 | 13.56889 | 17.97014 | 12.62404 | 0.43988 |
| PYRt2 | 1.16235 | 1.17285 | 0 | 0.049 | 0.68914 | 4.31954 | 4.93258 | 8.26 |
| RPE | 1.22113 | 0.75584 | 0.76842 | -1.50346 | 0.8169 | -1.08275 | 0.78162 | -0.21155 |
| RPI | -0.82567 | -0.38731 | -0.3841 | 0.6536 | -0.23342 | 0.49788 | -0.40495 | 0.10283 |
| SERAT | 0.03239 | 0.02894 | 0 | 0.01158 | 0.01875 | 0.02667 | 0.03333 | 0 |

| Reaction | Glucose | Fructose | Gluconate | Glutamate/ Succinate | Glycerol | Malate | Malate/ Glucose | Pyruvate |
|---|---|---|---|---|---|---|---|---|
| SERD_L | 0 | 0 | 0 | 0 | 0 | 0 | 0 | 0 |
| SHSL1_1 | 0.00544 | 0.00472 | 0.00159 | 0.0041 | 0 | 0.00327 | 0 | 0 |
| SHSL2 | 0.0681 | 0.06133 | 0.02549 | 0.02602 | 0.04938 | 0.07041 | 0.09441 | 0 |
| SHSL4r | 0 | 0 | 0.00636 | 0 | 0 | 0.01306 | 0 | 0 |
| SUCCt2r | 0 | 0.35071 | 1.92519 | -12.23272 | -0.80876 | -13.87046 | 0 | 3.35 |
| SUCD1 | 16.71143 | 16.58374 | 6.46915 | 0 | 2.42713 | 0 | 0 | 6.75282 |
| SUCOAS | 16.63789 | 16.16698 | 4.51053 | 12.20259 | 3.18651 | 13.78372 | -0.09441 | 3.40282 |
| TALA | 0.71127 | 0.37799 | 0.38421 | -0.71418 | 0.40845 | -0.50775 | 0.39197 | -0.10577 |
| THRD | 0.16014 | 0.99491 | 0.66884 | 0.0416 | 0.11784 | 0.16792 | 0.22095 | 0.06935 |
| THRD_L | 0.21732 | 0.19524 | 0.12757 | 0.07847 | 0.14781 | 0.19425 | 0.27533 | 0.06454 |
| THRS | 0.48739 | 1.2889 | 0.87466 | 0.16105 | 0.34018 | 0.46837 | 0.63602 | 0.16556 |
| TKT1 | 0.71127 | 0.37799 | 0.38421 | -0.71418 | 0.40845 | -0.50775 | 0.39197 | -0.10577 |
| TKT2 | 0.50986 | 0.37785 | 0.38421 | -0.78928 | 0.40845 | -0.57501 | 0.38966 | -0.10577 |
| TPI | 11.85715 | 9.79769 | 5.38996 | 4.64367 | 10.88256 | 4.01063 | 7.70429 | 0 |
| TRPAS1 | 0 | 0 | 0 | 0 | 0 | 0 | 0 | 0 |
| TRPS1 | 0.03206 | 0 | 0 | 0.01195 | 0 | 0 | 0 | 0 |
| TYRTA | -0.06539 | 0 | 0 | -0.02438 | 0 | 0 | 0 | 0 |
| UNK5 | 0 | 0 | 0 | 0 | 0 | 0 | 0 | 0 |

**Table S9:** Predicted fluxes from pFBA (one or two carbon sources allowed according to each specific condition)

| Reaction | Glucose | Fructose | Gluconate | Glutamate/ Succinate | Glycerol | Malate | Malate/ Glucose | Pyruvate |
|---|---|---|---|---|---|---|---|---|
| 2S6HCCi | 0 | 0 | 0 | 0 | 0 | 0 | 0 | 0 |
| ACKr | 1.98036 | 1.98036 | 1.88348 | 1.376 | 1.83448 | 2.07579 | 2.07579 | 3.27273 |
| ACONT | 0 | 0 | 0 | 0 | 0 | 0 | 0 | 0 |
| ACOTA | 0 | 0 | 0 | 0 | 0 | 0 | 0 | 0 |
| AKGDH | 0 | 0 | 0 | 0.73867 | 0 | 0 | 0 | 0 |
| AKGt2r | 0 | 0 | 0 | 0 | 0 | 0 | 0 | 0 |
| ALCD19y | 0 | 0 | 0 | 0 | 0 | 0 | 0 | 0 |
| ALDD31_1 | 0 | 0 | 0 | 0 | 0 | 0 | 0 | 0 |
| ARGSL | 0 | 0 | 0 | 0 | 0 | 0 | 0 | 0 |
| ARGabc | 0 | 0 | 0 | 0 | 0 | 0 | 0 | 0 |
| ARGt2r | 0 | 0 | 0 | 0 | 0 | 0 | 0 | 0 |
| ASPO1 | 0 | 0 | 0 | 0 | 0 | 0 | 0 | 0 |
| ASPT | 0 | 0 | 0 | 0 | 0 | 0 | 0 | 0 |
| ASPTA | 0 | 0 | 0 | 0 | 0 | 0 | 0 | 0 |
| CDPDSP_BS | 0 | 0 | 0 | 0 | 0 | 0 | 0 | 0 |
| CITt10 | 0 | 0 | 0 | 0 | 0 | 0 | 0 | 0 |
| CITt14 | 0 | 0 | 0 | 0 | 0 | 0 | 0 | 0 |
| CITt15 | 0 | 0 | 0 | 0 | 0 | 0 | 0 | 0 |
| CITt2r | 0 | 0 | 0 | 0 | 0 | 0 | 0 | 0 |
| CLPNS2_BS | 0 | 0 | 0 | 0 | 0 | 0 | 0 | 0 |
| CS | 0 | 0 | 0 | 0 | 0 | 0 | 0 | 0 |
| CYSS_2 | 0 | 0 | 0 | 0 | 0 | 0 | 0 | 0 |
| CYSTGL_1 | 0 | 0 | 0 | 0 | 0 | 0 | 0 | 0 |
| CYSTS_2 | 0 | 0 | 0 | 0 | 0 | 0 | 0 | 0 |

| Reaction | Glucose | Fructose | Gluconate | Glutamate/ Succinate | Glycerol | Malate | Malate/ Glucose | Pyruvate |
|---|---|---|---|---|---|---|---|---|
| ENO | 1.29236 | 1.29236 | 1.19548 | 0.688 | 1.14648 | 0.688 | 0.688 | 0 |
| FRUK | 0 | 0.30218 | 0 | 0 | 0 | 0 | 0 | 0 |
| FBA | 0.30218 | 0.30218 | 0.20299 | 0 | 0 | 0 | 0 | 0 |
| FBA2 | 0 | 0 | 0 | 0 | 0 | 0 | 0 | 0 |
| FBP | 0 | 0 | 0 | 0 | 0 | 0 | 0 | 0 |
| FEDCabc | 0 | 0 | 0 | 0 | 0 | 0 | 0 | 0 |
| FRUpts | 0 | 0.30218 | 0 | 0 | 0 | 0 | 0 | 0 |
| FUM | 0 | 0 | 0 | 0 | 0 | 0 | 0 | 0 |
| FUMt2r | 0 | 0 | 0 | 0 | 0 | 0 | 0 | 0 |
| G6PDH2r | 0 | 0 | 0 | 0 | 0 | 0 | 0 | 0 |
| GAPD | 1.29236 | 1.29236 | 1.49997 | 0.688 | 1.14648 | 0.688 | 1.38779 | 0 |
| GAPDi_nadp | 0 | 0 | 0.30449 | 0 | 0 | 0 | 0.69979 | 0 |
| GHMT2r | 0 | 0 | 0 | 0 | 0 | 0 | 0 | 0 |
| GLCNt2ir | 0 | 0 | 0.30449 | 0 | 0 | 0 | 0 | 0 |
| GLCpts | 0.30218 | 0 | 0 | 0 | 0 | 0 | 0 | 0 |
| GLUDxi | 0 | 0 | 0 | 0.73867 | 0 | 0 | 0 | 0 |
| GLUSy | 0 | 0 | 0 | 0 | 0 | 0 | 0 | 0 |
| GLUt2r | 0 | 0 | 0 | 0.73867 | 0 | 0 | 0 | 0 |
| GLYCt | 0 | 0 | 0 | 0 | 0.45848 | 0 | 0 | 0 |
| GLYK | 0 | 0 | 0 | 0 | 0.45848 | 0 | 0 | 0 |
| GLYO1 | 0 | 0 | 0 | 0 | 0 | 0 | 0 | 0 |
| GNKr | 0 | 0 | 0.30449 | 0 | 0 | 0 | 0 | 0 |
| HISTD | 0 | 0 | 0 | 0 | 0 | 0 | 0 | 0 |
| HISt2r | 0 | 0 | 0 | 0 | 0 | 0 | 0 | 0 |
| HSTPTr | 0 | 0 | 0 | 0 | 0 | 0 | 0 | 0 |
| ICDHyr | 0 | 0 | 0 | 0 | 0 | 0 | 0 | 0 |
| ICITt10 | 0 | 0 | 0 | 0 | 0 | 0 | 0 | 0 |
| ICITt2 | 0 | 0 | 0 | 0 | 0 | 0 | 0 | 0 |

| Reaction | Glucose | Fructose | Gluconate | Glutamate/ Succinate | Glycerol | Malate | Malate/ Glucose | Pyruvate |
|---|---|---|---|---|---|---|---|---|
| LCADi | 0 | 0 | 0 | 0 | 0 | 0 | 0 | 0 |
| LDH_L | 0 | 0 | 0 | 0 | 0 | 0 | 0 | 0 |
| L_LACt2r | 0 | 0 | 0 | 0 | 0 | 0 | 0 | 0 |
| MALt10 | 0 | 0 | 0 | 0 | 0 | 0 | 0 | 0 |
| MALt2r | 0 | 0 | 0 | 0 | 0 | 0.69979 | 0.69979 | 0 |
| MALt4 | 0 | 0 | 0 | 0 | 0 | 0 | 0 | 0 |
| MDH | 0 | 0 | 0 | 0 | 0 | 0.69979 | 0 | 0 |
| ME2 | 0 | 0 | 0 | 0 | 0 | 0 | 0.69979 | 0 |
| MCITL2 | 0 | 0 | 0 | 0 | 0 | 0 | 0 | 0 |
| OXGDC | 0 | 0 | 0 | 0 | 0 | 0 | 0 | 0 |
| PC | 0 | 0 | 0 | 0 | 0 | 0 | 0 | 0 |
| PDH | 1.98036 | 1.98036 | 1.88348 | 1.376 | 1.83448 | 2.07579 | 2.07579 | 3.27273 |
| PFK | 0.30218 | 0 | 0.20299 | 0 | 0 | 0 | 0 | 0 |
| PGCD | 0 | 0 | 0 | 0 | 0 | 0 | 0 | 0 |
| GND | 0 | 0 | 0.30449 | 0 | 0 | 0 | 0 | 0 |
| PGI | 0.30218 | 0 | 0 | 0 | 0 | 0 | 0 | 0 |
| PGK_1 | 1.29236 | 1.29236 | 1.19548 | 0.688 | 1.14648 | 0.688 | 0.688 | 0 |
| PGM_1 | 1.29236 | 1.29236 | 1.19548 | 0.688 | 1.14648 | 0.688 | 0.688 | 0 |
| PHETA1 | 0 | 0 | 0 | 0 | 0 | 0 | 0 | 0 |
| PPCK | 0 | 0 | 0 | 0 | 0 | 0.69979 | 0 | 0 |
| PPS | 0 | 0 | 0 | 0 | 0 | 0 | 0 | 0 |
| PRAGSr | 0 | 0 | 0 | 0 | 0 | 0 | 0 | 0 |
| PTAr | 1.98036 | 1.98036 | 1.88348 | 1.376 | 1.83448 | 2.07579 | 2.07579 | 3.27273 |
| PYK | 0.99018 | 0.99018 | 1.19548 | 0.688 | 1.14648 | 1.38779 | 0.688 | 0 |
| PYRt2 | 0 | 0 | 0 | 0 | 0 | 0 | 0 | 3.27273 |
| RPE | 0 | 0 | 0.20299 | 0 | 0 | 0 | 0 | 0 |
| RPI | 0 | 0 | 0.1015 | 0 | 0 | 0 | 0 | 0 |
| SERAT | 0 | 0 | 0 | 0 | 0 | 0 | 0 | 0 |

| Reaction | Glucose | Fructose | Gluconate | Glutamate/ Succinate | Glycerol | Malate | Malate/ Glucose | Pyruvate |
|---|---|---|---|---|---|---|---|---|
| SERD_L | 0 | 0 | 0 | 0 | 0 | 0 | 0 | 0 |
| SHSL1_1 | 0 | 0 | 0 | 0 | 0 | 0 | 0 | 0 |
| SHSL2 | 0 | 0 | 0 | 0 | 0 | 0 | 0 | 0 |
| SHSL4r | 0 | 0 | 0 | 0 | 0 | 0 | 0 | 0 |
| SUCCt2r | 0 | 0 | 0 | 0.73867 | 0 | 0 | 0 | 0 |
| SUCD1 | 0 | 0 | 0 | 0 | 0 | 0 | 0 | 0 |
| SUCOAS | 0 | 0 | 0 | 0.73867 | 0 | 0 | 0 | 0 |
| TALA | 0 | 0 | 0.1015 | 0 | 0 | 0 | 0 | 0 |
| THRD | 0 | 0 | 0 | 0 | 0 | 0 | 0 | 0 |
| THRD_L | 0 | 0 | 0 | 0 | 0 | 0 | 0 | 0 |
| THRS | 0 | 0 | 0 | 0 | 0 | 0 | 0 | 0 |
| TKT1 | 0 | 0 | 0.1015 | 0 | 0 | 0 | 0 | 0 |
| TKT2 | 0 | 0 | 0.1015 | 0 | 0 | 0 | 0 | 0 |
| TPI | 0.30218 | 0.30218 | 0.20299 | 0 | 0.45848 | 0 | 0 | 0 |
| TRPAS1 | 0 | 0 | 0 | 0 | 0 | 0 | 0 | 0 |
| TRPS1 | 0 | 0 | 0 | 0 | 0 | 0 | 0 | 0 |
| TYRTA | 0 | 0 | 0 | 0 | 0 | 0 | 0 | 0 |
| UNK5 | 0 | 0 | 0 | 0 | 0 | 0 | 0 | 0 |

**Table S10:** List of Pearson's correlation coefficients (r) between pFBA flux prediction results (one or two carbon sources allowed according to each specific condition) and $^{13}C$ metabolic flux data.

| Reaction | r | P-value (BH method adjusted) |
|---|---|---|
| Glucose | 0.76 | 0.03 |
| Fructose | 1 | 0 |
| Gluconate | 1 | 0 |
| Glycerol | 1 | 0 |
| Malate | 0.95 | 0 |
| Pyruvate | 1 | 0 |
| Glutamate | 1 | 0 |
| Succinate | 1 | 0 |
| PGI | 0.73 | 0.03 |
| PFK | 0.77 | 0.02 |
| FBP | NA | NA |
| FBA | 0.8 | 0.02 |
| TPI | 0.81 | 0.02 |
| GAPD | 0.8 | 0.02 |
| GAPDi_nadp | -0.32 | 0.78 |
| PGK_1 | 0.72 | 0.03 |
| PYK | 0.4 | 0.2 |
| G6PDH2r | NA | NA |
| GND | 0.76 | 0.02 |
| RPE | 0.82 | 0.02 |
| RPI | 0.64 | 0.06 |
| TKT1 | 0.78 | 0.02 |
| TKT2 | 0.86 | 0.01 |
| TALA | 0.78 | 0.02 |
| PDH | 0.25 | 0.31 |
| CS | NA | NA |
| ICDHyr | NA | NA |
| AKGDH | 0.51 | 0.12 |
| SUCD1 | NA | NA |
| MDH | 0.78 | 0.02 |
| PPCK | 0.8 | 0.02 |
| ME2 | -0.01 | 0.52 |
| PC | NA | NA |
| PTAr | 0.14 | 0.39 |
| PGM_1 | 0.72 | 0.03 |
| ENO | 0.72 | 0.03 |
| ACONT | NA | NA |
| FUM | NA | NA |
| ACKr | 0.14 | 0.39 |
| SUCOAS | 0.51 | 0.12 |

**Table S11:** Predicted fluxes from iMAT

| Reaction | Glucose | Fructose | Gluconate | Glutamate/ Succinate | Glycerol | Malate | Malate/ Glucose | Pyruvate |
|---|---|---|---|---|---|---|---|---|
| 2S6HCCi | 0 | 0 | 0 | 0 | 0 | 0 | 0 | 0 |
| ACKr | 1000 | 1000 | 0 | 1000 | 0 | 1 | -1 | 0 |
| ACONT | 1 | 1.10429 | 1 | 173.77375 | 31 | 237.47 | 1 | 1 |
| ACOTA | -1 | -1 | 0 | -1 | 0 | -273.22 | -1 | -1 |
| AKGDH | 5 | 7.79786 | 2 | 176.98375 | 34.21 | 238.47 | 4 | 1 |
| AKGt2r | 0 | 0 | 0 | 0 | 0 | 0 | 0 | 0 |
| ALCD19y | 0 | 0 | 0 | 0 | -1 | 0 | 0 | 0 |
| ALDD31_1 | 0 | 0 | 0 | 0 | 0 | 0 | 0 | 0 |
| ARGSL | 1 | 1 | 1 | 1 | 217.15536 | 1 | 1 | 1 |
| ARGabc | 0 | 0 | 0 | 0 | 0 | 0 | 0 | 0 |
| ARGt2r | 0 | 0 | 0 | 0 | 0 | 0 | 0 | 0 |
| ASPO1 | 642.15459 | 1 | 1 | 589.82937 | 0 | 149.496 | 504.33944 | 1 |
| ASPT | 0 | 0 | 0 | 0 | 0 | 0 | 0 | 0 |
| ASPTA | -988.42 | -11.94214 | -4 | -596.82937 | -224.15536 | -154.496 | -706.47075 | -5 |
| CDPDSP_BS | 0 | 0 | 0 | 0 | 0 | 0 | 0 | 0 |
| CITt10 | 0 | 0 | 0 | 0 | 0 | 0 | 0 | 0 |
| CITt14 | 0 | 0 | 0 | 0 | 0 | 0 | 0 | 0 |
| CITt15 | 0 | 0 | 0 | 0 | 0 | 0 | 0 | 0 |
| CITt2r | 0 | 0 | 0 | 0 | 0 | 0 | 0 | 0 |
| CLPNS2_BS | 0 | 0 | 0 | 0 | 0 | 0 | 0 | 0 |
| CS | 1 | 1.10429 | 1 | 173.77375 | 31 | 237.47 | 1 | 1 |
| CYSS_2 | 4 | 1 | 0 | 1 | 0 | 1 | 1 | 1 |
| CYSTGL_1 | 0 | 0 | 0 | 0 | 0 | 0 | 0 | 0 |
| CYSTS_2 | 0 | 0 | 0 | 0 | 0 | 0 | 0 | 0 |

| Reaction | Glucose | Fructose | Gluconate | Glutamate/ Succinate | Glycerol | Malate | Malate/ Glucose | Pyruvate |
|---|---|---|---|---|---|---|---|---|
| ENO | 221.43767 | 195.05361 | 26.52 | 210.11688 | 144.06681 | 397.748 | 471.805 | 1 |
| FRUK | 0 | 4.72 | 0 | 0 | 0 | 0 | 0 | 0 |
| FBA | 1 | 5.72 | 1 | 1 | 1 | 1 | 2.315 | 1 |
| FBA2 | 0 | 1 | 0 | 1 | 1 | 0 | 0 | 5.72 |
| FBP | 0 | 0 | 0 | 0 | 0 | 0 | 0 | 0 |
| FEDCabc | 0 | 0 | 0 | 0 | 0 | 0 | 0 | 0 |
| FRUpts | 0 | 5.72 | 0 | 1 | 1 | 0 | 0 | 5.72 |
| FUM | 344.26541 | 14.74 | 5 | 183.33375 | 253.36536 | 244.82 | 199.13131 | 4 |
| FUMt2r | 0 | 0 | 0 | 0 | 0 | 0 | 0 | 0 |
| G6PDH2r | 8.63 | 6.63 | 1 | 3.63 | 2 | 11.26 | 8.63 | 1 |
| GAPD | 229.43767 | 202.99576 | 136.78279 | 323.04875 | 145.56681 | 408.748 | 977.305 | 128.74583 |
| GAPDi_nadp | 0 | 0 | 0 | 0 | 0 | 1 | 0 | 59.23741 |
| GHMT2r | 7.79 | 7.94214 | 110.26279 | 112.56187 | 7 | 4 | 999 | 73.22842 |
| GLCNt2ir | 0 | 0 | 5.13 | 0 | 0 | 0 | 0 | 0 |
| GLCpts | 7.63 | 1 | 0 | 0 | 0 | 0 | 1 | 0 |
| GLUDxi | 1000 | 1 | 884.73721 | 1 | 1 | 1000 | 1 | 1 |
| GLUSy | 1 | 1 | 1 | 263.52563 | 1 | 1 | 1 | 1 |
| GLUt2r | 0 | 0 | 0 | 0 | 0 | 0 | 0 | 0 |
| GLYCt | 0 | 0 | 0 | 0 | 2 | 0 | 0 | 0 |
| GLYK | 0 | 0 | 0 | 0 | 1 | 0 | 0 | 0 |
| GLYO1 | 6.58 | 6.94214 | 106.26279 | 111.56187 | 6 | 1 | 998 | 73.22842 |
| GNKr | 0 | 0 | 5.13 | 0 | 0 | 0 | 0 | 0 |
| HISTD | 1.79 | 4.94214 | 1 | 1 | 1 | 1 | 1 | 1 |
| HISt2r | 0 | 0 | 0 | 0 | 0 | 0 | 0 | 0 |
| HSTPTr | 1.79 | 4.94214 | 1 | 1 | 1 | 1 | 1 | 1 |
| ICDHyr | 1 | 1.10429 | 1 | 173.77375 | 31 | 237.47 | 1 | 1 |
| ICITt10 | 0 | 0 | 0 | 0 | 0 | 0 | 0 | 0 |
| ICITt2 | 0 | 0 | 0 | 0 | 0 | 0 | 0 | 0 |

| Reaction | Glucose | Fructose | Gluconate | Glutamate/ Succinate | Glycerol | Malate | Malate/ Glucose | Pyruvate |
|---|---|---|---|---|---|---|---|---|
| LCADi | 0 | 0 | 0 | 0 | 0 | 0 | 0 | 0 |
| LDH_L | 0 | 0 | 0 | 0 | -1 | -344.156 | -1000 | -1 |
| L_LACt2r | 0 | 0 | 0 | 0 | -1 | -343.156 | -975.49 | -1 |
| MALt10 | 0 | 0 | 0 | 0 | 0 | 1 | 24.51 | 0 |
| MALt2r | 0 | 0 | 0 | 0 | 0 | 24.51 | 1 | 0 |
| MALt4 | 0 | 0 | 0 | 0 | 0 | 1 | 1 | 0 |
| MDH | -1 | 11.04643 | 3 | 180.77375 | 253.36536 | 242.47 | -1 | 1 |
| ME2 | 345.26541 | 3.69357 | 2 | 2.56 | 0 | 28.86 | 226.64131 | 3 |
| MCITL2 | 0 | 0 | 0 | 0 | 0 | 0 | 0 | 1 |
| OXGDC | 0 | 0 | 0 | 0 | 0 | 0 | 0 | 0 |
| PC | 348.26541 | 1 | 1 | 1 | 1.79 | 1 | 204.13131 | 24.70204 |
| PDH | 977.63 | 391.79366 | 1 | 526.72562 | 283.84362 | 1 | 456.36 | 49.8004 |
| PFK | 1 | 1 | 1 | 1 | 1 | 1 | 2.315 | 1 |
| PGCD | 11.79 | 8.94214 | 110.26279 | 113.56187 | 7 | 5 | 1000 | 74.22842 |
| GND | 6.79 | 6.63 | 6.13 | -1 | 2 | 11.26 | 8.63 | 1 |
| PGI | -1 | 1 | -1 | 4 | 1 | -11.26 | -1 | -1 |
| PGK_1 | 229.43767 | 202.99576 | 136.78279 | 323.04875 | 145.56681 | 407.748 | 977.305 | 69.50842 |
| PGM_1 | 221.43767 | 195.05361 | 26.52 | 210.11688 | 144.06681 | 397.748 | 471.805 | 1 |
| PHETA1 | -1 | -1 | -1 | -1 | -1 | -1 | -1 | -1 |
| PPCK | 0 | 0 | 0 | 1 | 0 | 1 | 0 | 19.70204 |
| PPS | 0 | 0 | 0 | 0 | 0 | 0 | 0 | 0 |
| PRAGSr | 1 | 1 | 1 | 1 | 1 | 1 | 1 | 0 |
| PTAr | 1000 | 1000 | 0 | 1000 | 0 | 1 | -1 | 0 |
| PYK | 205.80767 | 183.41647 | 1 | 200.11688 | 139.06681 | 385.488 | 460.175 | 1 |
| PYRt2 | 0 | 0 | 0 | 0 | 0 | 0 | 0 | 0 |
| RPE | 2 | 0 | 0 | -3 | 0 | 4.63 | 3.315 | -2.99701 |
| RPI | -4.79 | -6.63 | -6.13 | -2 | -2 | -6.63 | -5.315 | -3.99701 |
| SERAT | 4 | 1 | 0 | 1 | 0 | 1 | 1 | 1 |

| Reaction | Glucose | Fructose | Gluconate | Glutamate/ Succinate | Glycerol | Malate | Malate/ Glucose | Pyruvate |
|---|---|---|---|---|---|---|---|---|
| SERD_L | 0 | 0 | 0 | 0 | 0 | 0 | 0 | 0 |
| SHSL1_1 | 4 | 1 | 0 | 1 | 0 | 1 | 1 | 1 |
| SHSL2 | 1 | 1 | 0 | 1 | 5 | 0 | 4 | 0 |
| SHSL4r | 1 | 1 | 0 | 1 | 0 | 0 | 1 | 0 |
| SUCCt2r | 0 | 0 | 0 | 3.35 | 0 | 3.35 | 0 | 0 |
| SUCD1 | 5 | 7.79786 | 2 | 180.33375 | 34.21 | 241.82 | 4 | 2 |
| SUCOAS | -1 | 4.79786 | 221.4018 | 173.98375 | 1 | 237.47 | -1 | 1 |
| TALA | 3 | 1.22929 | 5.13 | 1 | 1 | 5.63 | 4.315 | 1.99701 |
| THRD | 0 | 0 | 0 | 0 | 0 | 0 | 0 | 0 |
| THRD_L | 1 | 1 | 0 | 1 | 0 | 1 | 1 | 1 |
| THRS | 1 | 1 | 0 | 1 | 0 | 1 | 1 | 1 |
| TKT1 | 3 | 1.22929 | 5.13 | 1 | 1 | 5.63 | 4.315 | 1.99701 |
| TKT2 | -1 | -1.22929 | -5.13 | -4 | -1 | -1 | -1 | -4.99401 |
| TPI | -535.75467 | -1 | -735.69443 | -1 | -1 | 1 | 2.315 | 6.72 |
| TRPAS1 | 0 | 0 | 0 | 0 | 0 | 0 | 0 | 0 |
| TRPS1 | 0 | 0 | 0 | 0 | 0 | 0 | 0 | 0 |
| TYRTA | -1 | -1 | -1 | -1 | -1 | -1 | -1 | -1 |
| UNK5 | 0 | 0 | 0 | 0 | 0 | 0 | 0 | 0 |

**Table S12:** Predicted fluxes from mCADRE.

| Reaction | Glucose | Fructose | Gluconate | Glutamate/ Succinate | Glycerol | Malate | Malate/ Glucose | Pyruvate |
|---|---|---|---|---|---|---|---|---|
| 2S6HCCi | 0.00016 | 0.00014 | 0 | 0 | 0.00011 | 0.00015 | 2e-04 | 0 |
| ACKr | -1000 | -1000 | -2.57143 | 0 | -1000 | -1000 | -1000 | -2.3128 |
| ACONT | 29.26714 | 28.94899 | 1.71429 | 0 | 28.25965 | 29.16109 | 29.58686 | 1.3216 |
| ACOTA | -0.11388 | -0.1023 | 0 | 0 | -0.07721 | -0.11002 | -0.14477 | 0 |
| AKGDH | 0 | 0 | 1.71429 | 0 | 0 | 0 | 0 | 1.3216 |
| AKGt2r | 0 | 0 | 0 | 0 | 0 | 0 | 0 | 0 |
| ALCD19y | 5.72 | 5.72 | 0 | 0 | 5.72 | 5.72 | 5.72 | -3.304 |
| ALDD31_1 | 0 | 0 | 0 | 0 | 0 | 0 | 0 | 0 |
| ARGSL | 0.11388 | 0.1023 | 0 | 0 | 0.07721 | 0.11002 | 0.14477 | 0 |
| ARGabc | 0 | 0 | 0 | 0 | 0 | 0 | 0 | 0 |
| ARGt2r | 0 | 0 | 0 | 0 | 0 | 0 | 0 | 0 |
| ASPO1 | 0 | 0 | 0 | 0 | 0 | 0 | 0 | 0 |
| ASPT | 0 | 0 | 0 | 0 | 0 | 0 | 0 | 0 |
| ASPTA | -1.23879 | -1.11281 | 0 | 0 | -0.83986 | -1.1968 | -1.57473 | 0 |
| CDPDSP_BS | 0.03304 | 0.02968 | 0 | 0 | 0.0224 | 0.03192 | 0.042 | 0 |
| CITt10 | 0 | 0 | 0 | 0 | 0 | 0 | 0 | 0 |
| CITt14 | 0 | 0 | 0 | 0 | 0 | 0 | 0 | 0 |
| CITt15 | 1000 | 1000 | 0 | 0 | 1000 | 1000 | 1000 | 0 |
| CITt2r | -1000 | -1000 | 0 | 0 | -1000 | -1000 | -1000 | 0 |
| CLPNS2_BS | 3e-04 | 0.00026 | 0 | 0 | 2e-04 | 0.00028 | 0.00038 | 0 |
| CS | 29.26714 | 28.94899 | 1.71429 | 0 | 28.25965 | 29.16109 | 29.58686 | 1.3216 |
| CYSS_2 | 0 | 0 | 2.57143 | 0 | 0 | 0 | 0 | 2.3128 |
| CYSTGL_1 | 0.224 | 0.20122 | 0 | 0 | 0.15186 | 0.2164 | 0.28474 | 0 |
| CYSTS_2 | 0.03362 | 0.0302 | 0 | 0 | 0.0228 | 0.03248 | 0.04274 | 0 |

| Reaction | Glucose | Fructose | Gluconate | Glutamate/ Succinate | Glycerol | Malate | Malate/ Glucose | Pyruvate |
|---|---|---|---|---|---|---|---|---|
| ENO | 933.99331 | 934.14529 | 2.57143 | 0 | 934.47458 | 934.04397 | 933.6376 | 0.9912 |
| FRUK | 0 | 0 | 0 | 0 | 0 | 0 | 0 | 3.304 |
| FBA | 0 | 0 | 2.57143 | 0 | 0 | 0 | 0 | 3.304 |
| FBA2 | 5.72 | 5.72 | 0 | 0 | 5.72 | 5.72 | 5.72 | -3.304 |
| FBP | 0 | 0 | 0 | 0 | 0 | 0 | 0 | 0 |
| FEDCabc | 0 | 0 | 0 | 0 | 0 | 0 | 0 | 0 |
| FRUpts | 5.72 | 5.72 | 0 | 0 | 5.72 | 5.72 | 5.72 | 0 |
| FUM | 3.99594 | 3.5518 | 0 | 0 | 2.58951 | 3.84789 | 4.65159 | 1.3216 |
| FUMt2r | -28.37407 | -28.52518 | 0 | 0 | -28.85259 | -28.42444 | -28.97894 | 0 |
| G6PDH2r | 8.54196 | 8.71668 | 0 | 0 | 9.09523 | 8.6002 | 8.07605 | 0 |
| GAPD | 934.56796 | 934.6615 | 5.14286 | 0 | 934.86417 | 934.59914 | 934.36808 | 6.9384 |
| GAPDi_nadp | 0 | 0 | 0 | 0 | 0 | 0 | 0 | 3.6344 |
| GHMT2r | 0.34836 | 0.31293 | 0 | 0 | 0.23618 | 0.33655 | 0.44283 | 0 |
| GLCNt2ir | 5.13 | 5.13 | 0 | 0 | 5.13 | 5.13 | 5.13 | 0 |
| GLCpts | 0 | 0 | 2.57143 | 0 | 0 | 0 | 0 | 0 |
| GLUDxi | 0 | 0 | 0 | 0 | 0 | 0 | 0 | 0 |
| GLUSy | 0 | 0 | 2.57143 | 0 | 0 | 0 | 0 | 0 |
| GLUt2r | 0 | 0 | 0 | 0 | 0 | 0 | 0 | 0 |
| GLYCt | -5.72029 | -5.72027 | 0 | 0 | -5.7202 | -5.72028 | -5.72037 | 3.304 |
| GLYK | 0 | 0 | 0 | 0 | 0 | 0 | 0 | 0 |
| GLYO1 | 0 | 0 | 0 | 0 | 0 | 0 | 0 | 0 |
| GNKr | 5.13 | 5.13 | 0 | 0 | 5.13 | 5.13 | 5.13 | 0 |
| HISTD | 0.04823 | 0.04332 | 0 | 0 | 0.0327 | 0.04659 | 0.0613 | 0 |
| HISt2r | 0 | 0 | 0 | 0 | 0 | 0 | 0 | 0 |
| HSTPTr | 0.04823 | 0.04332 | 0 | 0 | 0.0327 | 0.04659 | 0.0613 | 0 |
| ICDHyr | 29.26714 | 28.94899 | 1.71429 | 0 | 28.25965 | 29.16109 | 29.58686 | 1.3216 |
| ICITt10 | 0 | 0 | 0 | 0 | 0 | 0 | 0 | 0 |
| ICITt2 | 0 | 0 | 0 | 0 | 0 | 0 | 0 | 0 |

| Reaction | Glucose | Fructose | Gluconate | Glutamate/ Succinate | Glycerol | Malate | Malate/ Glucose | Pyruvate |
|---|---|---|---|---|---|---|---|---|
| LCADi | 0 | 0 | 0 | 0 | 0 | 0 | 0 | 0 |
| LDH_L | 0 | 0 | 0 | 0 | 0 | 0 | 0 | -10.2424 |
| L_LACt2r | 0 | 0 | 0 | 0 | 0 | 0 | 0 | -10.2424 |
| MALt10 | 0 | 0 | 0 | 0 | 0 | 0 | 0 | 0 |
| MALt2r | 26.51 | 26.51 | 0 | 0 | 26.51 | 26.51 | 26.51 | 0 |
| MALt4 | 0 | 0 | 0 | 0 | 0 | 0 | 0 | 0 |
| MDH | 30.50594 | 30.0618 | -2.57143 | 0 | 29.09951 | 30.35789 | 31.16159 | 1.3216 |
| ME2 | 0 | 0 | 2.57143 | 0 | 0 | 0 | 0 | 0 |
| MCITL2 | 0 | 0 | 0 | 0 | 0 | 0 | 0 | 0 |
| OXGDC | 28.69082 | 28.43127 | 0 | 0 | 27.86892 | 28.6043 | 29.86208 | 0 |
| PC | 0 | 0 | 4.28571 | 0 | 0 | 0 | 0 | 0 |
| PDH | 30.40757 | 29.97344 | 0 | 0 | 29.03282 | 30.26286 | 31.03656 | 1.3216 |
| PFK | 0 | 0 | 2.57143 | 0 | 0 | 0 | 0 | 0 |
| PGCD | 0.57465 | 0.51621 | 2.57143 | 0 | 0.38959 | 0.55517 | 0.73048 | 2.3128 |
| GND | 13.67196 | 13.84668 | 0 | 0 | 14.22523 | 13.7302 | 13.20605 | 0 |
| PGI | -8.69041 | -8.85003 | 2.57143 | 0 | -9.19587 | -8.74362 | -8.26476 | 0 |
| PGK_1 | 934.56796 | 934.6615 | 5.14286 | 0 | 934.86417 | 934.59914 | 934.36808 | 3.304 |
| PGM_1 | 933.99331 | 934.14529 | 2.57143 | 0 | 934.47458 | 934.04397 | 933.6376 | 0.9912 |
| PHETA1 | -0.1038 | -0.09325 | 0 | 0 | -0.07038 | -0.10029 | -0.13195 | 0 |
| PPCK | 0 | 0 | 0 | 0 | 0 | 0 | 0 | 0 |
| PPS | 0 | 0 | 0 | 0 | 0 | 0 | 0 | 0 |
| PRAGSr | 0.10747 | 0.09654 | 0 | 0 | 0.07286 | 0.10383 | 0.13661 | 0 |
| PTAr | -1000 | -1000 | -2.57143 | 0 | -1000 | -1000 | -1000 | -2.3128 |
| PYK | 927.81 | 928.00909 | 0 | 0 | 928.44047 | 927.87636 | 927.32863 | 0.9912 |
| PYRt2 | -1000 | -1000 | 0 | 0 | -1000 | -1000 | -1000 | 8.26 |
| RPE | 8.88333 | 9.02333 | 0 | 0 | 9.32666 | 8.92999 | 8.50999 | 0 |
| RPI | -4.78863 | -4.82335 | 0 | 0 | -4.89856 | -4.8002 | -4.69606 | 0 |
| SERAT | 0 | 0 | 2.57143 | 0 | 0 | 0 | 0 | 2.3128 |

| Reaction | Glucose | Fructose | Gluconate | Glutamate/ Succinate | Glycerol | Malate | Malate/ Glucose | Pyruvate |
|---|---|---|---|---|---|---|---|---|
| SERD_L | 0 | 0 | 0 | 0 | 0 | 0 | 0 | 0 |
| SHSL1_1 | 0.19037 | 0.17101 | 0 | 0 | 0.12907 | 0.18392 | 0.242 | 0 |
| SHSL2 | 0.10049 | 0.09027 | 0 | 0 | 0.06813 | 0.09708 | 0.12774 | 0 |
| SHSL4r | 0 | 0 | 0 | 0 | 0 | 0 | 0 | 0 |
| SUCCt2r | 3.35 | 3.35 | -1.71429 | 0 | 3.35 | 3.35 | 3.35 | 0 |
| SUCD1 | 32.04082 | 31.78127 | 0 | 0 | 31.21892 | 31.9543 | 33.21208 | 1.3216 |
| SUCOAS | -924.63241 | -932.16419 | 1.71429 | 0 | -948.48307 | -927.143 | -904.54763 | 1.3216 |
| TALA | 4.54247 | 4.60222 | 0 | 0 | 4.73168 | 4.56239 | 4.38315 | 0 |
| THRD | 0 | 0 | 0 | 0 | 0 | 0 | 0 | 0 |
| THRD_L | 0 | 0 | 0 | 0 | 0 | 0 | 0 | 0 |
| THRS | 0.10993 | 0.09875 | 0 | 0 | 0.07453 | 0.1062 | 0.13974 | 0 |
| TKT1 | 4.54247 | 4.60222 | 0 | 0 | 4.73168 | 4.56239 | 4.38315 | 0 |
| TKT2 | 4.34085 | 4.4211 | 0 | 0 | 4.59498 | 4.3676 | 4.12685 | 0 |
| TPI | 5.36207 | 5.39847 | 2.57143 | 0 | 5.47733 | 5.3742 | 5.265 | 0 |
| TRPAS1 | 0 | 0 | 2.57143 | 0 | 0 | 0 | 0 | 2.3128 |
| TRPS1 | 0.03206 | 0.0288 | 0 | 0 | 0.02173 | 0.03097 | 0.04075 | 0 |
| TYRTA | -0.06539 | -0.05874 | 0 | 0 | -0.04433 | -0.06317 | -0.08312 | 0 |
| UNK5 | 0 | 0 | 0 | 0 | 0 | 0 | 0 | 0 |

**Table S13:** List of Pearson's correlation coefficients (r) between our flux prediction results and $^{13}C$ metabolic flux data.

| Reaction | r | P-value (BH method adjusted) |
|---|---|---|
| Glucose | 0.51 | 0.31 |
| Fructose | 0.87 | 0.04 |
| Gluconate | 0.93 | 0.01 |
| Glycerol | 0.61 | 0.25 |
| Malate | 0.98 | 0 |
| Pyruvate | 0.78 | 0.09 |
| Glutamate | 0.76 | 0.1 |
| Succinate | 0.3 | 0.53 |
| PGI | 0.43 | 0.37 |
| PFK | 0.54 | 0.31 |
| FBP | -0.14 | 0.78 |
| FBA | 0.49 | 0.31 |
| TPI | 0.89 | 0.03 |
| GAPD | 0.78 | 0.09 |
| GAPDi_nadp | NA | NA |
| PGK_1 | 0.73 | 0.11 |
| PYK | 0.64 | 0.22 |
| G6PDH2r | 0.59 | 0.25 |
| GND | 0.52 | 0.31 |
| RPE | 0.48 | 0.31 |
| RPI | -0.57 | 0.28 |
| TKT1 | 0.5 | 0.31 |
| TKT2 | 0.45 | 0.34 |
| TALA | 0.5 | 0.31 |
| PDH | 0.8 | 0.08 |
| CS | 0.6 | 0.25 |
| ICDHyr | 0.55 | 0.3 |
| AKGDH | 0.34 | 0.48 |
| SUCD1 | 0.4 | 0.4 |
| MDH | 0.39 | 0.4 |
| PPCK | 0.96 | 0 |
| ME2 | -0.01 | 1 |
| PC | 0.23 | 0.64 |
| PTAr | 0.81 | 0.08 |
| PGM_1 | 0.73 | 0.11 |
| ENO | 0.73 | 0.11 |
| ACONT | 0.67 | 0.18 |
| FUM | 0.5 | 0.31 |
| ACKr | 0.81 | 0.08 |
| SUCOAS | 0.34 | 0.48 |

**Table S14:** List of Pearson's correlation coefficients (r) between flux prediction results from integrative metabolic analysis tool (iMAT) and $^{13}C$ metabolic flux data.

| **Reaction** | **r** | **P-value (BH method adjusted)** |
|---|---|---|
| Glucose | 0.42 | 0.77 |
| Fructose | 0.64 | 0.34 |
| Gluconate | 1 | 0 |
| Glycerol | 1 | 0 |
| Malate | 0.95 | 0 |
| Pyruvate | NA | NA |
| Glutamate | 0.31 | 0.77 |
| Succinate | 0.65 | 0.34 |
| PGI | 0.26 | 0.8 |
| PFK | 0.44 | 0.77 |
| FBP | NA | NA |
| FBA | 0.5 | 0.7 |
| TPI | -0.29 | 0.77 |
| GAPD | 0.07 | 0.97 |
| GAPDi_nadp | 0.07 | 0.97 |
| PGK_1 | 0.07 | 0.97 |
| PYK | 0.81 | 0.11 |
| G6PDH2r | 0.85 | 0.07 |
| GND | 0.58 | 0.48 |
| RPE | 0.36 | 0.77 |
| RPI | -0.67 | 0.34 |
| TKT1 | 0.73 | 0.22 |
| TKT2 | -0.25 | 0.8 |
| TALA | 0.73 | 0.22 |
| PDH | -0.11 | 0.97 |
| CS | -0.3 | 0.77 |
| ICDHyr | -0.3 | 0.77 |
| AKGDH | 0.13 | 0.97 |
| SUCD1 | 0.38 | 0.77 |
| MDH | 0.31 | 0.77 |
| PPCK | -0.12 | 0.97 |
| ME2 | -0.08 | 0.97 |
| PC | 0.35 | 0.77 |
| PTAr | -0.35 | 0.77 |
| PGM_1 | -0.01 | 1 |
| ENO | -0.01 | 1 |
| ACONT | -0.3 | 0.77 |
| FUM | -0.18 | 0.97 |
| ACKr | -0.35 | 0.77 |
| SUCOAS | 0.13 | 0.97 |

**Table S15:** List of Pearson's correlation coefficients (r) between flux prediction results from metabolic Context-specificity Assessed by Deterministic Reaction Evaluation (mCADRE) and $^{13}C$ metabolic flux data.

| **Reaction** | **r** | **P-value (BH method adjusted)** |
|---|---|---|
| Glucose | -0.22 | 0.87 |
| Fructose | 0.29 | 0.7 |
| Gluconate | -0.49 | 0.95 |
| Glycerol | -0.28 | 0.89 |
| Malate | 0.42 | 0.54 |
| Pyruvate | 0.49 | 0.54 |
| Glutamate | -0.14 | 0.87 |
| Succinate | -0.36 | 0.93 |
| PGI | -0.23 | 0.87 |
| PFK | 0.2 | 0.8 |
| FBP | NA | NA |
| FBA | -0.18 | 0.87 |
| TPI | 0.63 | 0.52 |
| GAPD | 0.5 | 0.54 |
| GAPDi_nadp | 0.06 | 0.87 |
| PGK_1 | 0.41 | 0.54 |
| PYK | 0.72 | 0.52 |
| G6PDH2r | 0.61 | 0.52 |
| GND | -0.03 | 0.87 |
| RPE | -0.12 | 0.87 |
| RPI | -0.12 | 0.87 |
| TKT1 | -0.06 | 0.87 |
| TKT2 | -0.19 | 0.87 |
| TALA | -0.06 | 0.87 |
| PDH | 0.52 | 0.54 |
| CS | -0.1 | 0.87 |
| ICDHyr | -0.1 | 0.87 |
| AKGDH | 0.26 | 0.72 |
| SUCD1 | -0.65 | 0.96 |
| MDH | 0.3 | 0.7 |
| PPCK | NA | NA |
| ME2 | -0.23 | 0.87 |
| PC | -0.16 | 0.87 |
| PTAr | -0.5 | 0.95 |
| PGM_1 | 0.41 | 0.54 |
| ENO | 0.41 | 0.54 |
| ACONT | -0.1 | 0.87 |
| FUM | -0.66 | 0.96 |
| ACKr | -0.5 | 0.95 |
| SUCOAS | 0.62 | 0.52 |

**Table S16:** List of Pearson's correlation coefficients (r) between predicted fluxes from glucose and malate transporters and $^{13}C$ metabolic flux data from the corresponding shifts (glucose to glucose plus malate and malate to malate plus glucose).

| Transporter | Shift | r | P-value (BH method adjusted) |
|---|---|---|---|
| Glucose | Glucose to glucose plus malate | -0.21 | 0.61 |
| Malate | Glucose to glucose plus malate | 0.98 | 0 |
| Glucose | Malate to malate plus glucose | 0.68 | 0.13 |
| Malate | Malate to malate plus glucose | 0.48 | 0.31 |

**Table S17:** Predicted flux from glucose to glucose plus malate shift.

| Reaction | 0 min | 5 min | 10 min | 15 min | 25 min | 45 min | 60 min | 90 min |
|---|---|---|---|---|---|---|---|---|
| 2S6HCCi | 0.00015 | 0.00015 | 0.00016 | 0.00016 | 0.00016 | 0.00017 | 0.00018 | 0.00019 |
| ACKr | -0.62398 | -0.24965 | -0.25199 | -3.68479 | 6.34346 | 2.79099 | 4.28845 | 6.48737 |
| ACONT | 6.95795 | 9.66615 | 8.4681 | 6.15361 | 2.09743 | 5.18786 | 7.24167 | 6.92889 |
| ACOTA | -0.01572 | -0.11195 | -0.03626 | 0 | 0 | 0 | 0 | 0 |
| AKGDH | 9.45798 | 12.07176 | 10.88069 | 6.88673 | 3.9111 | 4.82039 | 5.52526 | 7.08456 |
| AKGt2r | 0 | 0 | 0 | 0 | 0 | 0 | 0 | 0 |
| ALCD19y | 4.38213 | 4.60815 | 3.48494 | 1.24471 | 0.94757 | 0.86846 | -0.00033 | 1.42844 |
| ALDD31_1 | 0 | 0 | 0 | 0 | 0 | 0 | 0 | 0 |
| ARGSL | 0.01572 | 0.11195 | 0.03626 | -0.07318 | -0.06405 | -0.00034 | 0 | 0 |
| ARGabc | 0 | 0 | 0 | 0 | 0 | 0 | 0 | 0 |
| ARGt2r | 0 | 0 | 0 | 0 | 0 | 0 | 0 | 0 |
| ASPO1 | 0 | 0 | 0.21994 | 0 | 0 | 0 | 0 | 0 |
| ASPT | 0.4331 | 0 | 0.00085 | 0.17226 | 0 | 0 | 0 | 0 |
| ASPTA | -1.43208 | -1.46735 | -1.2685 | -1.18695 | -0.98591 | -1.09716 | -1.13145 | -1.20002 |
| CDPDSP_BS | 0.03136 | 0.03248 | 0.03304 | 0.0336 | 0.03416 | 0.03584 | 0.03696 | 0.0392 |
| CITt10 | 0 | 0 | 0 | 0 | 0.06205 | 0 | 0.0199 | 0 |
| CITt14 | 0.00179 | 0.00186 | 0.00189 | 0.00192 | 0.00196 | 0.00205 | 0.00212 | 0.00224 |
| CITt15 | 0 | 0 | 0 | 0 | 0 | 0 | 0 | 0 |
| CITt2r | -0.00566 | -0.00586 | -0.00596 | -0.00606 | -0.06821 | -0.00647 | -0.02657 | -0.00388 |
| CLPNS2_BS | 0.00028 | 0.00029 | 0.00029 | 3e-04 | 3e-04 | 0.00032 | 0.00033 | 0.00035 |
| CS | 6.95795 | 9.66615 | 8.4681 | 6.15361 | 2.09743 | 5.18786 | 7.24167 | 6.92889 |
| CYSS_2 | 0 | 0.00193 | 0 | 0 | 0 | 0 | 0.00315 | 0.00348 |
| CYSTGL_1 | 0.03191 | 0.03113 | 0.03535 | 0.0362 | 0.00936 | 0.01177 | 0.00527 | 0.00784 |

| Reaction | 0 min | 5 min | 10 min | 15 min | 25 min | 45 min | 60 min | 90 min |
|---|---|---|---|---|---|---|---|---|
| CYSTS_2 | 0.03191 | 0.03113 | 0.03362 | 0.03419 | 0.0065 | 0.00991 | 0.00087 | 0.00666 |
| ENO | 17.436 | 17.23856 | 17.23365 | 16.63526 | 17.68918 | 15.33185 | 15.88639 | 15.73647 |
| FRUK | 1.09439 | 1.11185 | 1.40004 | 3.24914 | 1.9776 | 1.66296 | 1.60655 | 0.84837 |
| FBA | 6.1051 | 5.95084 | 6.47289 | 7.71968 | 6.95357 | 6.65307 | 7.38862 | 7.2834 |
| FBA2 | 4.38213 | 4.60815 | 3.48494 | 1.24471 | 2.30984 | 1.31732 | 0.96174 | 1.42844 |
| FBP | 0 | 0 | 0 | 0 | 0 | 0 | 0 | 0 |
| FEDCabc | 0.00193 | 0.002 | 0.00204 | 0.00207 | 0.0021 | 0.00221 | 0.00228 | 0.00082 |
| FRUpts | 5.47652 | 5.72 | 4.88497 | 4.49385 | 4.28744 | 2.98028 | 2.56829 | 2.2768 |
| FUM | 9.40088 | -5.90805 | -10.70194 | -11.8593 | -13.91997 | -11.40044 | -9.74509 | -12.33029 |
| FUMt2r | -0.50591 | -9.6447 | -14.95853 | -13.92145 | -13.85592 | -12.81218 | -12.09518 | -15.81789 |
| G6PDH2r | 0.01817 | 0 | 0.01782 | 0.01424 | 0.22551 | 0.85676 | 0 | 0.01007 |
| GAPD | 17.68403 | 17.3885 | 17.49496 | 16.8469 | 16.55952 | 15.06736 | 15.14183 | 16.0028 |
| GAPDi_nadp | 0 | 0 | 0 | 0 | 0 | 0 | 0 | 0 |
| GHMT2r | 0.06367 | 0.07938 | 0.06708 | 0.01412 | 0.06005 | 0.00023 | 0.03955 | 0.06563 |
| GLCNt2ir | 4.55281 | 4.19215 | 1.54157 | 0.39314 | 0.76929 | 0 | 0 | 2.45726 |
| GLCpts | 6.58297 | 6.43093 | 6.39154 | 4.83195 | 5.65284 | 5.65367 | 6.55534 | 7.16073 |
| GLUDxi | 0.00073 | 0 | 0 | 0 | 0 | 0 | 0 | 0 |
| GLUSy | 0 | 0 | 0 | 0 | 0 | 0 | 0 | 0 |
| GLUt2r | 2.95448 | 3.27964 | 2.91108 | 2.4764 | 1.81886 | 1.00963 | 0.32049 | 0.19431 |
| GLYCt | -2.48524 | -2.87021 | -1.56039 | -0.6845 | -0.41566 | -0.3171 | 0 | -1.23246 |
| GLYK | 1.89717 | 1.73822 | 1.92485 | 0.56051 | 0.53222 | 0.55169 | 0 | 0.19633 |
| GLYO1 | 0 | 0.30378 | 0 | 0 | 0 | 0 | 0 | 0 |
| GNKr | 4.55281 | 4.19215 | 1.54157 | 0.39314 | 0.76929 | 0 | 0 | 2.45726 |
| HISTD | 0 | 0 | 0 | 0 | 0 | 0 | 0 | 0 |
| HISt2r | 0.04577 | 0.04741 | 0.04823 | 0.04904 | 0.04986 | 0.05231 | 0.05395 | 0.05722 |
| HSTPTr | 0 | 0 | 0 | 0 | 0 | 0 | 0 | 0 |
| ICDHyr | 6.95795 | 9.35846 | 8.4681 | 4.8072 | 2.09743 | 3.12391 | 4.57205 | 5.42296 |
| ICITt10 | 0.05696 | 0.05899 | 0.06001 | 0.06103 | 0 | 0.0651 | 0 | 0.0712 |

| Reaction | 0 min | 5 min | 10 min | 15 min | 25 min | 45 min | 60 min | 90 min |
|---|---|---|---|---|---|---|---|---|
| ICITt2 | -0.05696 | -0.36668 | -0.06001 | -1.40743 | 0 | -2.12905 | -2.66962 | -1.57713 |
| LCADi | 0 | 0 | 0 | 0 | 0 | 0 | 0 | 0 |
| LDH_L | -8.81567 | -6.2667 | -8.08622 | -7.39057 | -5.64756 | -5.04366 | -4.79743 | -7.61443 |
| L_LACt2r | -8.81567 | -5.17547 | -6.79947 | -6.24523 | -4.54789 | -3.87338 | -3.62939 | -6.33887 |
| MALt10 | 0 | 1.09123 | 1.28675 | 1.14534 | 1.09967 | 1.17028 | 1.16804 | 1.27555 |
| MALt2r | 0 | 17.45969 | 20.58806 | 18.32551 | 17.59472 | 18.7245 | 18.6886 | 20.40882 |
| MALt4 | 0 | 0.01705 | 0.02011 | 0.0179 | 0.01718 | 0.01829 | 0.01825 | 0.01993 |
| MDH | 8.42766 | 12.6598 | 10.573 | 7.62937 | 3.08334 | 8.34259 | 9.66183 | 9.37375 |
| ME2 | 0.97322 | 0.00012 | 0.61998 | 7e-05 | 1.70826 | 0.17004 | 0.46798 | 0.00027 |
| MCITL2 | 0 | 0 | 0 | 0 | 0 | 0 | 0 | 0 |
| OXGDC | 0 | 0 | 0 | 0 | 0.01178 | 0.01246 | 0 | 0 |
| PC | 0 | 0 | 0 | 0 | 0 | 0 | 0 | 0 |
| PDH | 7.12853 | 10.34941 | 9.45849 | 0 | 9.71609 | 9.254 | 12.88756 | 14.8798 |
| PFK | 5.01071 | 4.83898 | 5.07285 | 4.47055 | 4.97597 | 4.99011 | 5.78207 | 6.43503 |
| PGCD | 0.24802 | 0.30183 | 0.26131 | 0.21164 | 0.2326 | 0.18437 | 0.22323 | 0.26632 |
| GND | 0.65235 | 0.35472 | -1.15582 | 0.40738 | 0.61953 | 0.85676 | -0.58693 | 0.86043 |
| PGI | 6.42389 | 6.28499 | 6.22527 | 4.66673 | 5.27385 | 4.63588 | 6.38927 | 6.97453 |
| PGK_1 | 17.68403 | 17.3885 | 17.49496 | 16.8469 | 16.55952 | 15.06736 | 15.14183 | 16.0028 |
| PGM_1 | 17.436 | 17.23856 | 17.23365 | 16.63526 | 17.68918 | 15.33185 | 15.88639 | 15.73647 |
| PHETA1 | -0.09853 | -0.10204 | -0.1038 | 0 | 0 | 0 | 0 | 0 |
| PPCK | 0.03764 | 1.52631 | 1.05633 | 0.28882 | 0 | 2.05756 | 1.28871 | 1.24483 |
| PPS | 0 | 0 | 0 | 0 | 0 | 0 | 0 | 0 |
| PRAGSr | 0 | 0 | 0 | 0 | 0 | 0 | 0 | 0 |
| PTAr | -0.62398 | -0.24965 | -0.25199 | -3.68479 | 6.34346 | 2.79099 | 4.28845 | 6.48737 |
| PYK | 5.03566 | 6.15891 | 6.61471 | 7.40626 | 7.68647 | 8.68996 | 7.98427 | 7.4725 |
| PYRt2 | 0.31277 | 0.00184 | 0.79048 | -0.19847 | -2.15483 | -1.2904 | 2.07454 | 7.66847 |
| RPE | -0.76896 | -0.87854 | -0.8338 | 0 | 0.36322 | 0.5635 | -0.3914 | 0.47402 |
| RPI | -1.42131 | -1.23326 | 0.32201 | -0.40738 | -0.25631 | -0.29326 | 0.19552 | -0.38641 |

| Reaction | 0 min | 5 min | 10 min | 15 min | 25 min | 45 min | 60 min | 90 min |
|---|---|---|---|---|---|---|---|---|
| SERAT | 0 | 0.00193 | 0 | 0 | 0 | 0 | 0.00315 | 0.00348 |
| SERD_L | 0 | 0 | 0 | 0 | 0 | 0 | 0 | 0 |
| SHSL1_1 | 0 | 0 | 0.00173 | 0.002 | 0.00286 | 0.00186 | 0.0044 | 0.00118 |
| SHSL2 | 0.09538 | 0.09685 | 0.10049 | 0.03419 | 0.0065 | 0.00991 | 0.04018 | 0.07203 |
| SHSL4r | 0 | 0 | 0.0069 | 0.00799 | 0.22223 | 0.14777 | 0.00505 | 0.0047 |
| SUCCt2r | 0 | -8.44706 | -6.66122 | -4.92365 | -3.92288 | -3.42077 | -3.17517 | -3.59696 |
| SUCD1 | 9.45798 | 3.6247 | 4.21947 | 1.96307 | 0 | 1.41208 | 2.35009 | 3.4876 |
| SUCOAS | 9.3626 | 11.9749 | 10.77158 | 6.84254 | 3.67951 | 4.66085 | 5.47563 | 7.00664 |
| TALA | -0.30411 | -0.34027 | -0.33223 | 0.03273 | 0.18169 | 0.28183 | -0.1957 | 0.23701 |
| THRD | 0.16497 | 0.46121 | 0.17381 | 0.23085 | 0.189 | 0.26107 | 0.22992 | 0.22017 |
| THRD_L | 0.18069 | 0.18908 | 0.18175 | 0.18361 | 0 | 0.08344 | 0.24026 | 0.25322 |
| THRS | 0.45 | 0.75835 | 0.46549 | 0.52625 | 0.30266 | 0.46375 | 0.59315 | 0.60381 |
| TKT1 | -0.30411 | -0.34027 | -0.33223 | 0.03273 | 0.18169 | 0.28183 | -0.1957 | 0.23701 |
| TKT2 | -0.46485 | -0.53827 | -0.50158 | -0.03273 | 0.18153 | 0.28166 | -0.1957 | 0.23701 |
| TPI | 12.04467 | 11.94534 | 11.52474 | 9.16091 | 9.42556 | 8.13381 | 7.94996 | 8.4835 |
| TRPAS1 | 0 | 0 | 0 | 0 | 0 | 0 | 0 | 0 |
| TRPS1 | 0 | 0.03151 | 0 | 0 | 0 | 0 | 0 | 0 |
| TYRTA | -0.06206 | -0.06428 | -0.06539 | -0.06531 | 0 | 0 | 0 | 0 |
| UNK5 | 0 | 0 | 0 | 0 | 0 | 0 | 0 | 0 |

**Table S18:** Predicted flux from malate to malate plus glucose shift.

| Reaction | 0 min | 5 min | 10 min | 15 min | 25 min | 45 min | 60 min | 90 min |
|---|---|---|---|---|---|---|---|---|
| 2S6HCCi | 0.00018 | 2e-04 | 2e-04 | 2e-04 | 0.00021 | 0.00021 | 0.00021 | 2e-04 |
| ACKr | 3.68382 | 8.65823 | 9.96326 | 4.90959 | 3.62899 | 4.43707 | 5.79714 | 1.98366 |
| ACONT | 19.77627 | 9.47556 | 5.00971 | 3.53759 | 5.34535 | 6.30409 | 4.2409 | 6.45078 |
| ACOTA | 0 | 0 | 0 | 0 | 0 | 0 | 0 | -0.04712 |
| AKGDH | 17.2819 | 8.32389 | 4.50019 | 4.40913 | 2.72094 | 6.4035 | 5.72492 | 8.03392 |
| AKGt2r | 0 | 0 | 0 | 0 | 0 | 0 | 0 | 0 |
| ALCD19y | -1e-04 | -2.42704 | -1.2518 | -3.54451 | 0.34543 | -0.09934 | 1.93578 | 2.73381 |
| ALDD31_1 | 0 | 0 | 0 | 0 | 0 | 0 | 0 | 0 |
| ARGSL | 0 | 0 | 0 | -0.03963 | 0 | 0 | 0 | 0.04712 |
| ARGabc | 0 | 0 | 0 | 0 | 0 | 0 | 0 | 0 |
| ARGt2r | 0 | 0 | 0 | 0 | 0 | 0 | 0 | 0 |
| ASPO1 | 9.42007 | 0 | 0 | 0 | 0 | 0 | 0 | 0 |
| ASPT | 0 | 0 | 0 | 0 | 0 | 0 | 0 | 0 |
| ASPTA | -10.58581 | -1.2686 | -1.28574 | -1.29479 | -1.33717 | -1.37146 | -1.37146 | -1.37019 |
| CDPDSP_BS | 0.03808 | 0.04144 | 0.042 | 0.04256 | 0.04368 | 0.0448 | 0.0448 | 0.04144 |
| CITt10 | 0 | 0.07527 | 0.04362 | 0 | 0.05844 | 0 | 0 | 0 |
| CITt14 | 0.00218 | 0.00237 | 0.0024 | 0.00244 | 0.0025 | 0.00256 | 0.00256 | 0.00237 |
| CITt15 | 0.07314 | 0 | 0 | 0 | 0 | 0 | 0 | 0 |
| CITt2r | -0.07798 | -0.07961 | -0.91222 | -7.69542 | -6.20235 | -5.48623 | -8.26866 | -8.79829 |
| CLPNS2_BS | 0.00034 | 0.00037 | 0.00038 | 0.00038 | 0.00039 | 4e-04 | 4e-04 | 0.00037 |
| CS | 19.77627 | 9.47556 | 5.87591 | 11.23058 | 11.48588 | 11.78359 | 12.50203 | 15.24273 |
| CYSS_2 | 0.03354 | 0.03738 | 0 | 0 | 0.02027 | 0.03671 | 0.0357 | 0.03556 |
| CYSTGL_1 | 0.00756 | 0.01033 | 0.04888 | 0.04691 | 0.026 | 0.00888 | 0.00989 | 0.00662 |
| CYSTS_2 | 0.00521 | 0.00479 | 0.04274 | 0.04331 | 0.02418 | 0.00888 | 0.00989 | 0.00662 |
| ENO | 9.59383 | 6.6328 | 8.97373 | 9.91146 | 15.14829 | 22.13156 | 20.88579 | 20.53213 |

| Reaction | 0 min | 5 min | 10 min | 15 min | 25 min | 45 min | 60 min | 90 min |
|---|---|---|---|---|---|---|---|---|
| FRUK | 0 | 3.88717 | 2.03185 | 3.55545 | 0 | 0.34267 | 0 | 0 |
| FBA | 7.37731 | 8.13465 | 6.86888 | 9.55843 | 7.97611 | 9.42843 | 9.23039 | 8.64465 |
| FBA2 | 2.29211 | -2.42704 | -1.2518 | -3.54451 | 0.34543 | 0.79376 | 1.93578 | 2.73381 |
| FBP | 0 | 0 | 0 | 0 | 0 | 0 | 0 | 0 |
| FEDCabc | 0.00133 | 0.00098 | 0 | 0 | 0.00044 | 0.00209 | 0.00248 | 0.00198 |
| FRUpts | 2.29211 | 1.46014 | 0.78005 | 0.01094 | 0.34543 | 1.13643 | 1.93578 | 2.73381 |
| FUM | 3.41144 | 1.72089 | -2.41827 | -0.16834 | -4.27666 | -9.71245 | -7.22393 | 1.93676 |
| FUMt2r | 0 | -0.13048 | -2.41827 | -0.12871 | -4.27666 | -9.71245 | -7.22393 | -0.071 |
| G6PDH2r | 0 | 0 | 0.01385 | 0.01567 | 0.0153 | 0 | 0.0262 | 0.02244 |
| GAPD | 7.60279 | 6.96054 | 9.30591 | 10.25615 | 15.49375 | 21.59277 | 21.24011 | 20.85987 |
| GAPDi_nadp | 0 | 0 | 0 | 0 | 0 | 0 | 0 | 0 |
| GHMT2r | 0.07731 | 0.08413 | 0.08527 | 0.0945 | 0.08868 | 0.09095 | 0.09095 | 0.08413 |
| GLCNt2ir | 5.13 | 0.03187 | 0.03881 | 0.53113 | 0.74731 | 3.00118 | 3.4698 | 5.07685 |
| GLCpts | 0 | 7.02089 | 6.63293 | 8.04326 | 8.01793 | 7.92569 | 7.83607 | 8.27177 |
| GLUDxi | 0 | 0 | 0 | 0 | 0 | 0 | 0 | 0 |
| GLUSy | 3.91096 | 0.00021 | 0 | 0 | 0 | 0.00081 | 0 | 0 |
| GLUt2r | 1.24458 | 1.29434 | 0.71483 | 0.42726 | 0.35437 | 0 | 0.0413 | 0.93807 |
| GLYCt | 6.22 | 2.42667 | 1.28276 | 3.54413 | 1.04452 | 1.81432 | -1.49369 | -1.80655 |
| GLYK | 6.22024 | 0 | 0.03133 | 0 | 1.39034 | 1.71538 | 0.44249 | 0.92763 |
| GLYO1 | 0 | 0 | 0 | 0 | 0 | 0 | 0 | 0 |
| GNKr | 5.13 | 0.03187 | 0.03881 | 0.53113 | 0.74731 | 3.00118 | 3.4698 | 5.07685 |
| HISTD | 0 | 0 | 0 | 0 | 0 | 0 | 0 | 0 |
| HISt2r | 0.05558 | 0.06049 | 0.0613 | 0.06212 | 0.06376 | 0.06539 | 0.06539 | 0.06049 |
| HSTPTr | 0 | 0 | 0 | 0 | 0 | 0 | 0 | 0 |
| ICDHyr | 15.60464 | 6.69363 | 3.69413 | 3.53759 | 3.27217 | 4.82025 | 4.2409 | 6.45078 |
| ICITt10 | 0.06917 | 0 | 0.03267 | 0.0773 | 0.0209 | 0.08137 | 0.08137 | 0.07527 |
| ICITt2 | -4.24079 | -2.78193 | -1.34825 | -0.0773 | -2.09408 | -1.56521 | -0.08137 | -0.07527 |
| LCADi | 0 | 0 | 0 | 0 | 0 | 0 | 0 | 0 |

| Reaction | 0 min | 5 min | 10 min | 15 min | 25 min | 45 min | 60 min | 90 min |
|---|---|---|---|---|---|---|---|---|
| LDH_L | 0 | -2.42308 | -2.91405 | -1.75971 | -4.13243 | -8.07493 | -7.07708 | -7.54189 |
| L_LACt2r | 1.65688 | -1.13836 | -1.73954 | -0.57381 | -2.88311 | -6.69171 | -5.69736 | -6.66329 |
| MALt10 | 1.65688 | 1.28473 | 1.17451 | 1.1859 | 1.24932 | 1.38322 | 1.37971 | 0.8786 |
| MALt2r | 24.82724 | 20.55562 | 18.79215 | 18.97439 | 19.98912 | 22.13157 | 22.07543 | 14.0576 |
| MALt4 | 0.02589 | 0.02007 | 0.01835 | 0.01853 | 0.01952 | 0.02161 | 0.02156 | 0.01373 |
| MDH | 29.92107 | 23.58093 | 15.91787 | 20.0101 | 16.98095 | 13.82396 | 16.25232 | 16.88652 |
| ME2 | 0.00037 | 0.00038 | 1.64887 | 0.00038 | 0.00035 | 0 | 0.00045 | 0.00017 |
| MCITL2 | 0 | 0 | 0 | 0 | 0 | 0 | 0 | 0 |
| OXGDC | 0 | 0 | 0.81636 | 0.78268 | 2.15014 | 0 | 0.09922 | 0 |
| PC | 0 | 0 | 0 | 0 | 0 | 0 | 0 | 0 |
| PDH | 24.86325 | 19.72233 | 17.40563 | 17.7356 | 16.77762 | 17.94196 | 20.01946 | 18.8202 |
| PFK | 7.37731 | 4.24747 | 4.83703 | 6.00298 | 7.97611 | 9.08576 | 9.23039 | 8.64465 |
| PGCD | 0.30117 | 0.32774 | 0.33217 | 0.34469 | 0.34546 | 0.35432 | 0.35432 | 0.32774 |
| GND | 2.84281 | -3.50498 | -2.02088 | -2.22849 | 0.7626 | 2.83379 | 3.496 | 1.8628 |
| PGI | 5.98953 | 6.8347 | 6.43037 | 7.83636 | 7.80638 | 7.72439 | 7.60858 | 8.06313 |
| PGK_1 | 7.60279 | 6.96054 | 9.30591 | 10.25615 | 15.49375 | 21.59277 | 21.24011 | 20.85987 |
| PGM_1 | 9.59383 | 6.6328 | 8.97373 | 9.91146 | 15.14829 | 22.13156 | 20.88579 | 20.53213 |
| PHETA1 | 0 | 0 | 0 | -0.13371 | -0.13723 | -0.14075 | -0.14075 | -0.13019 |
| PPCK | 8.97906 | 12.83678 | 8.75623 | 7.48473 | 4.1579 | 0.66891 | 2.37884 | 0.2736 |
| PPS | 0 | 0 | 0 | 0 | 0 | 0 | 0 | 0 |
| PRAGSr | 0 | 0 | 0 | 0 | 0 | 0 | 0 | 0 |
| PTAr | 3.68382 | 8.65823 | 9.96326 | 4.90959 | 3.62899 | 4.43707 | 5.79714 | 1.98366 |
| PYK | 16.21119 | 10.91281 | 10.23599 | 8.82832 | 10.41565 | 13.26457 | 13.10862 | 9.3 |
| PYRt2 | 8.26 | 4.94069 | 3.28096 | 4.90167 | 4.45347 | 6.07254 | 6.59796 | 8.26 |
| RPE | 1.61012 | -2.34526 | -1.34812 | -1.58488 | 0.42477 | 1.62294 | 1.92713 | 0.9309 |
| RPI | -1.23268 | 1.15972 | 0.67277 | 0.6436 | -0.33783 | -1.21084 | -1.56888 | -0.9319 |
| SERAT | 0.03354 | 0.03738 | 0 | 0 | 0.02027 | 0.03671 | 0.0357 | 0.03556 |
| SERD_L | 0 | 0 | 0 | 0 | 0 | 0 | 0 | 0 |

| Reaction | 0 min | 5 min | 10 min | 15 min | 25 min | 45 min | 60 min | 90 min |
|---|---|---|---|---|---|---|---|---|
| SHSL1_1 | 0.00235 | 0.00554 | 0.00614 | 0.00359 | 0.00182 | 0 | 0 | 0 |
| SHSL2 | 0.08227 | 0.08865 | 0.12774 | 0.12944 | 0.11258 | 0.09954 | 0.10055 | 0.09048 |
| SHSL4r | 0 | 0 | 0.23586 | 0.22262 | 0.18303 | 0 | 0 | 0 |
| SUCCt2r | -13.87046 | -6.47252 | -5.31655 | -5.19181 | -4.87109 | -6.4035 | -5.82415 | -6.07328 |
| SUCD1 | 3.41144 | 1.85137 | 0 | 0 | 0 | 0 | 0 | 1.96064 |
| SUCOAS | 17.19728 | 8.22969 | 4.13046 | 4.05348 | 2.42352 | 6.30396 | 5.62437 | 7.94344 |
| TALA | 0.80515 | -1.17253 | -0.6729 | -0.68337 | 0.32433 | 0.90955 | 1.03924 | 0.57165 |
| THRD | 0.20032 | 0.218 | 0.22095 | 0.2158 | 0.22978 | 0.23568 | 0.23568 | 0.218 |
| THRD_L | 0.25061 | 0.27061 | 0 | 0.01902 | 0.08711 | 0.29484 | 0.29384 | 0.27433 |
| THRS | 0.57763 | 0.62649 | 0.36068 | 0.37642 | 0.46222 | 0.67957 | 0.67857 | 0.63021 |
| TKT1 | 0.80515 | -1.17253 | -0.6729 | -0.68337 | 0.32433 | 0.90955 | 1.03924 | 0.57165 |
| TKT2 | 0.80497 | -1.17273 | -0.67521 | -0.90151 | 0.10044 | 0.71339 | 0.88788 | 0.35925 |
| TPI | -0.57841 | 0 | 3.12648 | 1.61367 | 7.41865 | 11.45223 | 11.12333 | 11.85715 |
| TRPAS1 | 0 | 0 | 0 | 0 | 0 | 0 | 0 | 0 |
| TRPS1 | 0 | 0 | 0 | 0 | 0 | 0 | 0 | 0 |
| TYRTA | 0 | 0 | -0.00211 | -0.08423 | -0.08644 | -0.0552 | -0.01039 | -0.08201 |
| UNK5 | 0 | 0 | 0 | 0 | 0 | 0 | 0 | 0 |
